# New *var* reconstruction algorithm exposes high *var* sequence diversity in a single geographic location in Mali

**DOI:** 10.1186/s13073-017-0422-4

**Published:** 2017-03-28

**Authors:** Antoine Dara, Elliott F. Drábek, Mark A. Travassos, Kara A. Moser, Arthur L. Delcher, Qi Su, Timothy Hostelley, Drissa Coulibaly, Modibo Daou, Ahmadou Dembele, Issa Diarra, Abdoulaye K. Kone, Bourema Kouriba, Matthew B. Laurens, Amadou Niangaly, Karim Traore, Youssouf Tolo, Claire M. Fraser, Mahamadou A. Thera, Abdoulaye A. Djimde, Ogobara K. Doumbo, Christopher V. Plowe, Joana C. Silva

**Affiliations:** 1 Division of Malaria Research, Institute for Global Health, University of Maryland School of Medicine, Baltimore, MD USA; 2 Institute for Genome Sciences, University of Maryland School of Medicine, Baltimore, MD USA; 3 Malaria Research and Training Center, University of Science, Techniques and Technologies, Bamako, Mali; 4 Department of Microbiology and Immunology, University of Maryland School of Medicine, Baltimore, MD USA; 5 Department of Medicine, University of Maryland School of Medicine, Baltimore, MD USA

**Keywords:** Malaria, *Plasmodium falciparum*, *Plasmodium falciparum* erythrocyte membrane protein-1, PfEMP1, *var*, *var2csa*, Mali, ETHA

## Abstract

**Background:**

Encoded by the *var* gene family, highly variable *Plasmodium falciparum* erythrocyte membrane protein-1 (PfEMP1) proteins mediate tissue-specific cytoadherence of infected erythrocytes, resulting in immune evasion and severe malaria disease. Sequencing and assembling the 40–60 *var* gene complement for individual infections has been notoriously difficult, impeding molecular epidemiological studies and the assessment of particular *var* elements as subunit vaccine candidates.

**Methods:**

We developed and validated a novel algorithm, Exon-Targeted Hybrid Assembly (ETHA), to perform targeted assembly of *var* gene sequences, based on a combination of Pacific Biosciences and Illumina data.

**Results:**

Using ETHA, we characterized the repertoire of *var* genes in 12 samples from uncomplicated malaria infections in children from a single Malian village and showed them to be as genetically diverse as *var*s from isolates from around the globe. The gene *var2csa*, a member of the *var* family associated with placental malaria pathogenesis, was present in each genome, as were *var*s previously associated with severe malaria.

**Conclusion:**

ETHA, a tool to discover novel *var* sequences from clinical samples, will aid the understanding of malaria pathogenesis and inform the design of malaria vaccines based on PfEMP1.

ETHA is available at: https://sourceforge.net/projects/etha/.

**Electronic supplementary material:**

The online version of this article (doi:10.1186/s13073-017-0422-4) contains supplementary material, which is available to authorized users.

## Background

Naturally acquired immunity to malaria appears to occur, at least in part, through the acquisition of antibodies to parasite antigens expressed on the surface of infected erythrocytes. In epidemiological studies, having antibodies to these parasite-produced erythrocyte surface antigens is consistently associated with protection against clinical malaria [1, 2]. The best studied of these variant surface antigen (VSA) families, *Plasmodium falciparum* erythrocyte membrane protein-1 (PfEMP1) antigens, are large molecules expressed on the surface of the infected erythrocyte [3, 4] that bind to endothelial receptors [5–8]. Each PfEMP1 typically contains between two and eight Duffy-binding-like (DBL) domains and one to two cysteine-rich interdomain regions (CIDR). PfEMP1s are encoded by the *var* family of genes, 40 to 61 of which have been found in the few *P. falciparum* assembled genomes available to date. An infected erythrocyte expresses only one PfEMP1 variant on its surface [9]. *P. falciparum* parasites undergo clonal antigenic variation that is central to their ability to evade the host immune response [10]. PfEMP1s mediate tissue-specific cytoadherence, which allows infected erythrocytes to sequester in small capillaries of targeted host tissue and evade clearance by the spleen, thereby producing devastating pathologic effects [11]. Compared to other *P. falciparum* genes, most *var* genes exhibit extreme diversity, with less than 50% shared amino acid sequence identity [12]. Such diversity has proven a major obstacle to the development of strategies to amplify and sequence new *var* genes from clinical samples, hampering the understanding of *var* gene biology and the role of PfEMP1 in clinical disease. The full complement of *var* genes is only known for a few reference genomes and two clinical samples [13]. Until now, it has thus not been possible to sequence *vars* with adequate completeness and from a number of samples sufficient to associate specific *var* elements with clinical outcomes such as pregnancy-associated or severe malaria [14].

The best characterized PfEMP1, VAR2CSA, is expressed on the surface of infected erythrocytes that bind to chondroitin sulfate in the placental matrix [15, 16]. Antibodies to this antigen prevent placental cytoadherence and are associated with protection from placental malaria [15]. From the limited number of variants sequenced, VAR2CSA exhibits significantly greater sequence conservation than other *var*s, with over 75% shared amino acid identity among orthologs [12, 17, 18], making it a promising subunit vaccine candidate against pregnancy-associated malaria. Better characterization of VAR2CSA diversity within and across different geographic locations would enable an informed assessment of the need for regionally-based vaccines versus a globally effective vaccine approach.

The study of rapidly evolving genes such as those that encode most variant surface antigen families relies on de novo genome assemblies and/or gene-targeted sequencing approaches. Assessment of genetic variation based on mapping of re-sequencing reads requires reads to map uniquely between isolates within a maximum sequence divergence cutoff (usually ~2%, or a maximum of two to three single nucleotide polymorphisms, SNPs, in a 100 bp read). A large proportion of reads originating from *var* loci often cannot be reliably mapped across isolates, either because they map to repetitive regions present in multiple copies in the genome or because they differ among orthologous copies by more than the threshold cutoff necessary for mapping [19]. The long sequence reads generated with Pacific Biosciences (PacBio) technology have great utility as they allow the placement of repetitive regions in an assembly, using flanking unique conserved sequences as a genomic anchor [20, 21]. However, if the PacBio reads are short in comparison to the length of VSA genes, the high error rate of PacBio-generated sequences, which can top 15%, may lead to assembly errors, including chimeras among members of multigene families. In contrast, Illumina sequencing technologies produce much more accurate data, but the short read length poses a challenge for *var* gene assembly.

Here, we describe a novel, specialized pipeline that leverages publicly available *var* gene sequences and capitalizes on a combination of relatively low amounts of PacBio and Illumina data to reconstruct *var* gene sequences present in clinical malaria field samples. We show that all *P. falciparum* genomes from uncomplicated malaria infections harbor *var2csa* and also *var* gene subclasses previously associated with severe malaria. We also show a lack of clustering of VAR2CSA by geography and identify segments conserved across most *var2csa* sequences despite high overall sequence diversity at this locus among all samples analyzed. Such segments, if immunogenic, suggest the potential of a pregnancy-associated malaria vaccine based on a limited number of strains.

## Methods

### Sample collection, genomic DNA preparation, and quantification of host contamination

A total of 3–5 mL of venous whole blood were obtained from 12 children with symptomatic malaria episodes in Bandiagara, Mali, in 2010, as part of a study measuring the incidence of malaria at a vaccine testing site [22]. Whole blood samples were leukocyte-depleted at the time of collection using CF11 columns [23] and then kept frozen at –80 °C until DNA extraction. DNA was extracted using the QIAamp Blood Midi kit, yielding 300 μL of each sample eluted in distilled water. Samples were stored at –80 °C until DNA sequencing. We determined clonality using six neutral microsatellite markers [24, 25]. Host contamination was calculated retrospectively by measuring the proportion of sequenced reads that mapped to the human genome (version GRCh37) (see below). Isolate NF54 was provided by Sanaria, Working Cell Bank SAN02-073009, cultured using standard protocols [26], and DNA extracted as above.

### Generation of Illumina and PacBio whole-genome sequence data and genome assemblies

Genomic DNA libraries for all samples were constructed for sequencing on the Illumina platform using the KAPA Library Preparation Kit (Kapa Biosystems, Woburn, MA, USA). First, 500 ng of DNA was fragmented with the Covaris E210 to about 200 bp. Then libraries were prepared using a modified version of manufacturer’s protocol. The DNA was purified between enzymatic reactions and the size selection of the library was performed with AMPure XT beads (Beckman Coulter Genomics, Danvers, MA, USA). The polymerase chain reaction (PCR) amplification step was performed with primers containing an index sequence six nucleotides in length. Libraries were assessed for concentration and fragment size using the DNA High Sensitivity Assay on the LabChip GX (Perkin Elmer, Waltham, MA, USA). The library concentrations were also assessed by quantitative PCR using the KAPA Library Quantification Kit (Complete, Universal) (Kapa Biosystems, Woburn, MA, USA). The libraries were pooled and sequenced on a 100 bp, paired-end, Illumina HiSeq 2500 run (Illumina, San Diego, CA, USA).

For the Malian clinical samples, DNA was prepared for PacBio sequencing using the DNA Template Prep Kit 2.0 (Pacific Biosciences, Menlo Park, CA, USA). First, DNA was fragmented with the Covaris E210 to generate fragments ~3, 8, or 10 kb in length. Libraries were then prepared per the manufacturer’s protocol. Three SMRT cells were sequenced per library, using P4C2 chemistry and a 120-min movie on the PacBio RS II (Pacific Biosystems, Menlo Park, CA, USA). The process for the generation of NF54 PacBio data was slightly different; NF54 had a longer insert library size (18,800 bp) and was sequenced using P6-C4 chemistry.

Two assemblies were generated for each sample using PacBio-only sequencing data or PacBio plus Illumina data. PacBio-only assemblies were created with Sprai using default settings (http://zombie.cb.k.u-tokyo.ac.jp/sprai/index.html); Sprai by default does not use reads shorter than 2000 bp. Hybrid assemblies using both PacBio and Illumina data were created with Celera assembler, with default settings [27].

### Extraction of var gene-like sequences from whole-genome assemblies

Sequences resembling *var* exon 1 were extracted from the whole-genome assemblies by stitching together lenient amino acid-level alignments to known reference *var* genes. Amino acid alignments were generated using *PROmer* (promer –maxmatch) [28] between each whole-genome assembly and the full set of known exon 1 sequences from 3D7 and VarDom [13]). Matches of at least 50% identity at least 100 bp long were grouped together when they were no more than 100 bp apart, and any such group with a total length of at least 2000 bp was considered a candidate exon 1 sequence. These candidate sequences were then oriented according to the reading frame with the least number of stop codons. Finally, duplicates and subsequences were eliminated by removing any sequence that was covered to at least 90% (proportion of length × proportion of identity) by another.

### Algorithm for var gene reconstruction using ETHA

Likely ends (500 bp) of *var* exon 1 sequences were identified in each PacBio assembly by aligning known exon 1 ends from all *var* genes available in the VarDom database, including the *var* genes from the reference 3D7 genome, against each assembly, using *NUCmer* with option *maxmatch* [28]. Regions of the assembly from the start of each such match to 100 bp past the inferred end of exon 1 were extracted from the assembly and then *kmer*-corrected, using all 71mers observed at least ten times in the Illumina data. These repaired sequences were re-aligned to the reference exon 1 ends, the exact end identified as the rightmost AAGGT occurring in the rightmost 100 bp of the match, and the 71mer ending with that AAGGT was added to the set of identified exon 1 end kmers. A similar approach was used for some other variants including ACCTT (the reverse complement of AAGGT) and other 5mers occurring with low frequency at the end of *var* exon 1. This set of splice site-containing 71mers was then used to start a leftward kmer walk. This iteratively follows each 71mer with a new 71mer which overlaps the previous exactly over 70 shared base pairs, extending it by one base pair.

This process is followed iteratively until no overlapping kmers are found (with an occurrence count of at least ten) or a stop codon is encountered in all reading frames. Kmers at the left-most end of an open reading frame are collected as the starting point for a rightward walk following the same method, with the added stopping condition that a walk will end as soon as a kmer is seen which was in the original set of splice site-containing kmers. These rightward walks are used to identify further splice site kmers by finding the rightmost kmer ending in AAGGT, in each kmer walk terminated by the end of all reading frames. These new splice-site kmers are added to the original set and the leftward walk is repeated, this time using the seeds of the rightward walk as an additional stopping condition. All of the kmers encountered in this final leftward walk are saved as the set of possible 71mers belonging to exon 1. A final leftward walk is carried out to generate all possible sequences that start with the most recent set of splice-site kmers, using only the exon 1 71mers. These sequences are filtered to remove any that is shorter than 1 Kb and the resulting set forms the candidate exon 1 sequences. These candidate exon 1 sequences are aligned to the whole-genome assembly and segments are extracted of at least 500 bp aligning at least 90% identity. The extracted sequences are then assembled using a simple unitigger which requires perfectly matching overlaps.

We then identify all sequences formed by a unique path from the starting kmer and add them to the final set of exon 1 sequences if not already present. Duplicates, defined as sequences fully contained within any exon 1 unitigs, are removed. Also removed are sequences which are completely covered by another sequence with which they share ≥ 95% sequence identity.

### NF54 validation

An optimal NF54 PacBio-only assembly was constructed from all PacBio data using PacBio’s Hierarchical Genome Assembly Process (HGAP) [29]. A second, sub-standard assembly that matched features of the 12 Malian clinical samples was generated from reads subsampled from the total NF54 PacBio data to match the lower median read length and read number of the datasets for Malian clinical samples. This simulated clinical assembly was created with HGAP using the subset of PacBio reads with the whitelist option within SMRT Analysis (v2.3). 71mers used for kmer walk by ETHA were extracted from NF54 Illumina data. The original standard bioinformatic approach and ETHA were then run on this assembly to pull extracted and to generate reconstructed exon 1 sequences, respectively, as described above. For both extracted and reconstructed NF54 output, exon 1 sequences were then compared to the complete set of known exon 1 sequences from the reference 3D7 by alignment using Mummer [28] and coverage and percent identity was calculated for the single best match from the ETHA output to each reference 3D7 exon 1 sequence with custom scripts.

### Amplification and structural annotation of var2csa sequences from genomic DNA

Primers flanking *var2csa*, based on its upstream promoter sequence (UPSE; ~500 bp upstream of translation start site) and on the acidic terminal sequence (ATS), were designed using sequences from GenBank (accession numbers: EF614224; EF614233; EF614230; EF614227; EF614231; EF614225; EF614226; EF614229; EF614232; EF614228) and VarDom (clones 3D7, HB3, DD2, IT4/FCR3, PFCLIN, RAJ116, IGH). UPSE sequences were provided by Thomas Lavstsen [13]. A single PCR was performed using PacBio 96-plex 21 bp padded barcode mode according to manufacturer’s instructions. A target specific forward primer Frag1_ExF (5’-GTGATGTATGTGTTTATGGAATAACTAGC-3’) and a target specific reverse primer R_IT4var04_ATS (5’-TCCTTACGTTCCATATTCCACACTTC-3’) were used. The cycling conditions were as follows: a 50 μL reaction containing 5 μL of 10X LA PCR buffer, 8 μL of dNTPs (2.5 mM each), 5 μL of each the forward and reverse primer (1 μM), 0.5 μL of TaKaRa LA Taq (5 units/μL), and 2 μL of DNA sample. Cycling conditions included an initial denaturation step of 2 min at 94 °C, followed by 10 s at 98 °C denaturation, 10 min annealing and elongation step at 60 °C, with 35 cycles followed by a final elongation step of 10 min at 72 °C. Successful PCR products of 10 kb from each sample were purified and pooled in equimolar concentrations. The pooled amplicons were sequenced using a Single Molecule Real Time (SMRT) RS II amplicon sequencing protocol using P6C4 chemistry, and assembled with SMRT analysis v2.3 ConsensusTools. The VarDom server was used for the *var* gene domain annotation, according to the default protocol parameters.

### Identification of upstream promoter sequence (UPS) for each var gene in the Malian samples

Regions upstream from the translation start positions of *var* genes in our assemblies were extracted for analysis as follows. First, a set of possible exon 1 sequences was created from kmers in Illumina reads, as described above. Then, all PacBio reads were mapped to these sequences using NUCmer and the best match for each PacBio read extracted if it was at least 500 bp long and matched the reconstructed exon 1 with at least 95% sequence identity. The extracted sequences were then assembled in a simple, greedy fashion to create a set of exon 1 unitigs. Those unitigs containing the starts of exon 1 sequences were matched using NUCmer against contigs in the final assembly. The final set of promoter regions consisted of the assembly regions upstream of the exon 1 start of each match (up to 2000 bp but as short as 900 bp if the end of the contig was reached) plus the first 100 bp of exon 1 coding sequence.

To identify the coordinates of each promoter sequence in those upstream regions and the UPS class to which each belonged, a BLAST search was done of known promoter sequences against the *var* gene upstream sequences. Each BLAST hit was then scored to determine the best hits. The scores were determined by adding the length, percent identity, and the bit score of the alignment (inversely proportional to e-value, and of the same order of magnitude and the other two parameters). This scoring takes into account the three factors that most impact the quality of the BLAST hit. The percent identity had little impact on the scores, since nearly all of the hits have a percent identity between 90% and 100%. The coordinates for all the BLAST hits were inferred from the alignments. The best hits were defined by keeping only those hits within 5% of the score of the top hit for each upstream sequence. The 5% threshold represents a natural inflection point in the distribution of hit scores (scores tended to drop off after a few very highly scoring hits; alternatively, scores declined gradually, but represented redundant hits, i.e. alternative alignments of the same promoter to the same target region). All redundant hits were removed from the set of best hits, such that only unique best hits remained. Most often this resulted in only one remaining hit of a “known” promoter against the upstream region of a *var*, which defined the UPS class, and with the alignment coordinates defining the UPS location. In cases where more than one hit remained, it was a result of either: (1) partially overlapping hit coordinates from “known” promoters from the same class; or (2) perfectly overlapping hits, but from “known” promoters of different classes. In (1), the final UPS coordinates were defined by the reunion of all overlapping hits. In (2), the UPS coordinates were common to all hits and the *var* was assigned to a hybrid class defined by all subject “known” promoters. Finally, a small number of *var* sequences appear to have more than one non-overlapping upstream promoter, although these could represent assembly artifacts.

### Identification of domain cassettes DC8 and DC13

DC8 and DC13 containing sequences were retrieved from VarDom server. Each DBL and CIDR domain was identified and aligned separately along with the known DC8 and DC13. Phylogenetic trees were inferred to identify sequences that clustered with either DC8 or DC13 domains. Sequences that contained both DBLα2 and CIDRα1.1 were defined as DC8 whereas those with DBLα1.7 and CIDRα1.4 were DC13.

### Phylogenetic tree and recombination analysis

Multiple sequence alignments were performed with AQUA [30], a program that optimizes multiple alignment using MAFFT and MUSCLE. A maximum likelihood tree was reconstructed with RAxML-PTHREADS (version 8.2.4) [31], based on the AQUA output. The gamma model of substitution with rate heterogeneity was used, together with AUTO option, to determine the matrix of substitution that best fits the data. JTT and VT were identified as the best-scoring amino acid substitution models. The phylogenetic tree was visualized with interactive Tree of Life (iTOL version 3). To determine whether *var2csa* sequences cluster regionally, a multiple alignment was performed using nucleotide sequences with MAFFT L-iNS-i default options followed by careful manual evaluation and editing when required. The alignment was used as an input for recombination analysis using RDP4 version 4.36 [32]. The program detected regions of recombination in the alignment. Therefore, a Neighbor-Net analysis was performed to reconstruct a phylogenetic network incorporating recombination events, using Splitstree version 4.14.4, based on uncorrected p-distances [33].

## Results and discussion

### De novo whole-genome assemblies

Genomic DNA (gDNA) was obtained from leukocyte-depleted blood from each of 12 clinical malaria samples collected in Bandiagara, a town in Mali, West Africa. This set included samples with both low (5000–25,000 parasites/μL) and high (>150,000 parasites/μL) parasitemia, as well as monoclonal and polyclonal infections (Additional file 1: Table S1). Illumina and PacBio data were obtained for all samples (see “Methods”). The insert size of Illumina libraries was in the range of 316–346 bp (Additional file 1: Table S2). The 12 samples were multiplexed in a single Illumina HiSeq2500 channel and data generated corresponded to 78X–141X coverage of the *P. falciparum* genome per sample. A 3Kb, 8Kb, and/or 10Kb PacBio insert library was built for each sample (Additional file 1: Table S2). The total PacBio data, generated through PacBio’s Single Molecule Real Time (SMRT) sequencing, was equivalent to 8X–52X coverage, depending on the sample. The varying amount of data per sample depended on the percent host contamination, the variation associated with multiplexing samples in an Illumina run, the number of PacBio SMRT cells sequenced, and the variation inherent to each SMRT cell (Additional file 1: Tables S1 and S2).

**Fig. 1:**
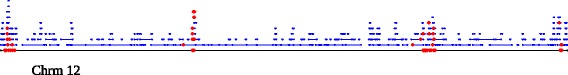
Whole-genome assembly contigs aligned against the 3D7 Reference. Contigs from Sample 303_1 (*blue*) aligned to chromosome 12 of 3D7 (*black*); *var* exon 1 sequences extracted from 303_1 (see “Methods”) and previously identified *var* exon 1 sequences from 3D7 are labeled as *red dots* on the contigs and chromosome. Exon 1 sequences extracted from the whole-genome assembly of 303_1 are found in chromosome regions similar to where 3D7 exon 1 sequences have been found. While some are found towards the center of the chromosome, a portion are also found on the ends, where complicated repeat regions and multi-gene families cause assembly issues and assembly contigs are seen to pile up in this area. Similar layouts for all 14 chromosomes for each of the 12 Malian clinical samples can be found in the Additional files

Assemblies for the 12 Malian clinical samples were created using either Celera (PacBio with Illumina) or Sprai (PacBio-only) assemblers (see “Methods”). In each case, the assembly considered to be best was selected based on various combinations of assembly metrics, including number of contigs, cumulative length of assembly, length of largest contig, and N50 (the length of the largest contig in the subset containing the smallest 50% of all contigs) (Additional file 1: Table S3). The *P. falciparum* assemblies generated from the six monoclonal infections were usually < 30 Mb in length, and consisted of ~450 large contigs (≥10 Kb) of an average maximum size of 325 Kb. These large contigs together had an average cumulative length of 22 Mb (~94% of the reference genome), corresponding to the core, unique regions of the genome (Fig. 1; Additional file 1: Table S3). The assemblies of the six polyclonal samples were all longer than 30 Mb, and the number of large contigs was ~20% greater than those in monoclonal samples, while the number of small (<10 Kb) contigs from the polyclonal samples was roughly twice as big as those from the monoclonal samples, reflecting the presence of multiple allelic forms. All assemblies contained at least 500 small contigs of 2–10 Kb. These small contigs map primarily to telomeric and sub-telomeric regions, suggesting that they correspond to fragments of repeats or the multigene families preferentially located in these regions (Fig. 1; Additional file 2).

The size of all assemblies was larger than the reference 3D7 *P. falciparum* genome. This is likely due to two primary factors. First, the relatively low amount of PacBio data that could be generated for each clinical sample were insufficient to correct all internal errors in the PacBio data. As a result, each assembly likely contains redundant contigs that could not be merged due to incorrectly reconstructed nucleotide positions (Fig. 1). Second, more than one clone was detected in half of these samples; therefore, some of the redundancy in the assembly is likely due to nucleotide-level differences in orthologous regions from multiple parasites. This may explain why most assemblies from polyclonal infections are larger than those from monoclonal infections (Additional file 1: Table S3).

### Extracted *var* gene exon 1 sequences in de novo whole-genome assemblies

Currently available *P. falciparum* genome assemblies contain 40–61 complete *var* genes in addition to several dozen *var* pseudogenes [34]. These genes are mostly located in sub-telomeric regions of the chromosomes, with a few clusters located in the core region of chromosomes 4, 6, 7, 8, and 12 (Fig. 1). Each complete *var* gene consists of two exons, the first of which, at 2500–10,500 bp, is longer and much more variable in length than the second (1000–1500 bp) [34]. While the PfEMP1 segment derived from exon 2 corresponds mostly to the inter-membrane and intra-cellular domains of the protein, exon 1 gives rise to the motifs involved in cytoadherence and rosetting, and consequently is of particular interest in terms of pathogenesis.

**Fig. 2:**
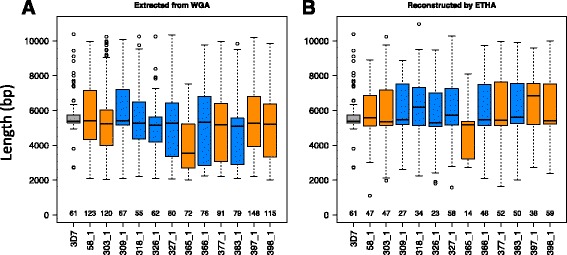
Number and length of *var* exon 1 sequences. **a** Complete exon 1 sequence extracted from whole-genome assemblies using standard bioinformatic methods, requiring a minimum sequence length of 2 Kb. **b** Complete exon 1 sequences reconstructed from whole-genome assemblies by ETHA. Distributions of var exon 1 lengths in each sample are represented by a *box-and-whiskers plot*: the median is indicated by a *dark line*, the first and third quartiles by the *boundaries* of the box, and the minimum and maximum, excluding outliers, by the *whiskers*. Outliers are those points lying beyond the first or third quartile by more than 1.5 times the interquartile range. Clinical samples are colored by clonality (polyclonal: *orange*, monoclonal: *blue*). 3D7 is shown in *gray*. The number of exon 1 sequences per sample, estimated from the counts of extracted exon 1 sequences are listed on the bottom (see “Methods”)

To determine the accuracy of these clinically important exon 1 sequences recovered directly from default assemblies with standard bioinformatics approaches, exon 1 sequences were extracted from each of the 12 Malian whole-genome assemblies based on amino acid sequence similarity to those of existing *var* exon 1 sequences from the reference *P. falciparum* 3D7 (Pf3D7) genome, as well as others available through the VarDom database [13]. The putative exon 1 sequences extracted were validated as bona fide *var* sequences by comparison with the NCBI nr/nt nucleotide collection. Without exception, each sequence was spanned (with or without internal gaps) by at least one match to a known *var*, with a bitscore of at least 200. Between 58 to 177 exon 1 sequences were extracted from each of the 12 Malian sample genome assemblies in this manner, with a higher number from polyclonal versus monoclonal samples (Additional file 1: Table S4). As with Pf3D7 *var* exon 1 sequences, the median length of extracted exon 1 sequences in each assembly was ~5 Kb for most samples, and the length of individual exon 1 sequences fell within a wide range of 2 to 10 Kb (Fig. 2).

Several observations suggest the presence of assembly errors in the *var* exon 1 sequences extracted directly from the assemblies of the 12 Malian clinical samples. First, the number of exon 1 sequences per sample is much larger than the 40–61 expected. This is most likely due to the presence of duplicated segments in the assembly, which could not be merged due to incompatible sequencing errors (see above). Second, some segments in these extracted *var* sequences have no equivalent in the *k*-mer Illumina data, i.e. they have no identical sequence among all the 71mers extracted from all Illumina reads generated for the same sample. Finally, in these extracted *var* sequences with missing *k*-mer data, no independently generated *k*-mer path (tiling paths of 71mers extracted from all Illumina reads) unites the two regions flanking those segments with no *k*-mer coverage. The second observation can be explained by poor PacBio correction, but the final point suggests the presence of larger structural errors, such as chimeras. Therefore, the large number of complete exon 1 sequences extracted directly from these assemblies is inflated by the presence of chimeras, as well as by duplicated copies.

### Reconstruction of *var* gene exon 1 sequences by a novel algorithm, ETHA

**Fig. 3:**
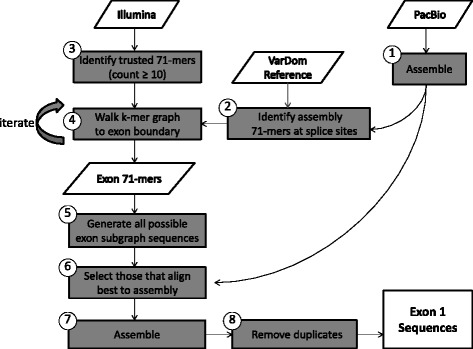
Overview of the ETHA algorithm for reconstructing exon 1 sequences: Illumina and PacBio sequencing data are both used in conjunction with previously characterized exon 1 sequences from VarDom [13] as data inputs for reconstructing exon 1 sequences in clinical whole-genome assemblies. Pacbio data are assembled and exon 1 ends are identified by mapping known exon 1 sequences from VarDom onto the assembly (steps 1 and 2). Illumina data corresponding to *var* genes are identified by finding 71 bp segments (71mers) containing *var* splice site sequences at the end of exon 1 in the assembly and iteratively following possible continuations (new trusted 71mers overlapping previously identified *var* 71mers by 70 bp) within the Illumina data (steps 3 and 4). This process is extended until a start methionine is reached (step 4). This k-mer walk is repeated in the opposite direction, now from the start methionine to the intron. They are then assembled by generating all possible paths within the de Bruijn graph of 71mers (step 5) and reconciled with the whole-genome assembly by choosing those paths which align best with the whole-genome assembly (steps 6–8). Data inputs in *white*; processes are in *gray*. See “Methods” for additional details

Compared to Pf3D7, the *var* exon 1 sequences extracted directly from each of the 12 genome assemblies were extremely variable in length and in number. In addition to the presence of duplicates and chimeras, the length distribution is skewed toward elements smaller than the median length, compared with Pf3D7. For this reason, an algorithm named ETHA was developed to more accurately recover exon 1 sequences (Fig. 3; see “Methods”). Briefly, Illumina data corresponding to the *var* genes is: (1) identified by finding 71 bp segments containing *var* splice site sequences in the assembly and iteratively following possible continuations within the Illumina data; (2) assembled by generating all possible paths within de Bruijn graph of 71mers; (3) reconciled with the whole-genome assembly by choosing paths that align best with the whole-genome assembly; and (4) assembling reconciled *var* sequences in a sample and removing duplicates. While the original extraction method described above was done using the best assemblies, we found that Celera assemblies (while often superior as measured by N50 and other assembly metrics) were less effective than Sprai assemblies as input to the current pipeline, as measured by coverage of known reference *var* genes. Celera Assembler’s double use of Illumina data may explain this reduced coverage. Therefore, the Sprai assemblies were used as input for ETHA.

ETHA reconstructs both complete and partial exon 1 sequences. Those sequences containing a start methionine residue were judged to include the start of an exon; those ending with a known boundary sequence between exon 1 and the intron were judged to include an end. Those with neither were considered internal fragments and those with both were considered complete exon 1 sequences. The median length of complete ETHA-reconstructed exon 1 sequences in each sample was much more similar to Pf3D7 and length variation was far lower than that of sequences extracted by the original method (Fig. 2; Additional file 1: Table S4). Since ETHA includes redundancy reduction steps that will remove nearly identical copies likely to result from sequencing error, the number of complete sequences reconstructed with ETHA is less than what was found with the exon 1 sequences extracted directly from the assemblies, and the total cumulative length of all ETHA exon 1 sequences was closer to what was expected (Additional file 1: Table S5). Finally, the estimated number of copies recovered by ETHA, which can be inferred from the sum of complete elements plus the largest of either the number of exon 1 start or end sequences (Additional file 1: Table S5), is also closer to number of complete elements in a genome than the number of *var* sequences extracted directly from each assembly.

### Validation of ETHA using NF54

To confirm that ETHA is indeed recovering true and accurate *var* exon 1 sequences, we simulated the sequencing, assembly and *var* sequence recovery process used above for the 12 Malian clinical samples now using the *P. falciparum* isolate NF54. NF54 is the isolate from which the reference 3D7 was cloned [35]; therefore, any *var* exon 1 sequences present in NF54 should be very similar to those in the 3D7 genome. Indeed, this was confirmed with the generation of a high-quality PacBio-based whole-genome assembly of NF54, in which each of the 61 3D7 *var*s was present in one intact piece. We found no evidence in the NF54 assembly of extra *var* sequences relative to the original 3D7 assembly, as recently reported in a new PacBio-based 3D7 assembly [21]. The only difference detected between the NF54 and original 3D7 assemblies across the 61 genes was one small indel. The simulation was conducted by subsampling NF54 Illumina and PacBio reads such that the quality metrics for the data used to create the assembly and the ETHA pipeline input were on the low end of the Malian samples, in an attempt to replicate the relatively poor quality of the sequence data generated from the clinical samples (see “Methods”) (Additional file 1: Table S3).

To assess sensitivity, specificity, accuracy, and contiguity of the ETHA algorithm, exon 1 sequences were extracted directly from the simulated NF54 assembly and also  reconstructed with ETHA, as done before for the Malian clinical samples, and aligned to the known exon 1 sequences in the 3D7 reference. Overall, the ETHA output can be characterized as having very high accuracy and specificity at some cost to contiguity and sensitivity, while the direct extractions were quite sensitive and contiguous but at a cost to specificity and accuracy. ETHA output sequences covered 88.4% of the cumulative length of 3D7 *var* exon 1 sequences with alignments of at least 500 bp and 100% identity. Lowering the identity threshold to 90% identity increased the coverage only slightly to 92.8% and lowering it further to 80% achieved only 93.6% coverage. By contrast, the sequences extracted directly from the assembly covered only 17.2% of the cumulative length of the reference at 100% identity, but lowering the threshold to 90% identity increased coverage to 97.3% and lowering it further to 80% achieved 97.8% coverage.

We also calculated coverage and percent identity for the single best match between the two sources. For the ETHA output, 38 of the 61 reference 3D7 exon 1 sequences were covered completely by a single match to reconstructed sequence, all of these with 100% identity. For the sequences extracted directly, only one reference exon 1 was covered completely with 100% identity, but a total of 53 of the 61 were covered completely at lower identities (mean identity of 99.91%, range of 99.58–99.98%). The high number of complete (if not 100% accurate) exon 1 sequences extracted directly was most likely facilitated by the nature of the NF54 simulated sample that could not be degraded sufficiently to resemble true clinical data (likely due to the difference between clinical and cultured parasite genetic material and the longer reads from the updated PacBio chemistry used to sequence NF54; see “Methods”). The ETHA output’s low number of completely covered reference 3D7 exon 1 sequences likely reflects ETHA's conservative nature, which by design will not merge two pieces of sequences unless they are identical across the overlapping region.

### Validation of ETHA using long-range targeted PCR with PacBio sequencing of *var2csa*

A second validation of the ETHA output was done by sequencing *var2csa* with PacBio to compare the PCR amplicon-based sequence to the sequence reconstructed by ETHA. PCR primers spanning the UPS and ATS were used to amplify the extracellular portion of *var2csa*, corresponding to an amplicon around 10 Kb, and then sequence it with PacBio using P6-C4 chemistry (see “Methods”). For all except two samples (397_1 and 398_1), the *var2csa* gene was successfully amplified and sequenced. The ten amplicons were pooled before sequencing with PacBio.

We assembled the PacBio reads with PacBio’s HGAP assembler and further corrected the results using Pilon, with the corresponding Illumina reads [36]. Three of the ten samples (309_1, 366_1, and 383_1) had very low PCR amplicon product and did not yield the desired amount (394.94 ng) to generate an equimolar ten-sample amplicon pool before sequencing. Therefore, the number of PacBio reads for these samples was insufficient to generate a reliable PacBio consensus amplicon sequence. Of the remaining samples, two (318_1 and 377_1) failed to yield high-quality assemblies; for the last five samples, we obtained a single high-confidence contig assembly, thus providing an independent source against which to assess the accuracy of the ETHA pipeline. In four of these five samples (303_1, 326_1, 327_1, and 365_1), the assembled amplicon sequence corresponding to exon 1 was 100% identical, end to end, to the output of the ETHA pipeline, while the fifth (58_1) differed by 11 nucleotides from the ETHA output, corresponding to 99.87% identity. In each of the five samples, the most similar *var2csa*-like sequence extracted directly from the whole-genome assembly differed considerably from the assembled amplicon, with differences in the range of 6–2750 nucleotides (median = 287 bp). This validation exercise provides further evidence that ETHA produces sequences of higher accuracy than those obtained using standard bioinformatic approaches to extract sequences directly from a default, low coverage PacBio assembly.

As a positive control for this validation approach, we also amplified and PacBio-sequenced the exon 1 of *var2csa* from the 3D7 isolate. The sequence obtained was 100% identical to that in the published 3D7 genome assembly.

### Subfamily composition

**Fig. 4:**
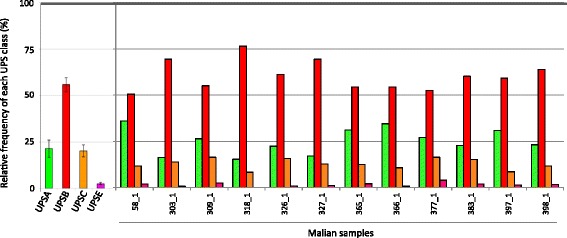
Subfamily composition of recovered ETHA exon 1 sequences: relative frequencies of each UPS class (UPSB in *red*, UPSA in *green*, UPSC in *orange*, and UPSE in *purple*) for each of the 12 Malian samples, compared to the frequencies found by Rask et al. [13]

The upstream regions of *var* genes have previously been classified into four UPS classes, namely UPSA, UPSB, UPSC, and UPSE, which correspond to various chromosomal locations and clinical phenotypes [13]. UPSA (associated with longer *var* genes) and UPSB (the most common) tend to be sub-telomeric, whereas UPSC is located centrally on chromosomes. UPSE is associated only with *var2csa*. To determine if the *var* genes reconstructed from the 12 clinical samples shared a similar promoter breakdown, we extracted 2100 bp-long segments upstream of each exon 1 found in the whole-genome assemblies of the 12 Malian samples, and classified these by sequence similarity to the four UPS classes (see “Methods”). As has been observed in other genomes, UPSB was the most common UPS class for each of the clinical samples (Fig. 4). While previous work found a similar number of UPSA and UPSC classes in a parasite genome [13], UPSA tended to be more numerous than UPSC in the 12 Malian clinical isolates studied here. As expected, UPSE-like sequences were present at very low numbers in each sample. Five samples had between two to four UPSE copies, half of the samples had a single UPSE copy, and none were found in sample 318_1. In contrast, sample 377_1 had 15 UPSE-like sequences. The large number of UPSE sequences relative to the four *var2csa* genes reconstructed with ETHA from the 377_1 assembly likely stems from redundancy in these low coverage PacBio assemblies and sample polyclonality.

### Structural characterization of *var2csa* and other *var*s using VarDom

**Fig. 5:**
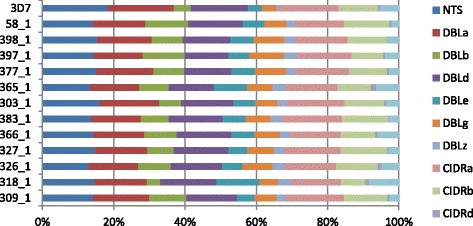
Relative distribution of constitutive domains. Relative domain frequencies compared to the reference 3D7

All ETHA exon 1 sequences were characterized and annotated using the VarDom server ([13]; www.cbs.dtu.dk/services/VarDom/) (see “Methods”). Briefly, the exon 1 genomic sequences were used to predict open reading frames (ORF); the resulting ORFs were translated into protein sequences and submitted to VarDom for domain prediction. The output files were parsed with custom Python and Perl scripts to extract domain coordinates and determine protein domain organization. The overall domain organization was consistent between the samples (Fig. 5; Additional file 3: Figure S1), and similar to the *var* composition in the 3D7 reference genome, with NTS-DBLα-CIDRα-DBLδ-CIDRβ being the most frequent arrangement and NTS-DBLα-CIDRα-DBLδ-CIDRγ the second most common domain architecture. While this domain architecture was conserved, individual PfEMP1 sequences were highly polymorphic. The most conserved *var*s from the repertoire were *var1*, *var3*, and *var2csa.* According to the structural characterization of all samples, *var1*, *var3*, and *var2csa* variants were present in nine, seven, and 12 samples, respectively*.* Complete or nearly complete *var2csa* exon 1 sequences were recovered from all 12 samples, with some samples having more than one *var2csa* sequence, providing cross-validation of the output of the ETHA algorithm. Each sample possessed a similar distribution frequency of constituent domains in these three most widespread *var*s as the 3D7 isolate. The most common domain found in each sequence was DBLα (Additional file 1: Table S6).

PfEMP1s play an important role in malaria pathogenesis by mediating the binding of infected erythrocytes to specific host receptors in different organs. Particular clinical features have been associated with specific domains or the combination of two or more domains defined as domain cassettes (DC). In particular, the expression of DC8 and DC13 has been associated with severe malaria [37]. Previously, DC8 was found in six of seven genomes from different continents, while DC13 was found in five of these genomes [13]. The reference 3D7 genome lacks DC8. To identify these domain cassettes in our dataset, we reconstructed phylogenetic trees using amino acid sequence of domains from ETHA-reconstructed exon 1 sequences as well as amino acid sequences of known DC13 and DC8 domain cassettes. Both domain cassettes were identified in 11 samples at least once (Additional file 3: Figures S2 and S3). Samples 58_1 and 397_1 had more than one DC8 (paired domains). We detected neither DC8 nor DC13 in sample 318. These results suggest that differences in clinical outcome may be the result of variation in *var* expression and/or host immunity, instead of being directly related to domain composition of the *var* complement in the genome. These findings may also be explained by other factors such as the timing of sample collection, the possibility that the DC8s and DC13s sequenced in these samples differ, in some critical way, from DC8s and DC13s that have been associated with severe malaria, or that these uncomplicated malaria infections could have progressed to severe infections if left untreated.

### Genetic diversity of *var2csa* and other *var*s

To characterize the diversity of *var* repertoires, average amino acid identity was calculated for each domain of the major DBL and CIDR classes, as well as the entire exon 1 region of *var2csa*. Among a sample of *var*s obtained from a collection of isolates from around the globe, amino acid sequence identity of individual domains was in the range of 30–92% [13]. The DBLε (31% shared amino acid identity) and CIDRα domain (30% shared amino acid identity) classes were the most heterogeneous domains, whereas DBLβ and CIDRδ were relatively more conserved, with 43% and 55% shared amino acid identity, respectively (Additional file 1: Table S7). Remarkably, both the relative sequence conservation among domains and the range of amino acid sequence identity observed among 12 Malian samples are essentially identical to global isolates (Additional file 1: Table S7). We also confirmed that VAR1 and VAR3 were among the most conserved *var*s, with 67% and 87% shared amino acid identity, respectively. The high level of conservation of these two proteins among parasites suggests particular—and as yet undiscovered—niches in malaria pathogenesis that warrant further investigation.

The genetic variation among VAR2CSA proteins is particularly pertinent to the development of a pregnancy-associated malaria vaccine. We identified and characterized the genetic diversity among the 19 different *var2csa* sequences found in the 12 Malian samples. The average shared amino acid sequence identity among these sequences was 79%. Sequence identity within the individual constituent domains ranged from 62% (DBLε10) to 92% (DBLεpam4). DBLεpam4 was the most homogeneous domain, followed by DBLεpam5 and DBLpam3, both with 89% amino acid sequence identity (Additional file 1: Table S7). The ID1–ID2a region of the VAR2CSA protein, which contains the motif that binds the CSA binding region, is the target of current efforts to develop a vaccine against malaria in pregnancy. The average amino acid sequence identity for this region was 74%, suggesting that it has considerable heterogeneity even among parasites from a single West African village. In addition, all 19 Malian ID1-ID2a sequences were unique.

**Fig. 6:**
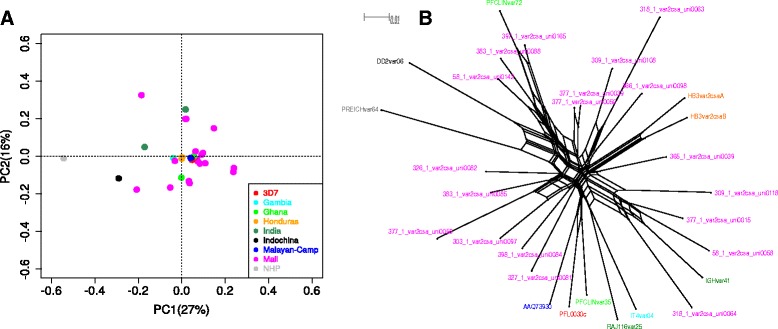
Sequence similarity among *var2csa* elements. **a** PCA of reconstructed and known *var2csa* exon 1 amino acid sequences: 19 *var2csa* sequences reconstructed from the 12 Malian samples were aligned to VarDom *var2csa* sequences and Euclidian distance matrix generated from the multiple alignment was used to plot the PCA. The sequences are colored based on their origin. 3D7, FCR3/IT4 (Gambia), HB3 (Honduras), Dd2 (Indochina), PFCLIN (Ghana), IGH (India), RAJ116 (India), AAQ73930 (Malayan Camp), and NHP (*Plasmodium reichenowi*, a **N**on-**H**uman **P**rimate parasite *var2csa*). **b** Neighbor net of the reconstructed *var2csa* exon 1 nucleotide sequences based on uncorrected p-distances. The network is inferred from the 19 *var2csa* sequences reconstructed from the 12 Malian samples and 11 *var2csa* sequences from the VarDom database multiple alignments. The sequences are color-coded based on their origin as in Fig. 6a

To determine the relatedness among *var2csa* sequences from parasites collected in different geographic locations, Malian sequences were aligned and compared to *var2csa* sequences retrieved from the VarDom database, which were obtained from parasites from across the globe. A principal component analysis revealed that *var2csa* sequences from Mali did not form a distinct cluster; instead, Malian samples displayed a large range of genetic diversity in the principal component analysis (PCA) (Fig. 6a). Supporting this conclusion, phylogenetic analyses showed Malian *var2csa* sequences not to be monophyletic; in fact, several Malian sequences were more closely related to *var2csa*s from other continents than they were to other Malian samples (Fig. 6b). The phylogenetic network was reconstructed to account for recombination, since recombination events were detected in the data, both among Malian samples and between these and other samples (Additional file 3: Figure S4). An analysis under the assumption of no recombination differed slightly in topology but still showed Malian samples to be polyphyletic in the network (Additional file 3: Figure S5). These results, taken together, suggest that VAR2CSA sequences are not structured according to geography, supporting the idea that a VAR2CSA-based vaccine based on a number of strains within a region would still capture considerable global *var2csa* sequence diversity.

Despite the tremendous variation among *var2csa* sequences from a single Malian village, two VAR2CSA C-terminal extracellular domains, DBLεpam4 and DBLεpam5, were remarkably conserved among these isolates. In a recent study using the 3D7 VAR2CSA variant [38], sera from multigravid women from Mali recognized a fragment containing these two domains to a greater extent than sera from nulliparous women, men, and children. Other studies have also shown that these domains were targets of maternal antibodies [39–41]. Our results suggest that the high sequence conservation of the extracellular C-terminal makes it a potential target for a subunit vaccine. Immune responses and allele-specific immunogenicity against different variants of these domains are important to characterize, as previous studies suggest that a repertoire of antibodies from women recognize both conserved and polymorphic epitopes [42].

## Conclusions

Sequence analysis of PfEMP1s from clinical isolates has the potential to provide insights into malaria pathogenesis and vaccine design. Challenges in sequencing *var* genes from clinical isolates have limited our understanding of *P. falciparum* pathogenesis and ability to develop interventions focused on this family of variant proteins. We overcame these challenges by combining two sequencing technologies and developing and validating a novel assembly algorithm. PacBio offered the advantage of long reads, permitting assembly of *var*s, while Illumina corrected the inherent sequencing errors associated with PacBio. ETHA is a novel algorithm for reconstructing variable *var* exon 1 sequences from unfinished, highly fragmented whole-genome assemblies generated from clinical samples using PacBio and Illumina data. This approach allowed us to accurately and reproducibly reconstruct the sequence of full-length *var* exon 1 from clinical isolates, in an automated fashion, for the first time. While *var* sequences extracted directly from the assemblies likely encompass almost all *var* exon 1 sequences present in the sample, collection of sequences was both chimeric and redundant, and contained sequencing errors. In contrast, ETHA removed much of this duplication and reduced chimerism and sequencing errors, albeit with a slight cost in sensitivity and contiguity, resulting in some fragmentation. The accuracy of ETHA *var* reconstruction was validated using several approaches, including the recovery of known *var*s and the validation of the motif composition of novel reconstructed *var*s.

This approach will provide valuable *var* sequence information for clinical infections, with the potential to vastly expand the limited available sequence data from the field, with associated clinical and epidemiological data. The pipeline described here can be adapted to other surface variant proteins such as those encoded by the *r*epetitive *i*nterspersed *f*amily (*rif*) and the *s*ub-*te*lomeric *v*ariable *o*pen *r*eading frame (*stevor*) gene families, which have yet to be sequenced from clinical infections.

This approach has some limitations. Sequence generation is dependent on the acquisition of adequate quantities of venous blood (typically 3–5 mL) with a sufficient parasitemia to guarantee enough parasite DNA for at least a small insert PacBio library—here, a minimum of 5000 parasites per microliter. In addition, the cost of necessary next-generation sequencing may be prohibitive for some projects. Finally, these blood samples required leukocyte depletion in the field to reduce host DNA contamination. New developments to deplete human DNA from mixed host-parasite DNA samples, coupled with sequencing technology advances to reduce costs and improve sensitivity may soon overcome some of these limitations.

Comparative sequence analysis showed that the repertoire of *var* genes found in a small number of isolates from a single West African village was as variable as a collection of isolates from different continents. However, two immunogenic VAR2CSA C-terminal regions of exon 1 [38–40] exhibited considerable sequence conservation, suggesting a potential target for vaccine development. Additional *var* sequence data from different geographic settings would serve to accurately determine epitope region variability. Such information would allow better representation of PfEMP1 diversity on protein and peptide microarrays used to assay malaria exposure and immunity [38, 43–45].

We have shown that parasite sequences present in uncomplicated malaria infections included *var2csa*s and also *var* subtypes associated with severe malaria. Severity of clinical infection was thus not dictated by the potential for virulence of the *var* repertoire present in an infection. The presence of DC8 and DC13 in the genomes of parasites in uncomplicated malaria infections contradicts a hypothesis that severe malaria is caused by highly restricted, virulent strains of *P. falciparum* [46], even though some caveats to this point remain, as mentioned in “Results.” Additional studies of host immunity, specific *var* gene regulation and expression, and *var* gene subsets in different clinical syndromes are needed to better understand determinants of virulence.

## Additional files

Additional file 1: A file containing Supplementary Tables S1–S7. **Table S1:** Samples and respective metadata. **Table S2:** Genomic DNA: Illumina and PacBio Sequencing Statistics. **Table S3:** Assembly characteristics for 12 Malian samples generated by Sprai and Celera assemblers. **Table S4:** Exon 1 *var* sequences recovered with ETHA compared to raw *var*-like sequences extracted directly from assemblies. **Table S5:** Percent of extracted sequence absent from the ETHA output. **Table S6:** Comparison of PfEMP1 constitutive domains reconstructed from 12 samples to the reference 3D7. **Table S7:** Average percent (%) amino acid identity within PfEMP1 constitutive domains. (DOCX 64 kb)

Additional file 2: Contig pile-ups of the genome of 12 *P. falciparum* isolates from Mali aligned against the reference 3D7 genome. The genome assembly of each of the 12 isolates, represented by its constituent contigs (*blue lines* in between *blue circles*) is aligned against each of the 14 nuclear chromosomes of the reference *P. falciparum* 3D7 strain (*black*). The location of *var* gene sequences is shown (*red*) in both the 3D7 genome and the contigs aligned to it. (PDF 1608 kb)

Additional file 3: A file containing Supplementary Figures S1–S5. **Figure S1:** Domain organization of PfEMP1 in each of 12 Malian isolates. **Figure S2:** Maximum likelihood phylogeny of DBLα domain sequences. **Figure S3:** Maximum likelihood phylogeny of CIDRα domain sequences. **Figure S4:** Detection and visualization of recombination events within and between *var2csa* sequences. **Figure S5:** Phylogenetic tree of reconstructed *var2csa* exon 1 sequences. (DOCX 3594 kb)

## Abbreviations

ATS: Acidic terminal sequence
CIDR: Cysteine-rich interdomain region
DBL: Duffy-binding-like
DC: Domain cassettes
ETHA: Exon-Targeted Hybrid Assembly
HGAP: Hierarchical Genome Assembly Process
ORF: Open reading frame
PacBio: Pacific Biosciences
PfEMP1: *Plasmodium falciparum* erythrocyte membrane protein-1
SMRT: Single Molecule Real Time
UPS: Upstream promoter sequence
