# Supplementary material for: New var reconstruction algorithm exposes high var sequence diversity in a single geographic location in Mali
Source: Genome Med. 2017 Mar 28;9:30. doi: 10.1186/s13073-017-0422-4 (PMC5368897; doi:10.1186/s13073-017-0422-4)
Supplement: Supplementary file 1 — A file containing Supplementary Tables S1–S7. Table S1: Samples and respective metadata. Table S2: Genomic DNA: Illumina and PacBio Sequencing Statistics. Table S3: Assembly characteristics for 12 Malian samples generated by Sprai and Celera assemblers. Table S4: Exon 1 var sequences recovered with ETHA compared to raw var-like sequences extracted directly from assemblies. Table S5: Percent of extracted sequence absent from the ETHA output. Table S6: Comparison of PfEMP1 constitutive domains reconstructed from 12 samples to the reference 3D7. Table S7: Average percent (%) amino acid identity within PfEMP1 constitutive domains. (DOCX 64 kb) [file 13073_2017_422_MOESM1_ESM.docx]

**Table S1. Samples and respective metadata.** Samples were collected in Bandiagara, Mali. Samples display a range of high (>150k parasites/µL) and low (<25k parasites/µL) parasitemia, and mono- and polyconality. Parasitemia, clonality and percent host contamination were estimated as descibed in Methods.

| **Plasmodium sample ID** | **Parasitemia**  **(parasites/µL)** | **Clonality** | **gDNA**  **(ng)** | **Host Contamination (%)** |
| --- | --- | --- | --- | --- |
| 58_1 | 209,050 | Polyclonal | 18,024 | 45.1 |
| 303_1 | 15,400 | Polyclonal | 1,434 | 9.6 |
| 309_1 | 185,400 | Monoclonal | 9,804 | 28.2 |
| 318_1 | 23,400 | Monoclonal | 3,906 | 6.9 |
| 326_1 | 7,525 | Monoclonal | 1,344 | 28.9 |
| 327_1 | 193,800 | Monoclonal | 16,608 | 6.1 |
| 365_1 | 10,725 | Polyclonal | 1,110 | 44.5 |
| 366_1 | 19,275 | Monoclonal | 2,448 | 9.7 |
| 377_1 | 198,900 | Polyclonal | 16,320 | 9.3 |
| 383_1 | 220,800 | Monoclonal | 2,658 | 13.8 |
| 397_1 | 151,200 | Polyclonal | 7,092 | 19.8 |
| 398_1 | 14,650 | Polyclonal | 2,622 | 25.3 |

**Table S2. Genomic DNA: Illumina and PacBio Sequencing Statistics**

| **Sample ID** | **Illumina HiSeq** | | | **PacBio** | | | | | |
| --- | --- | --- | --- | --- | --- | --- | --- | --- | --- |
|  | **Library Insert Size (bp)** | **Total Bases** | **Genome Coverage*^a^*** | **Insert Library** | **No. SMRT Cells** | **All Reads** | | **Subset, Reads ≥ 2Kb** | |
|  |  |  |  |  |  | **Total Bases** | **Genome coverage*^b^*** | **Total bases** | **Genome coverage*^b^*** |
| 58_1 | 336 | 6,248,408,026 | 78X | 3Kb; 8Kb | 10 | 1,271,314,933 | 16X | 962,086,036 | 12X |
| 303_1 | 326 | 5,813,460,212 | 133X | 10Kb | 10 | 482,041,714 | 11X | 388,644,760 | 9X |
| 309_1 | 334 | 5,832,635,264 | 101X | 10Kb | 8 | 1,116,447,517 | 19X | 906,086,485 | 16X |
| 318_1 | 339 | 5,760,398,448 | 129X | 3Kb; 8Kb | 8 | 918,464,230 | 21X | 728,573,003 | 16X |
| 326_1 | 321 | 6,031,789,084 | 103X | 10Kb | 8 | 920,052,939 | 16X | 709,223,990 | 12X |
| 327_1 | 340 | 5,926,111,370 | 140X | 10Kb | 8 | 929,381,160 | 22X | 706,674,360 | 17X |
| 365_1 | 324 | 6,521,539,498 | 79X | 10Kb | 10 | 693,315,649 | 8X | 546,209,835 | 7X |
| 366_1 | 320 | 5,791,669,866 | 131X | 10Kb | 8 | 332,256,386 | 12X | 439,544,789 | 10X |
| 377_1 | 338 | 5,903,858,242 | 141X | 10Kb | 10 | 1,647,976,264 | 39X | 1,354,769,485 | 32X |
| 383_1 | 326 | 8,017,999,130 | 110X | 10Kb | 8 | 565,993,711 | 8X | 448,930,861 | 6X |
| 397_1 | 316 | 5,216,390,026 | 101X | 10Kb | 10 | 1,734,269,009 | 34X | 1,398,696,113 | 27X |
| 398_1 | 346 | 5,576,348,168 | 103X | 10Kb | 10 | 2,796,195,673 | 52X | 2,183,535,333 | 40X |
| NF54 | 403 | 3,977,840,560 | 169X | 18.8Kb | 4 | 4,146,771,099 | 176X | 4,079,004,160 | 173X |

***^a^*** Cumulative length of reads mapped to the Pf genome assembly divided by Pf genome size.

***^b^*** Estimated coverage with PacBio, given the relationship between cumulative base pair and genome coverage obtained for Illumina HiSeq data that mapped to the Pf, for the same sample.

| **Table S3. Assembly characteristics for 12 Malian samples generated by Sprai and Celera assemblers.** For each sample, the “best” assembly (shown in bold) was submitted to NCBI. | | | | | | | | | | | |
| --- | --- | --- | --- | --- | --- | --- | --- | --- | --- | --- | --- |
| **Sample** | **Clonality** | **Platform** | **Assembler** | **No. Contigs** | **Total Length** | **Contig Coverage** | **No. Contigs >= 1kb** | **No. Contigs >= 10kb** | **Largest Contig** | **GC (%)** | **N50** |
| 58_1 | Poly | PacBio | Sprai | 3,923 | 20,133,304 | 6.00 | 3,923 | 318 | 44,308 | 20.3 | 5,911 |
|  |  | **PacBio+Illumina** | **Celera** | **2,532** | **31,914,281** | **9.76** | **2,532** | **530** | **271,026** | **20.2** | **41,929** |
| 303_1 | Poly | PacBio | Sprai | 5,338 | 36,846,085 | 4.82 | 5,338 | 836 | 59,629 | 20.0 | 7,907 |
|  |  | **PacBio+Illumina** | **Celera** | **3,310** | **32,088,274** | **8.15** | **3,310** | **677** | **164,654** | **20.2** | **23,330** |
| 309_1 | Mono | PacBio | Sprai | 989 | 23,175,876 | 11.57 | 989 | 658 | 171,375 | 19.6 | 37,098 |
|  |  | **PacBio+Illumina** | **Celera** | **1,754** | **27,761,916** | **8.84** | **1,754** | **508** | **232,970** | **20.0** | **49,431** |
| 318_1 | Mono | **PacBio** | **Sprai** | **1,448** | **28,151,628** | **18.39** | **1,448** | **297** | **405,578** | **19.8** | **128,734** |
|  |  | PacBio+Illumina | Celera | 1,315 | 26,753,146 | 13.09 | 1,315 | 327 | 404,366 | 19.9 | 86,651 |
| 326_1 | Mono | PacBio | Sprai | 4,714 | 36,519,655 | 10.25 | 4,714 | 810 | 107,245 | 19.7 | 13,238 |
|  |  | **PacBio+Illumina** | **Celera** | **3,555** | **31,920,880** | **9.12** | **3,555** | **599** | **157,437** | **20.4** | **22,357** |
| 327_1 | Mono | **PacBio** | **Sprai** | **3,144** | **35,519,348** | **13.91** | **3,144** | **513** | **287,227** | **20.0** | **41,276** |
|  |  | PacBio+Illumina | Celera | 3,942 | 38,228,756 | 9.14 | 3,942 | 730 | 196,515 | 19.7 | 21,243 |
| 365_1 | Poly | PacBio | Sprai | 4,081 | 21,851,385 | 4.23 | 4,081 | 358 | 33,632 | 20.8 | 5,951 |
|  |  | **PacBio+Illumina** | **Celera** | **9,261** | **31,652,294** | **2.41** | **9,261** | **240** | **24,589** | **21.1** | **3,947** |
| 366_1 | Mono | **PacBio** | **Sprai** | **1,322** | **28,206,277** | **13.42** | **1,322** | **366** | **591,160** | **19.8** | **107,945** |
|  |  | PacBio+Illumina | Celera | 3,378 | 36,502,528 | 7.46 | 3,378 | 815 | 171,916 | 19.8 | 19,374 |
| 377_1 | Poly | **PacBio** | **Sprai** | **2,025** | **37,201,405** | **13.86** | **2,025** | **848** | **822,855** | **20.0** | **38,917** |
|  |  | PacBio+Illumina | Celera | 1,542 | 32,078,753 | 13.43 | 1,542 | 496 | 568,516 | 19.7 | 96,135 |
| 383_1 | Mono | **PacBio** | **Sprai** | **1,733** | **29,490,227** | **11.48** | **1,733** | **461** | **276,389** | **19.7** | **56,985** |
|  |  | PacBio+Illumina | Celera | 3,653 | 41,425,376 | 6.38 | 3,653 | 1,050 | 234,774 | 19.9 | 14,177 |
| 397_1 | Poly | PacBio | Sprai | 4,524 | 52,612,787 | 10.65 | 4,524 | 1,499 | 407,419 | 20.1 | 13,401 |
|  |  | **PacBio+Illumina** | **Celera** | **2,781** | **37,773,347** | **11.48** | **2,781** | **587** | **645,552** | **20.4** | **44,226** |
| 398_1 | Poly | **PacBio** | **Sprai** | **876** | **30,162,044** | **17.72** | **876** | **370** | **1,114,782** | **20.1** | **230,653** |
|  |  | PacBio+Illumina | Celera | 1,873 | 32,005,636 | 13.07 | 1,873 | 438 | 578,983 | 20.4 | 75,247 |
| NF54: Original | N/A | PacBio | HGAP | 42 | 23,761,894 | 12.31 | 42 | 38 | 3,331,393 | 19.5 | 1,539,554 |
| NF54: SCS |  |  |  | 1,313 | 21,601,209 | 24 | 1,262 | 692 | 168,463 | 19.6 | 28,924 |

**Table S4. Exon 1 *var* sequences recovered with ETHA compared to raw *var*-like sequences extracted directly from assemblies.** The reference 3D7 isolate had a total of 61 complete elements.

| **Sample** | **Clonality** | **No. extracted seqs *^a^*** | **No. ETHA seqs *^b^*** | **No. with start seq *^c^*** | **No. with internal seq only *^d^*** | **No. with end seq *^e^*** | **Complete elements *^f^*** | **Estimated no. elements *^g^*** |
| --- | --- | --- | --- | --- | --- | --- | --- | --- |
| 58_1 | Poly | 136 | 126 | 32 | 23 | 24 | 47 | 79 |
| 303_1 | Poly | 164 | 82 | 8 | 17 | 10 | 47 | 57 |
| 309_1 | Mono | 75 | 103 | 20 | 22 | 34 | 27 | 61 |
| 318_1 | Mono | 58 | 54 | 6 | 2 | 12 | 34 | 46 |
| 326_1 | Mono | 71 | 71 | 17 | 8 | 23 | 23 | 46 |
| 327_1 | Mono | 104 | 82 | 6 | 3 | 15 | 58 | 73 |
| 365_1 | Poly | 124 | 81 | 18 | 28 | 21 | 14 | 35 |
| 366_1 | Mono | 96 | 87 | 12 | 5 | 22 | 48 | 70 |
| 377_1 | Poly | 120 | 86 | 10 | 14 | 10 | 52 | 62 |
| 383_1 | Mono | 100 | 85 | 8 | 3 | 24 | 50 | 74 |
| 397_1 | Poly | 177 | 124 | 27 | 30 | 29 | 38 | 77 |
| 398_1 | Poly | 154 | 78 | 4 | 2 | 13 | 59 | 72 |

***^a^*** Total number of *var*-like sequences extracted from each assembly. ***^b^*** Total number of *var*-like sequences reconstructed with ETHA for each sample, which can be complete or partial exon 1s. ***^c^*** Total number of *var*-like ETHA sequences containing the start of exon 1. ***^d^*** Total number of *var*-like ETHA sequences without the start or the end of exon 1. ***^e^*** Total number of *var*-like ETHA sequences containing the end of exon 1. ***^f^*** Total number of *var*-like ETHA sequences containing both the start and the end of exon 1. ***^g^*** Estimated number of elements is the sum of the number of complete elements plus the largest of either the number of elements with a start or an end sequence.

**Table S5: Percent of Extracted Sequence absent from the ETHA output.** The cumulative length of *var* exon 1 sequences in the 3D7 genome is 330,105 bp.

| **Sample** | **Clonality** | **% not in ETHA***^a^* | **Extracted Sequence** | | **ETHA Sequence** | |
| --- | --- | --- | --- | --- | --- | --- |
|  |  |  | **No. Sequences** | **Cumulative Length (bp)** | **No. Sequences** | **Cumulative Length (bp)** |
| 58_1 | Poly | 29.33 | 136 | 734,851 | 126 | 524,229 |
| 303_1 | Poly | 53.20 | 164 | 784,187 | 82 | 365,652 |
| 309_1 | Mono | 22.14 | 75 | 424,891 | 103 | 407,285 |
| 318_1 | Mono | 10.83 | 58 | 305,804 | 54 | 286,549 |
| 326_1 | Mono | 17.00 | 71 | 344,918 | 71 | 293,303 |
| 327_1 | Mono | 7.87 | 104 | 487,163 | 82 | 441,858 |
| 365_1 | Poly | 35.85 | 124 | 457,135 | 81 | 242,247 |
| 366_1 | Mono | 8.05 | 96 | 471,496 | 87 | 445,258 |
| 377_1 | Poly | 27.49 | 120 | 571,498 | 86 | 430,018 |
| 383_1 | Mono | 8.21 | 100 | 452,088 | 85 | 447,930 |
| 397_1 | Poly | 40.13 | 177 | 903,806 | 124 | 485,891 |
| 398_1 | Poly | 39.07 | 154 | 693,912 | 78 | 427,658 |

*^a^* The absence of some sequences from the ETHA output is due to a combination of the higher sensitivity, higher redundancy, and higher error rate of the extracted sequences.

**Table S6: Comparison of PfEMP1 constitutive domains reconstructed from 12 samples to the reference 3D7**

| Sample | NTS | DBLα | DBLβ | DBLδ | DBLε | DBLγ | DBLζ | CIDRα | CIDRβ | CIDRδ | CIDRγ | Clonality |
| --- | --- | --- | --- | --- | --- | --- | --- | --- | --- | --- | --- | --- |
|  |  |  |  |  |  |  |  |  |  |  |  |  |
| 309_1 | 49 | 55 | 36 | 49 | 17 | 21 | 9 | 57 | 42 | 2 | 9 | Mono |
| 318_1 | 39 | 39 | 10 | 42 | 32 | 14 | 10 | 37 | 18 | 3 | 22 | Mono |
| 326_1 | 40 | 43 | 28 | 44 | 18 | 26 | 10 | 45 | 36 | 3 | 15 | Mono |
| 327_1 | 58 | 57 | 29 | 60 | 21 | 29 | 11 | 62 | 52 | 1 | 11 | Mono |
| 366_1 | 55 | 56 | 35 | 59 | 25 | 28 | 11 | 55 | 38 | 2 | 23 | Mono |
| 383_1 | 52 | 53 | 30 | 58 | 24 | 27 | 12 | 64 | 50 | 1 | 10 | Mono |
| 303_1 | 52 | 54 | 20 | 47 | 20 | 20 | 8 | 53 | 36 | 2 | 11 | Poly |
| 365_1 | 36 | 37 | 22 | 34 | 25 | 19 | 10 | 39 | 25 | 4 | 17 | Poly |
| 377_1 | 55 | 58 | 32 | 48 | 24 | 32 | 10 | 54 | 39 | 2 | 10 | Poly |
| 397_1 | 61 | 58 | 50 | 51 | 25 | 41 | 16 | 64 | 38 | 2 | 16 | Poly |
| 398_1 | 60 | 60 | 34 | 52 | 25 | 34 | 12 | 57 | 43 | 1 | 12 | Poly |
| 58_1 | 62 | 64 | 53 | 67 | 26 | 27 | 10 | 61 | 56 | 2 | 9 | Poly |
| 3D7 | 60 | 60 | 16 | 52 | 13 | 13 | 3 | 54 | 36 | 2 | 17 | Reference |

**Table S7: Average percent (%) amino acid identity within PfEMP1 constitutive domains**

|  | | VAR2CSA | | | DBLpam1 | | DBLpam2 | | | CIDRpam | | DBLpam3 | | DBLεpam4 | | DBLεpam5 | | DBLε10 | | ID1-ID2a* | |
| --- | --- | --- | --- | --- | --- | --- | --- | --- | --- | --- | --- | --- | --- | --- | --- | --- | --- | --- | --- | --- | --- |
| Mali | | 79 | | | 78 | | 79 | | | 80 | | 89 | | 92 | | 89 | | 62 | | 74 | |
| Rask *et al.* 2010 | | NA | | | 78 | | 77 | | | 75 | | 87 | | 90 | | 80 | | 58 | | NA | |
| Global | | 77 | | | 77 | | 78 | | | 78 | | 88 | | 91 | | 87 | | 60 | |  | |
|  | **DBLα** | | | **DBLβ** | | **DBLδ** | | **DBLε** | | | **DBLγ** | | **DBLζ** | | **CIDRα** | | **CIDRβ** | | **CIDRδ** | | **CIDRγ** |
| Mali | 42 | | | 43 | | 33 | | 31 | | | 37 | | 40 | | 30 | | 37 | | 55 | | 36 |
| Rask *et al.* 2010 | 42 | | | 45 | | 38 | | 31 | | | 37 | | 41 | | 33 | | 41 | | 60 | | 37 |
|  | | |  | | |  | | |  |  |  |  |  |  |  |  |  |  |  |  |  |
|  | | | **VAR1** | | | **VAR3** | | |  |  |  |  |  |  |  |  |  |  |  |  |  |
| Mali | | | 67 | | | 87 | | |  |  |  |  |  |  |  |  |  |  |  |  |  |
| Rask *et al.* 2010 | | | 69 | | | 84 | | |  |  |  |  |  |  |  |  |  |  |  |  |  |

*Minimal CSA-binding binding region; focus of the current VAR2CSA-based vaccine.
