# Supplementary material for: New var reconstruction algorithm exposes high var sequence diversity in a single geographic location in Mali
Source: Genome Med. 2017 Mar 28;9:30. doi: 10.1186/s13073-017-0422-4 (PMC5368897; doi:10.1186/s13073-017-0422-4)
Supplement: Supplementary file 2 — Contig pile-ups of the genome of 12 P. falciparum isolates from Mali aligned against the reference 3D7 genome. The genome assembly of each of the 12 isolates, represented by its constituent contigs (blue lines in between blue circles) is aligned against each of the 14 nuclear chromosomes of the reference P. falciparum 3D7 strain (black). The location of var gene sequences is shown (red) in both the 3D7 genome and the contigs aligned to it. (PDF 1608 kb) [file 13073_2017_422_MOESM2_ESM.pdf]

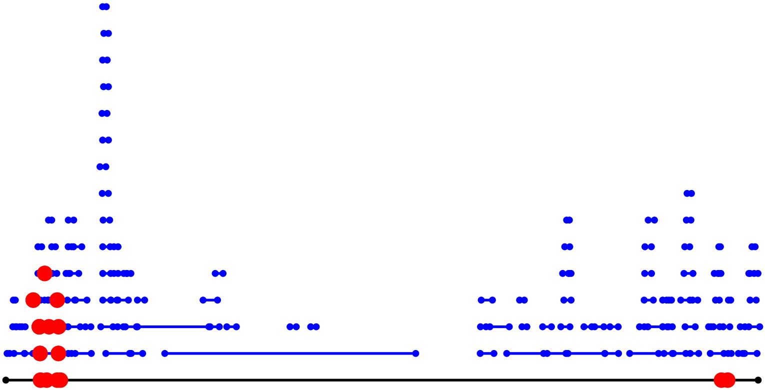

58_1 Chrm 1

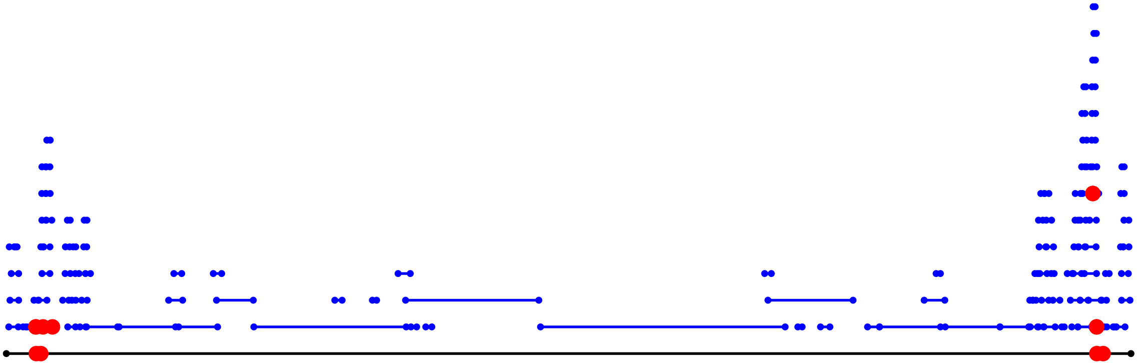

58_1 Chrm 2

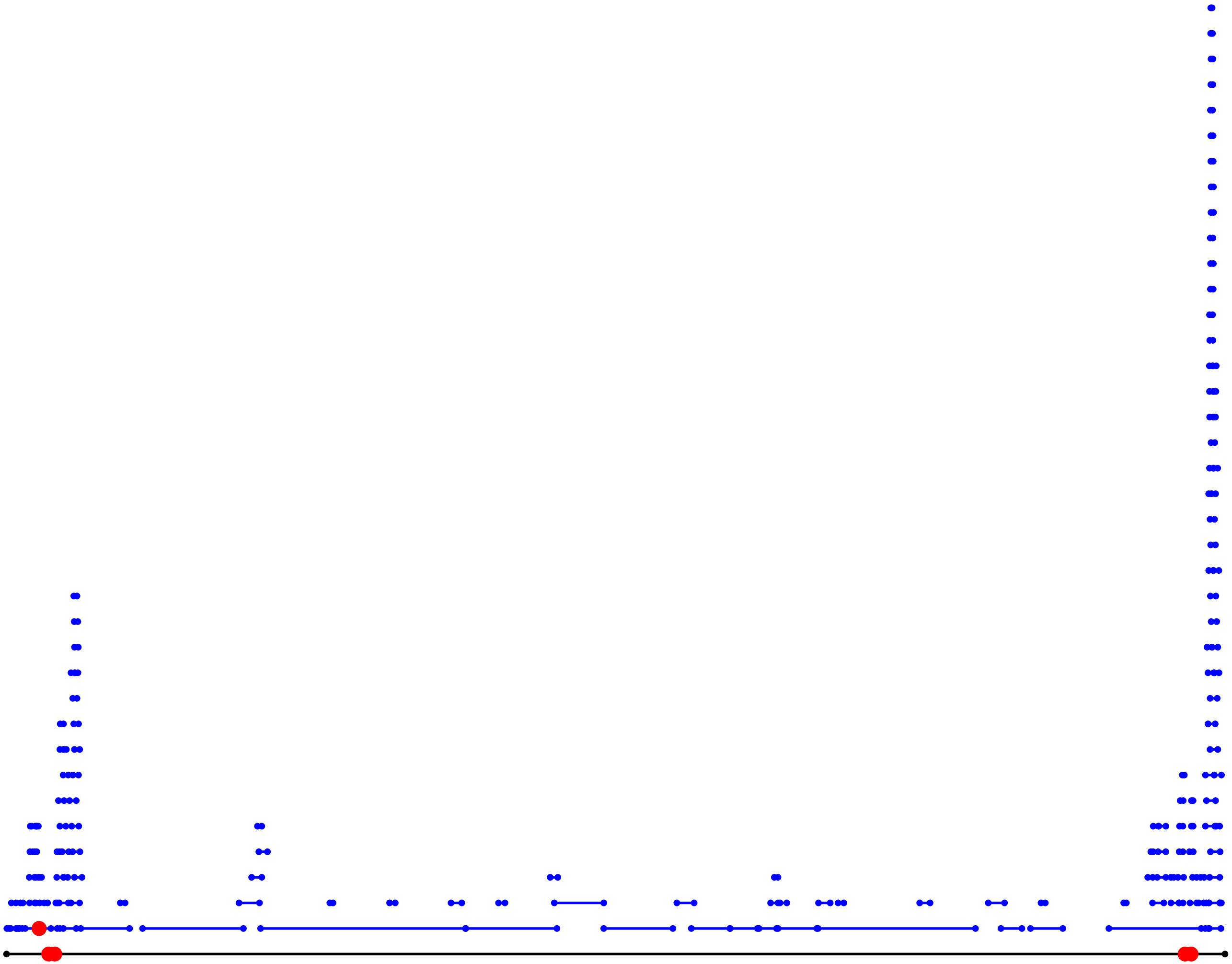

58_1 Chrm 3

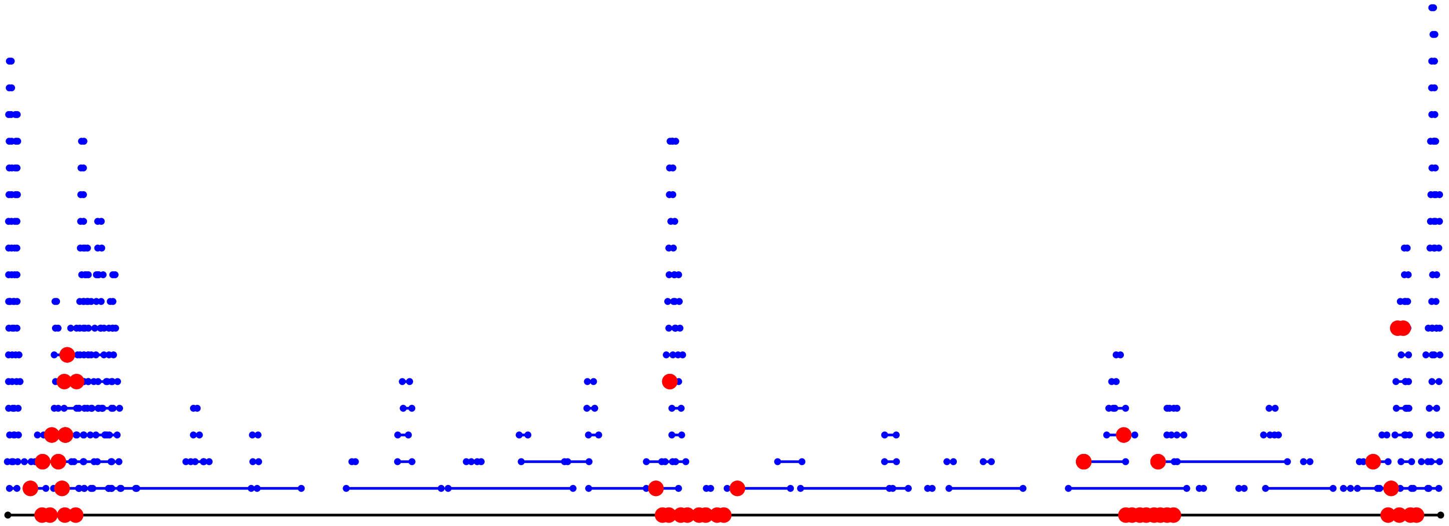

58_1 Chrm 4

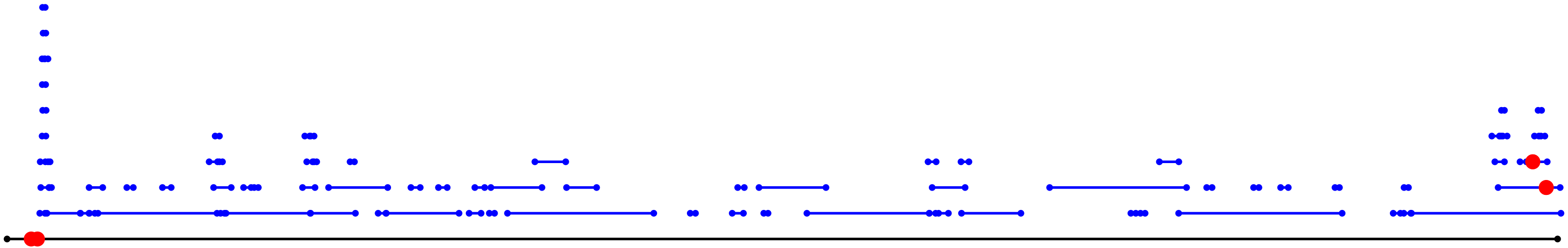

58_1 Chrm 10

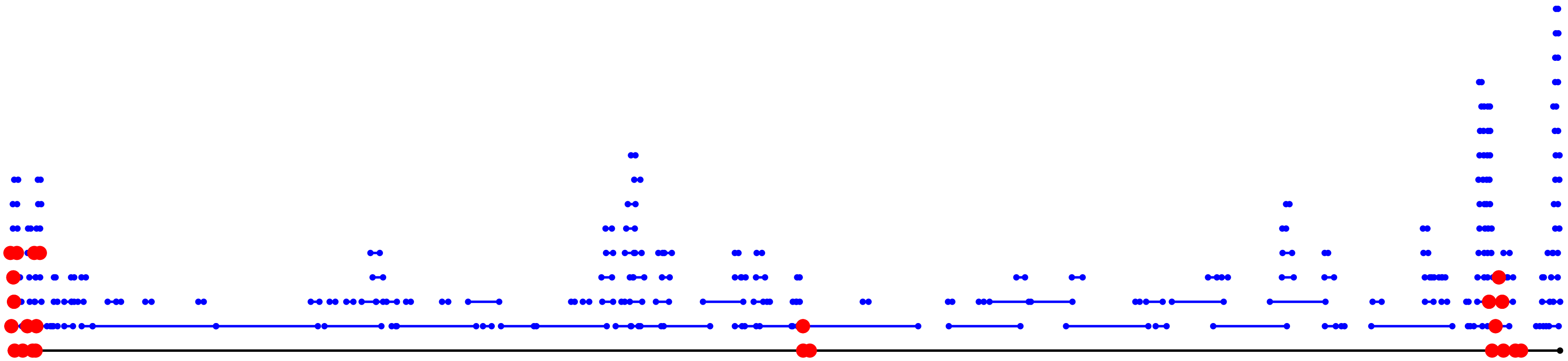

58_1 Chrm 6

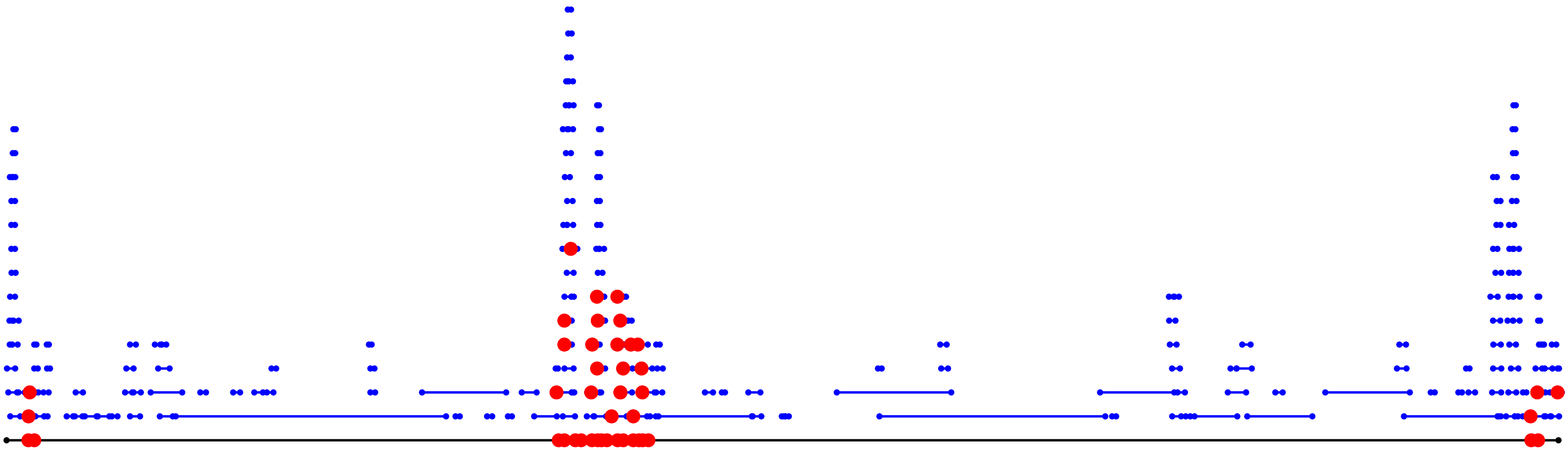

58_1 Chrm 7

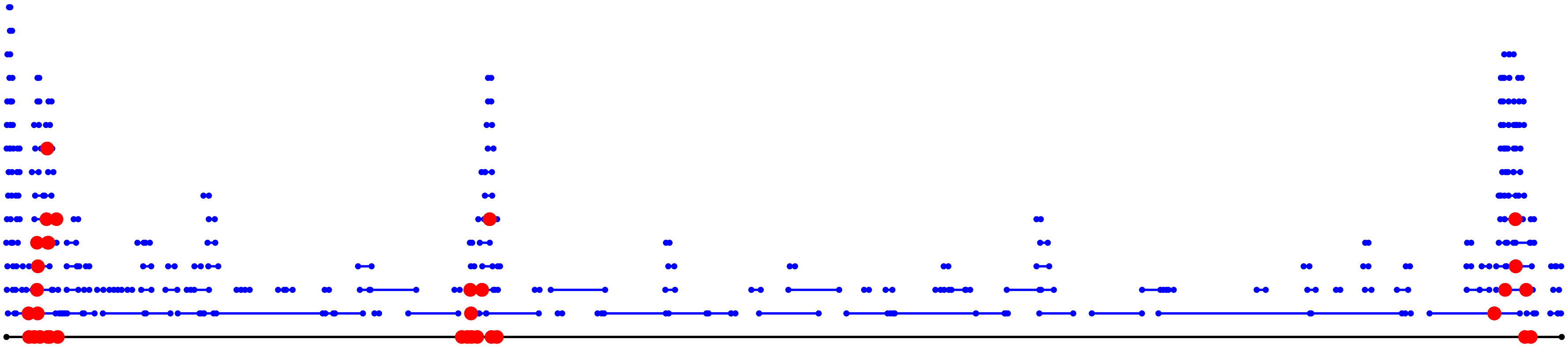

58_1 Chrm 8

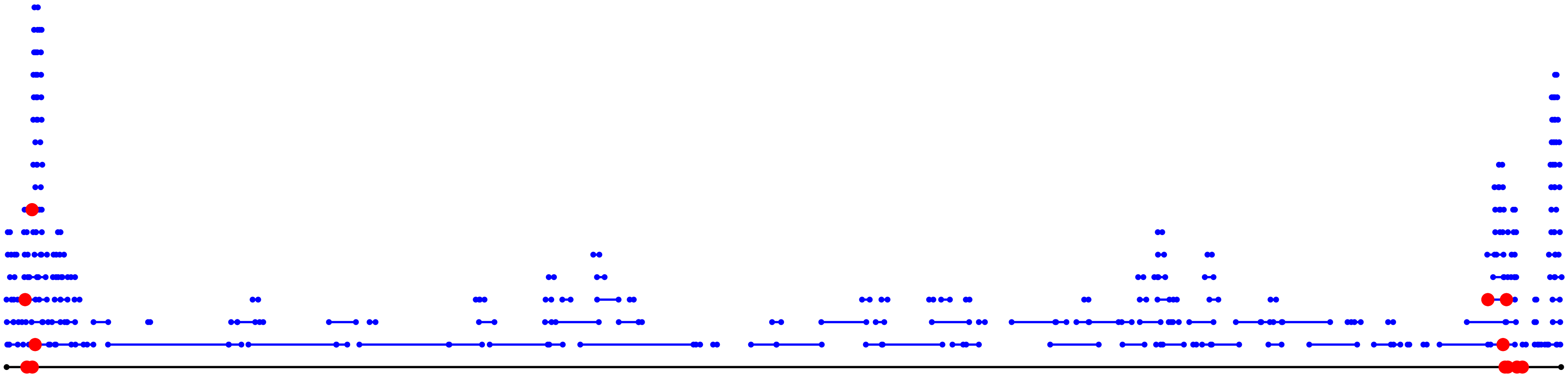

58_1 Chrm 9

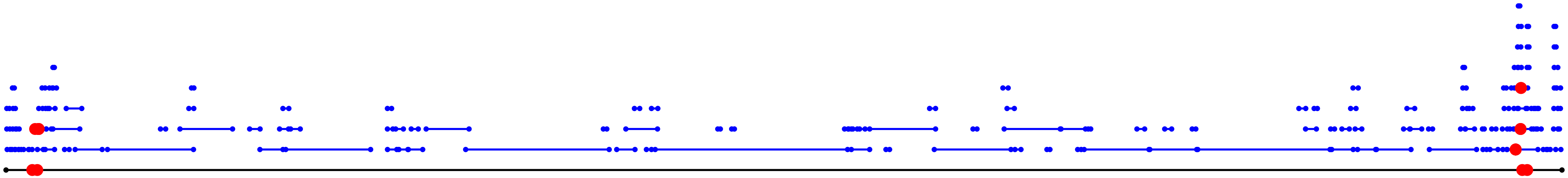

58_1 Chrm 10

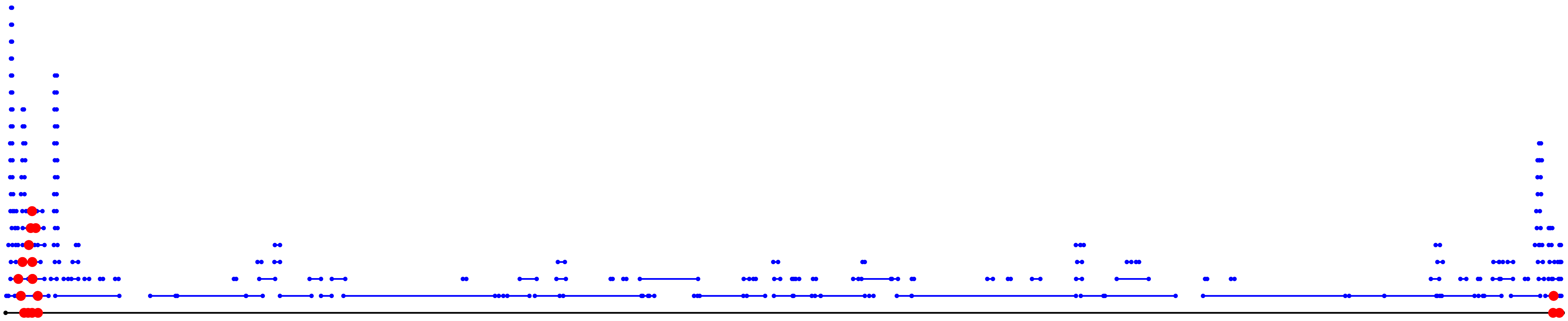

58_1 Chrm 11

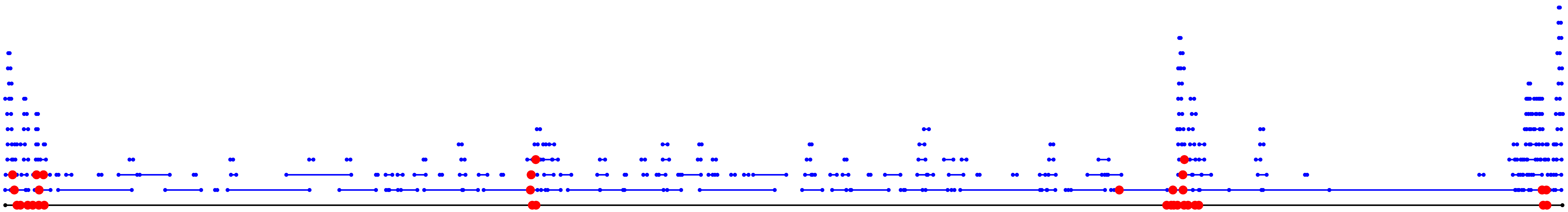

58_1 Chrm 12

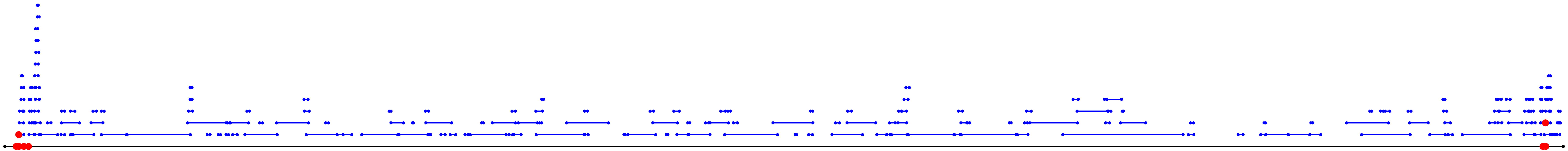

58_1 Chrm 13

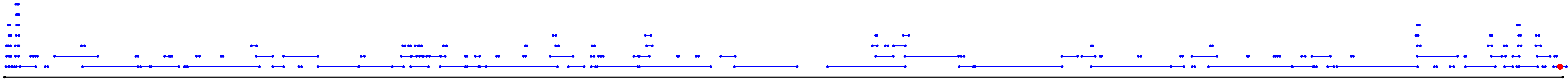

58_1 Chrm 14

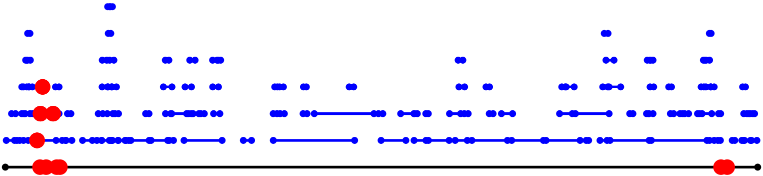

303_1 Chrm 1

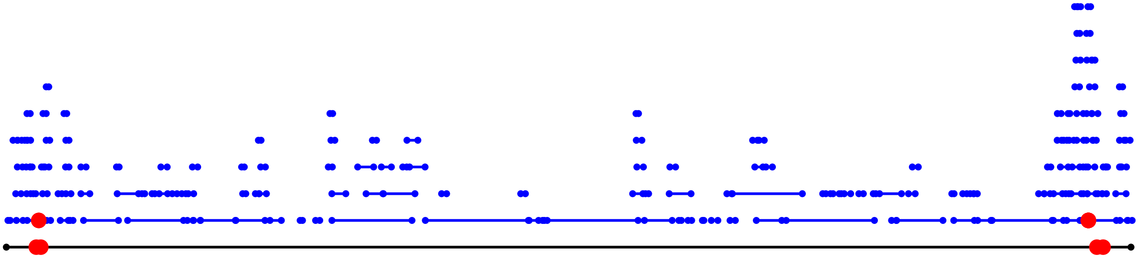

303_1 Chrm 2

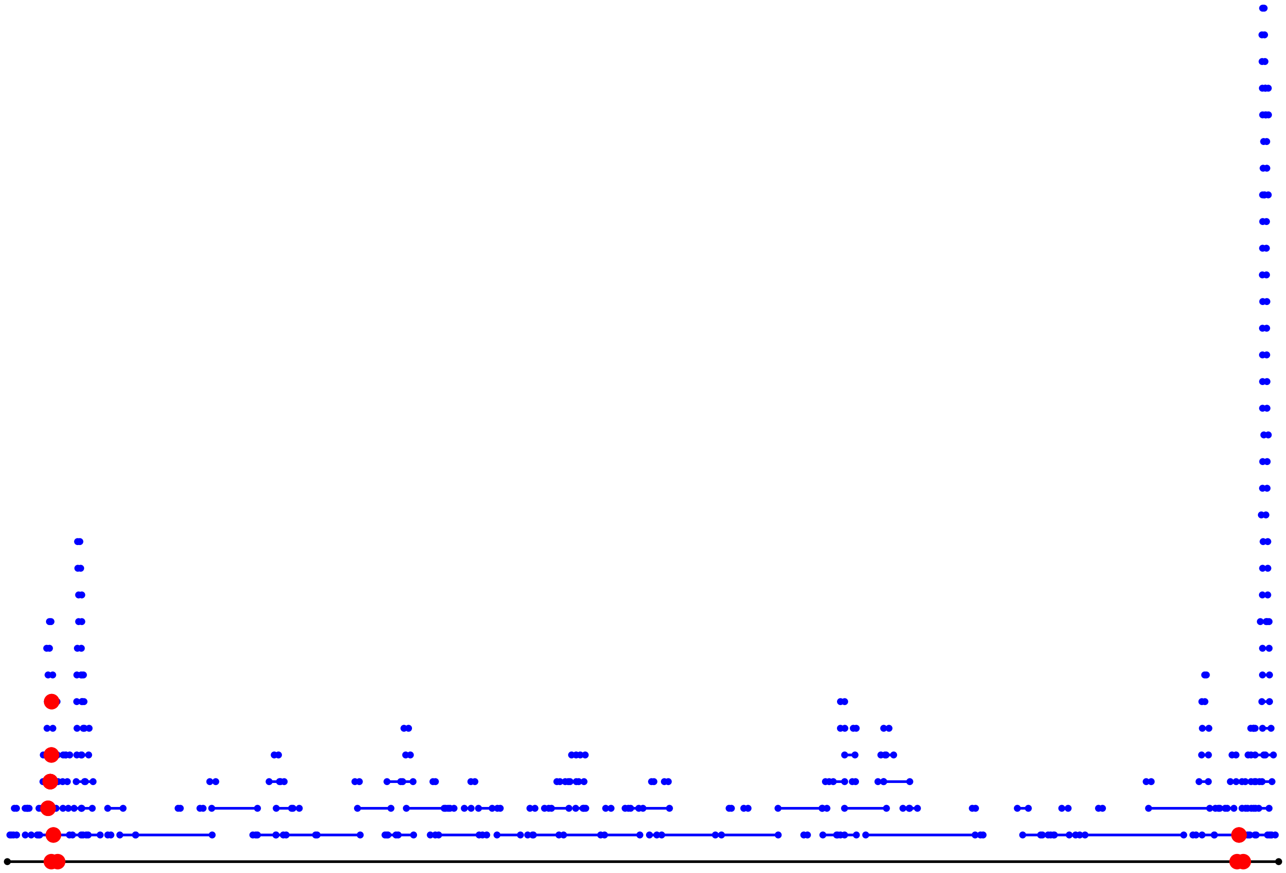

303_1 Chrm 3

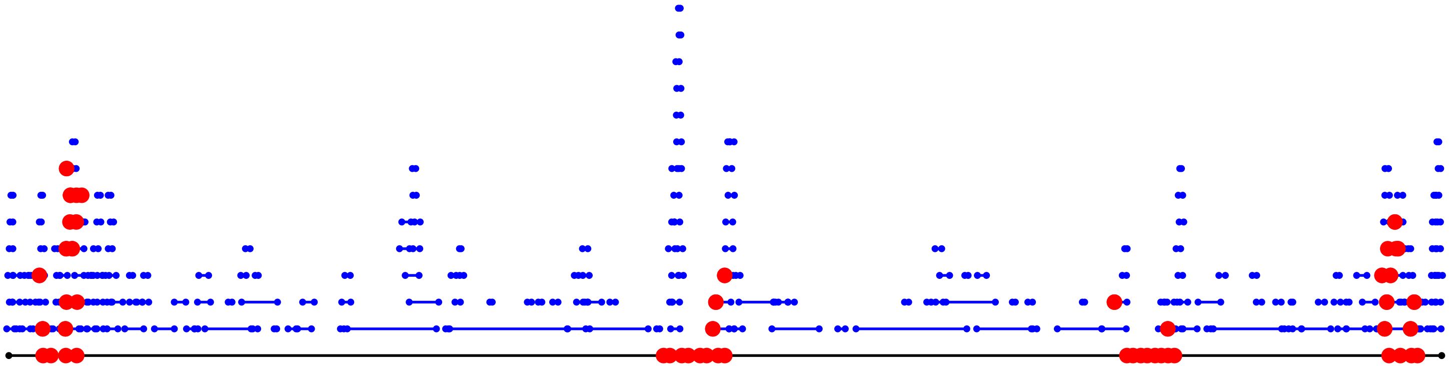

303_1 Chrm 4

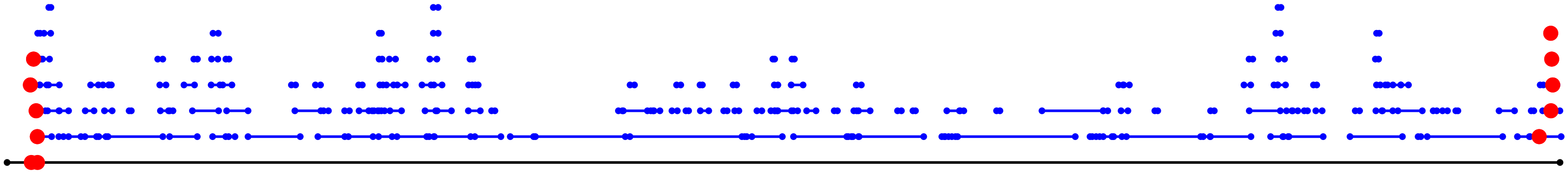

303_1 Chrm 5

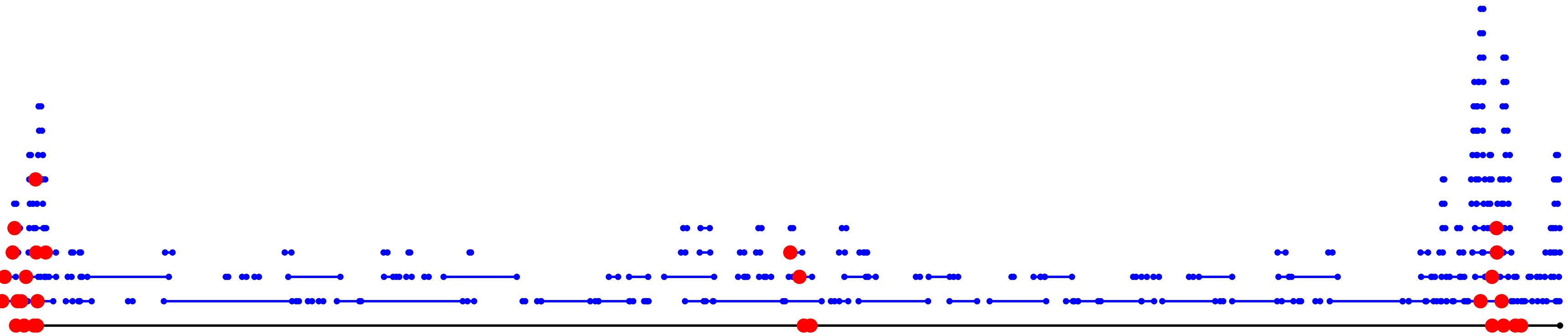

303_1 Chrm 6

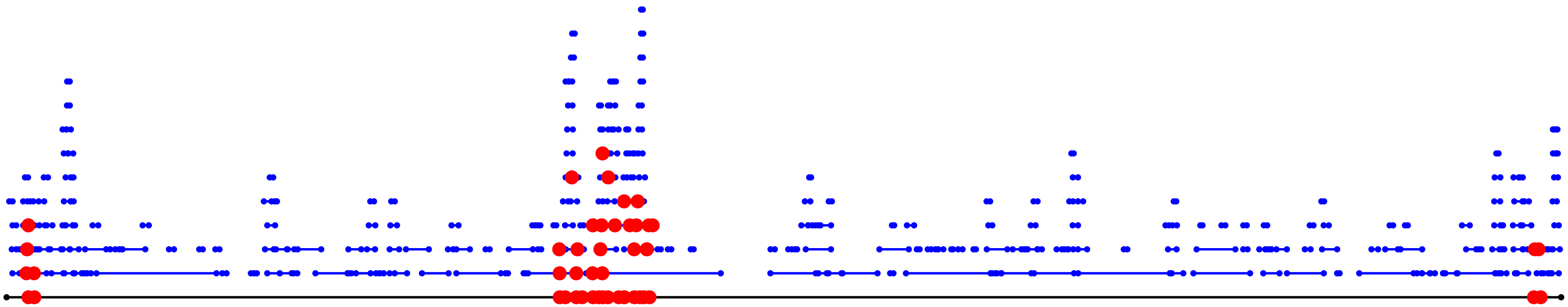

303_1 Chrm 7

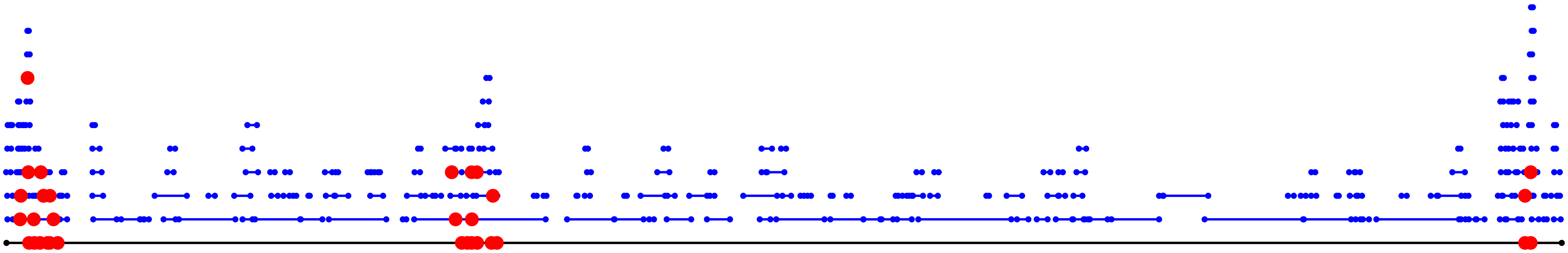

303_1 Chrm 8

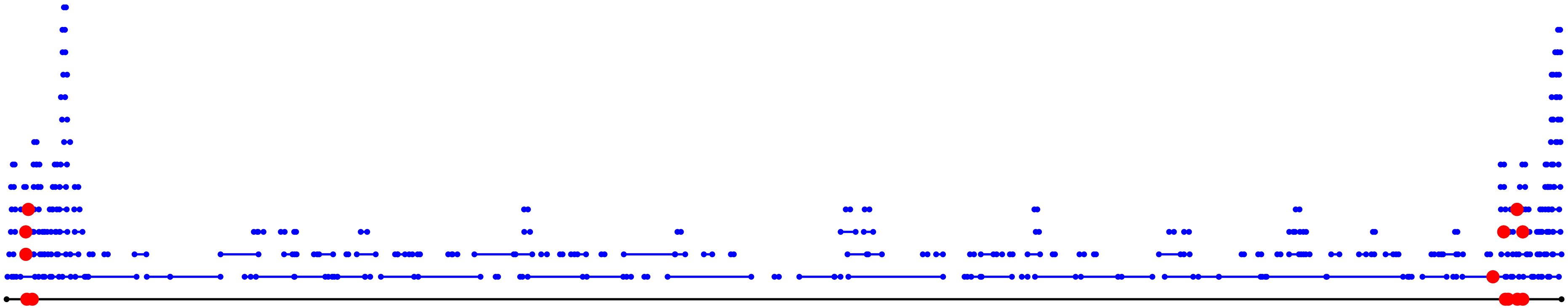

303_1 Chrm 9

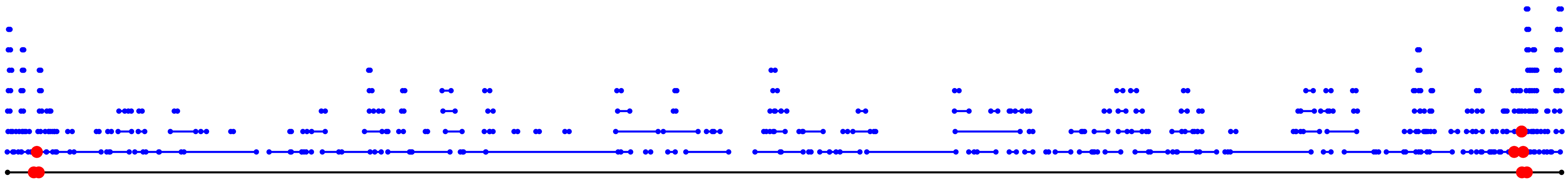
303_1 Chrm 10

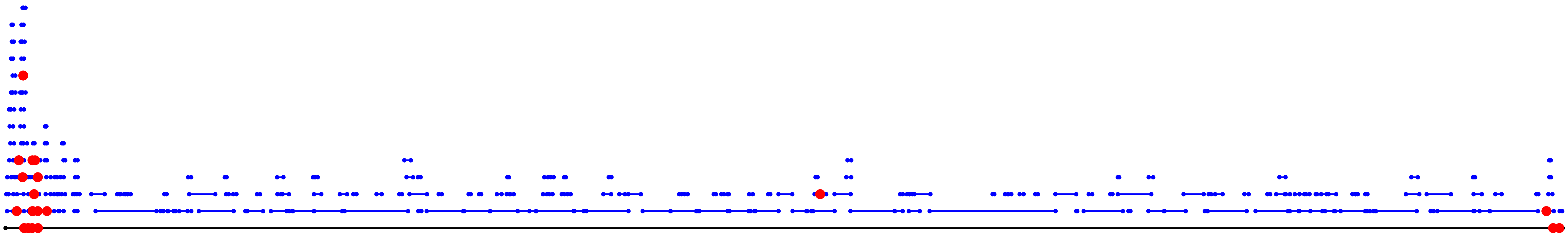

303_1 Chrm 11

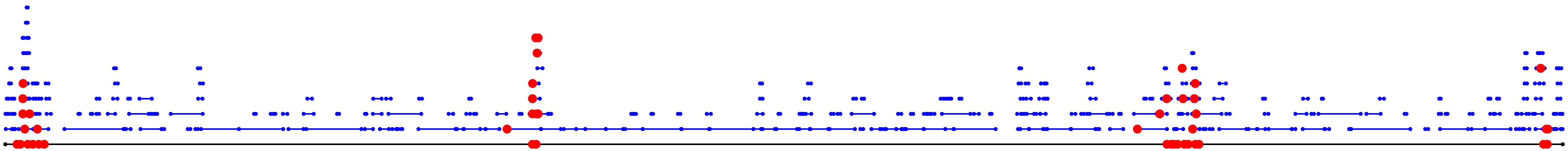

303_1 Chrm 12

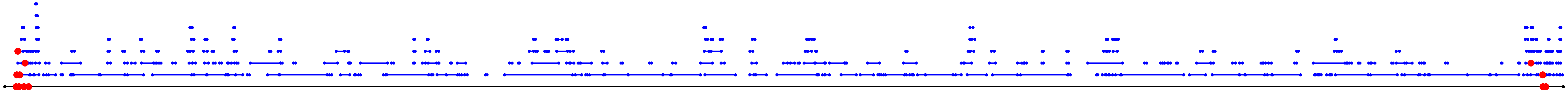

303_1 Chrm 13

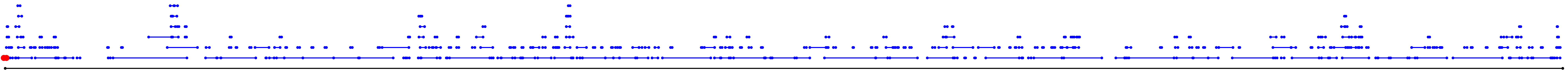

303_1 Chrm 14

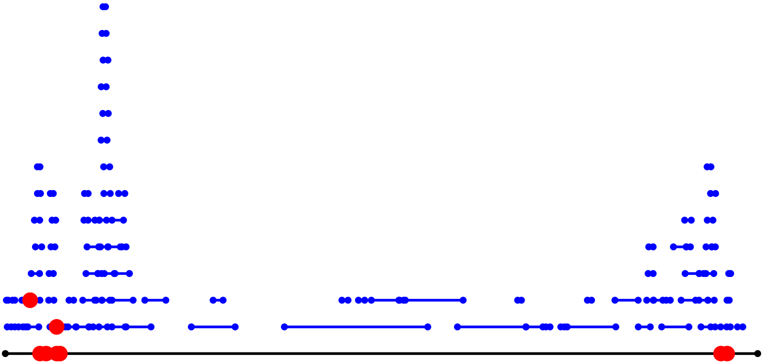

309_1 Chrm 1

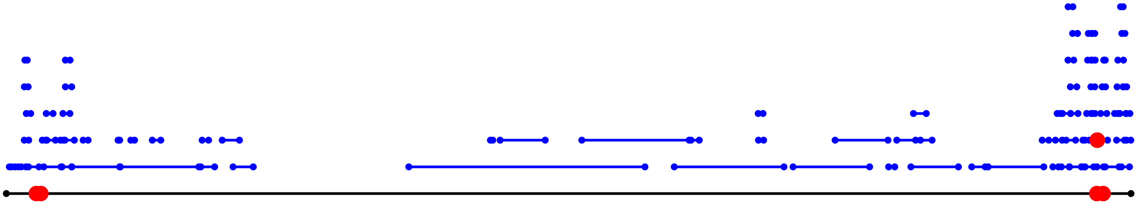

309_1 Chrm 2

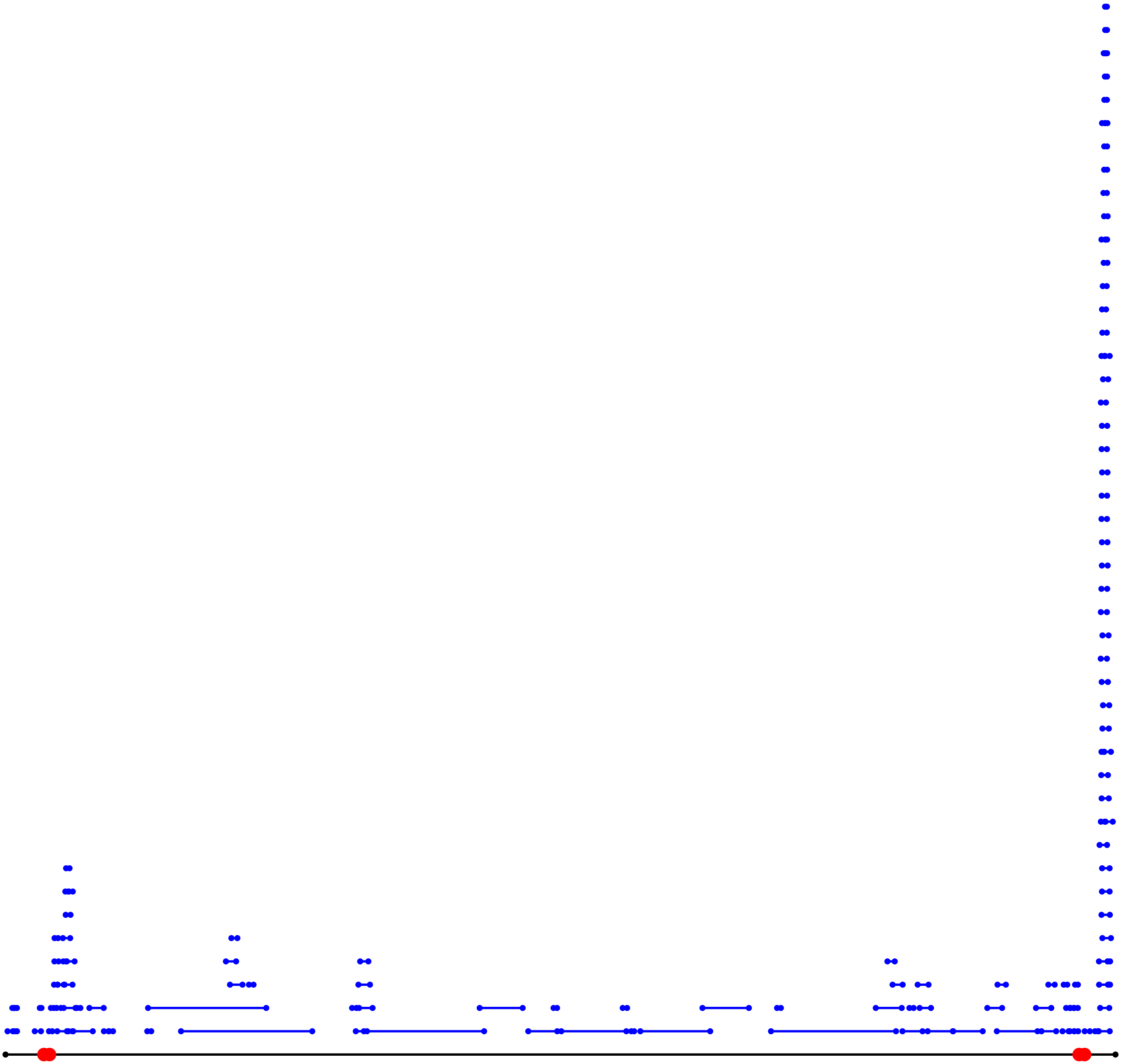

309_1 Chrm 3

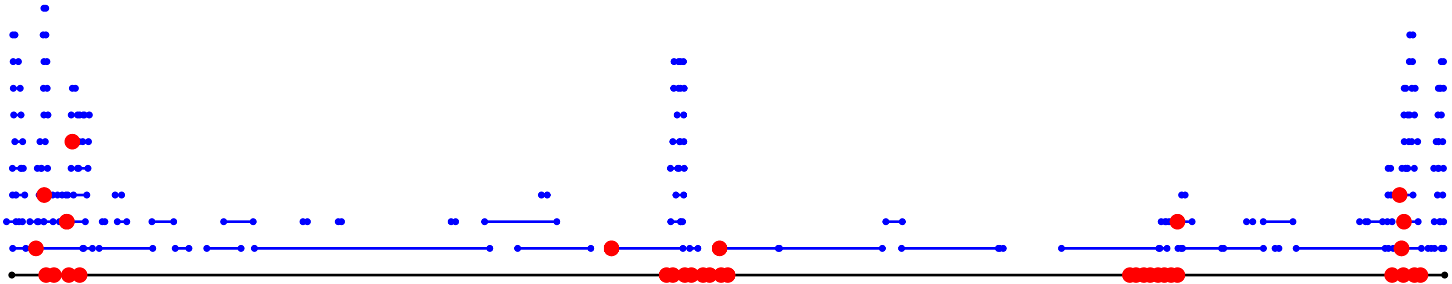

309_1 Chrm 4

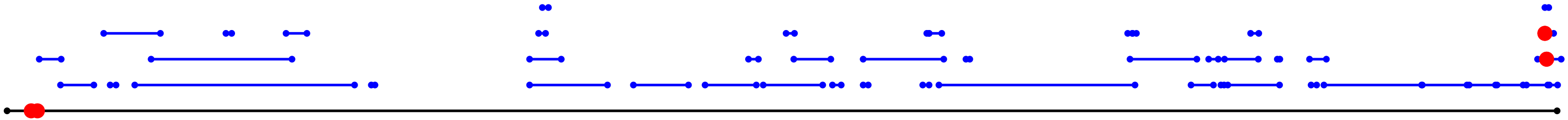

309_1 Chrm 5

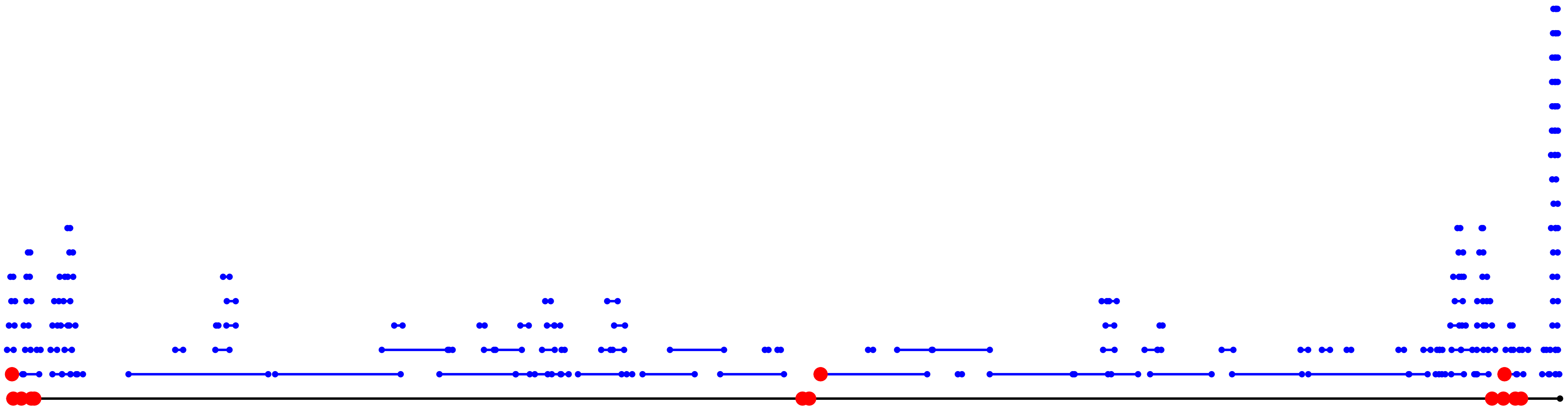

309_1 Chrm 6

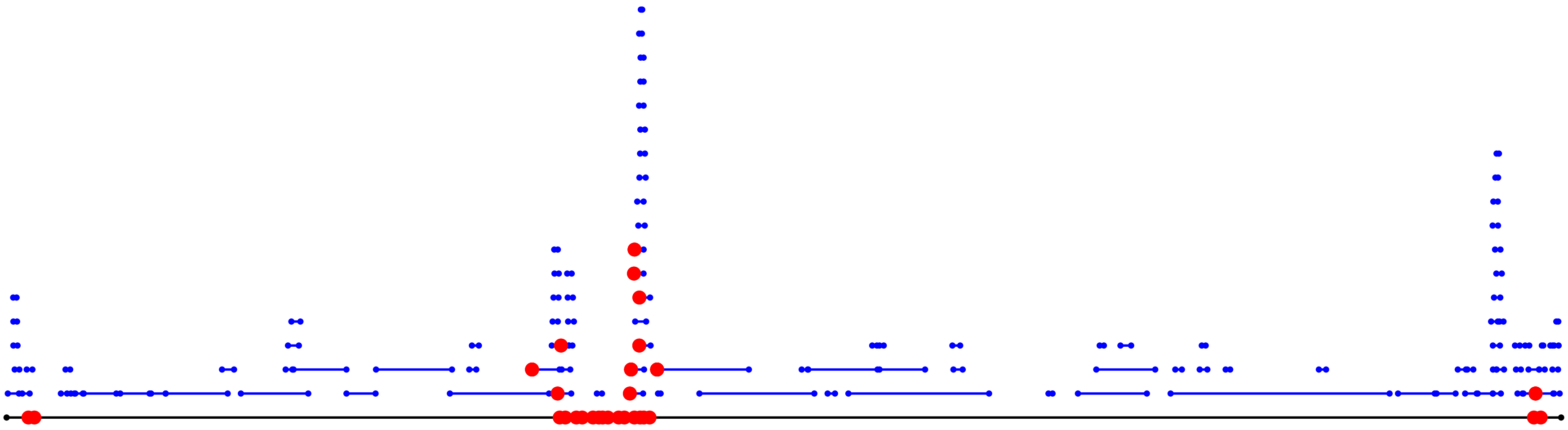

309_1 Chrm 7

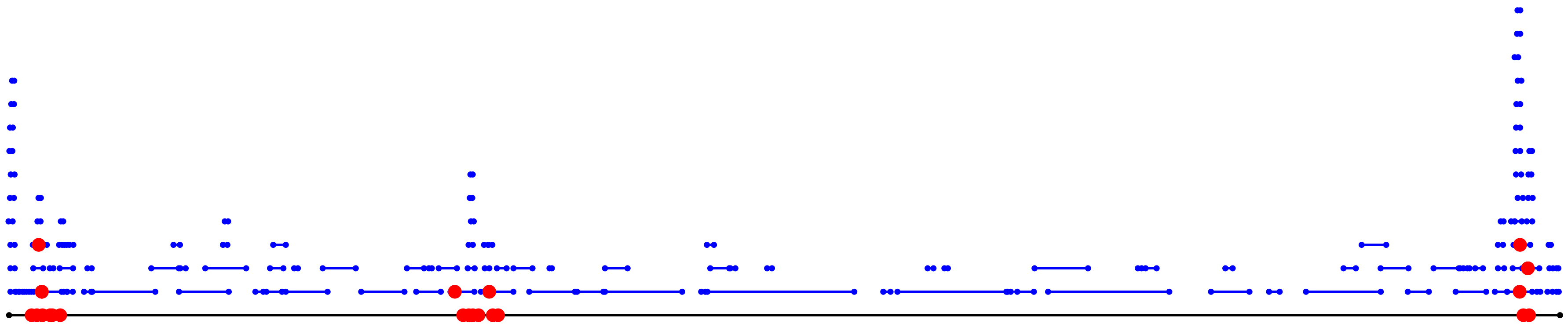

309_1 Chrm 8

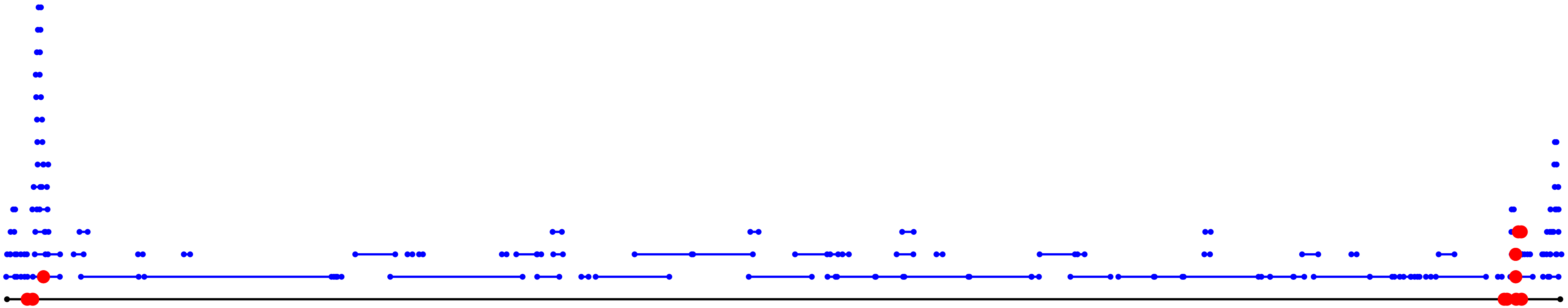

309_1 Chrm 9

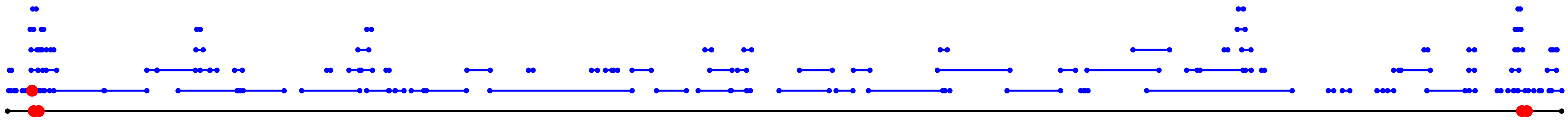

309_1 Chrm 10

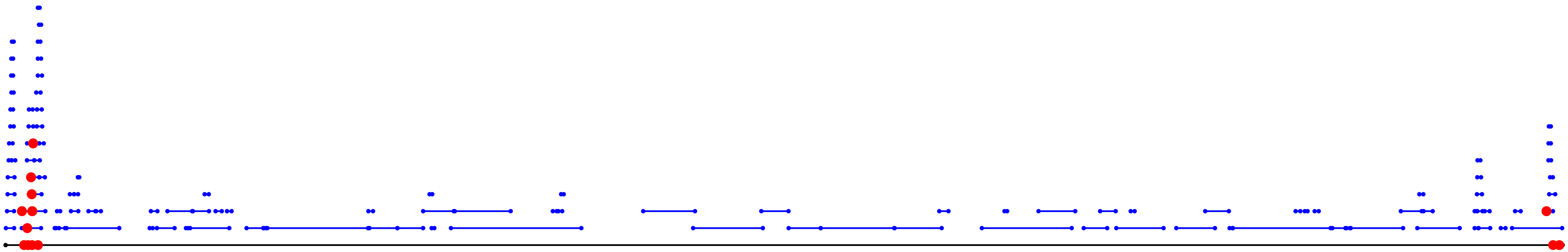

309_1 Chrm 11

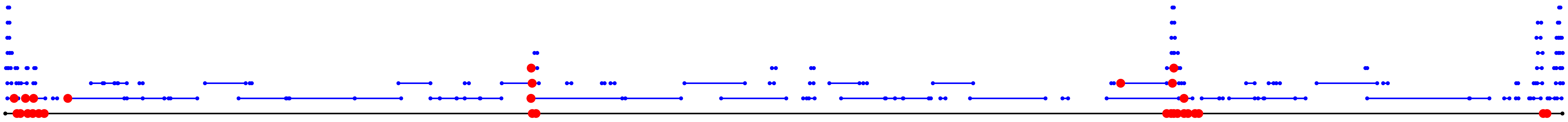

309_1 Chrm 12

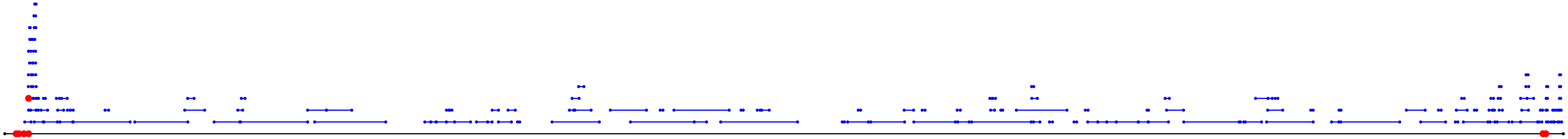

309_1 Chrm 13

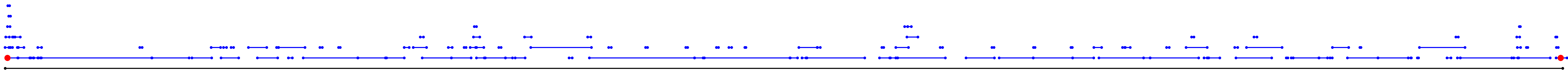

309_1 Chrm 14

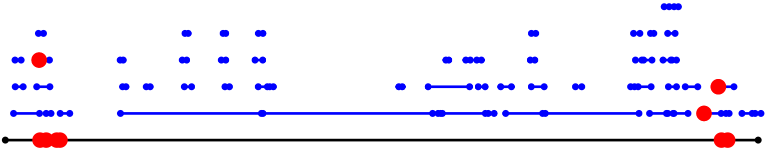

318_1 Chrm 1

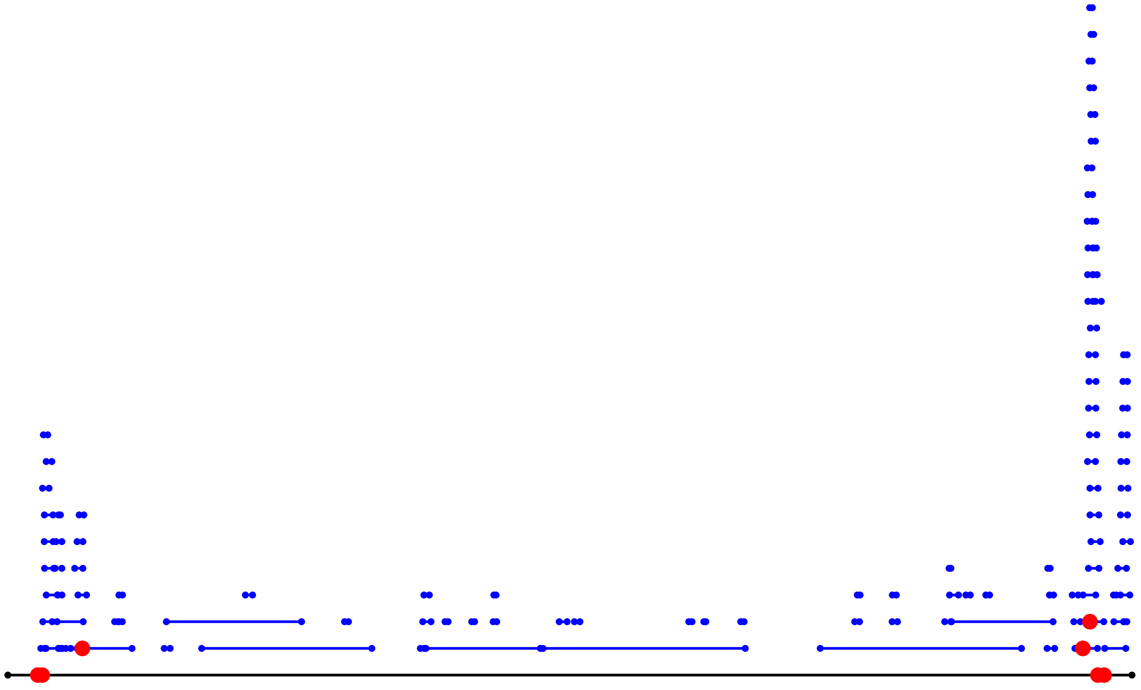

318_1 Chrm 2

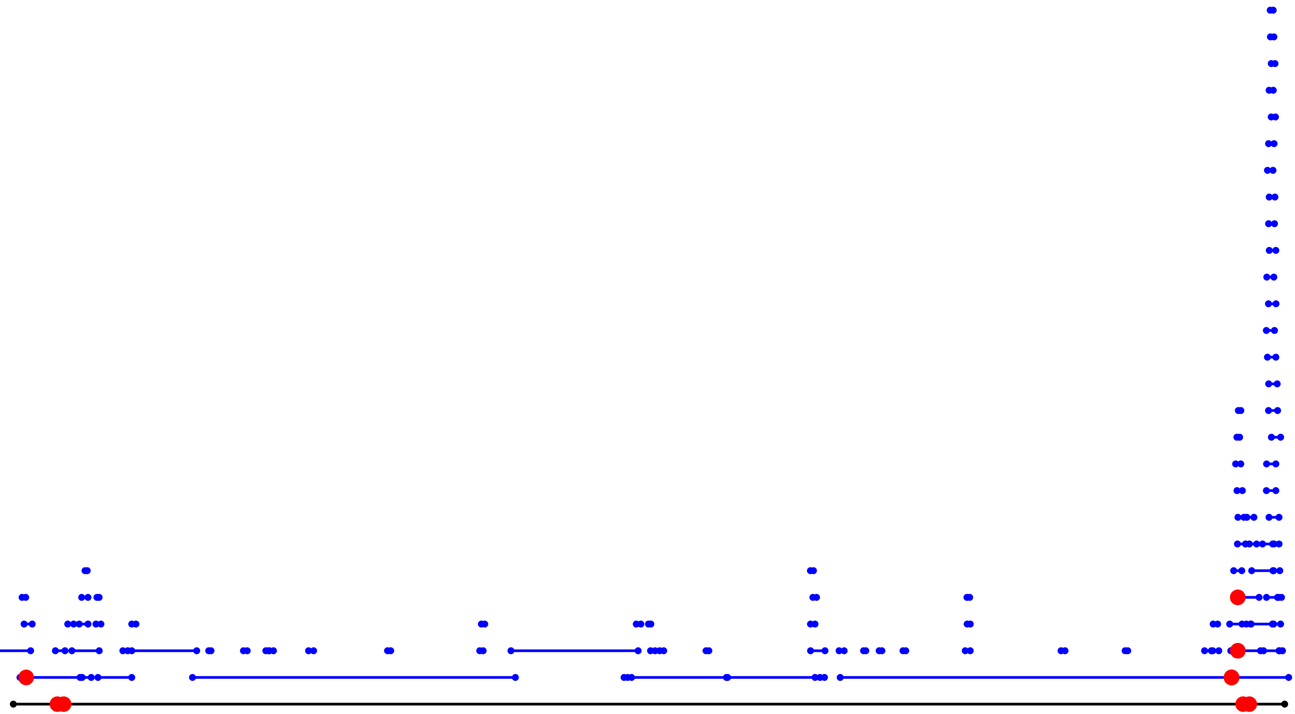

318_1 Chrm 3

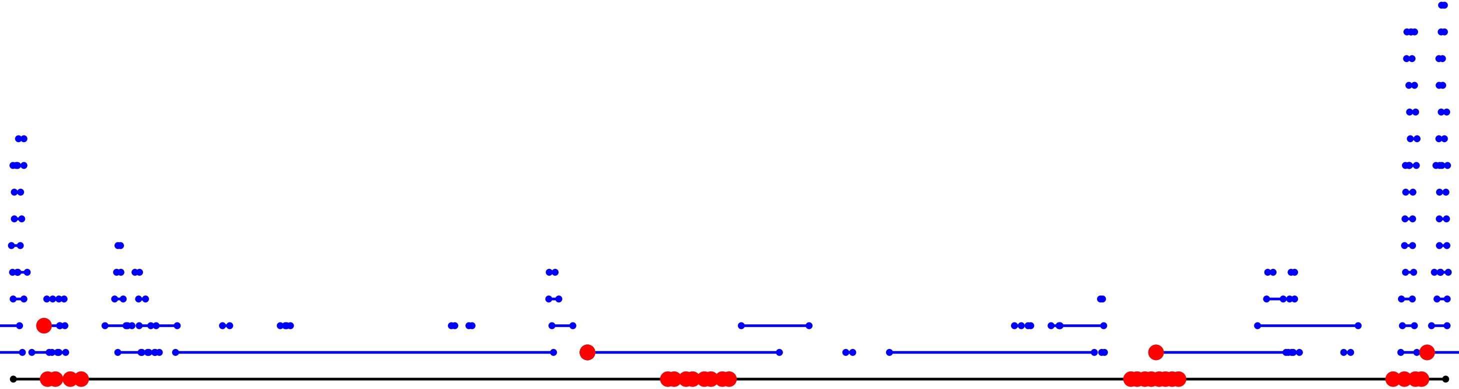

318_1 Chrm 4

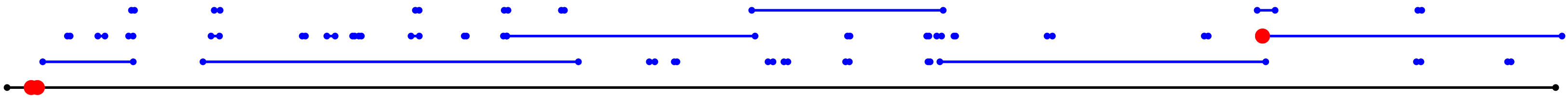

318_1 Chrm 5

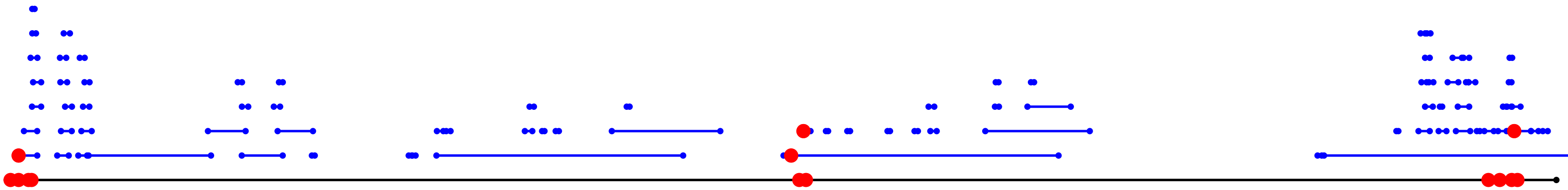

318_1 Chrm 6

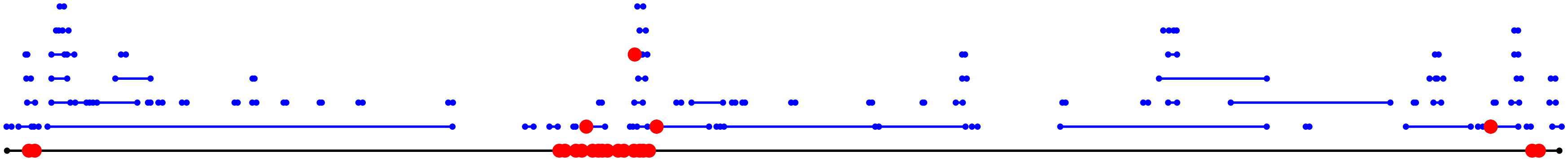

318_1 Chrm 7

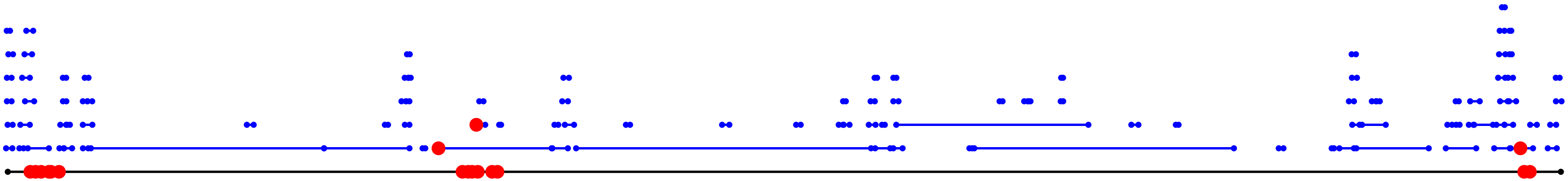

318_1 Chrm 8

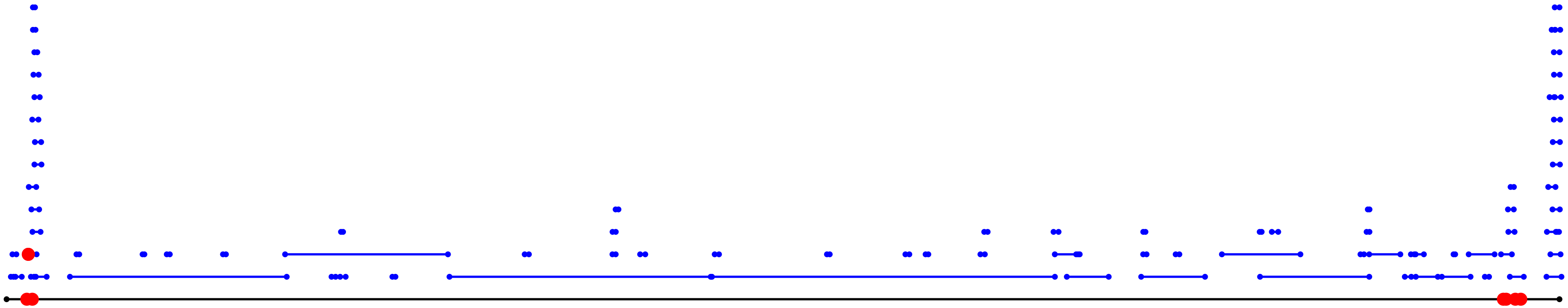

318_1 Chrm 9

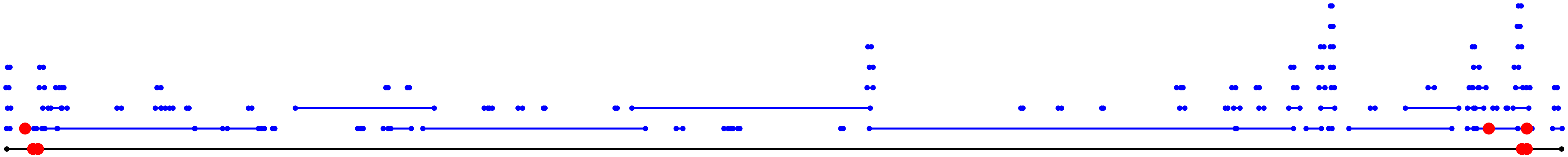

318_1 Chrm 10

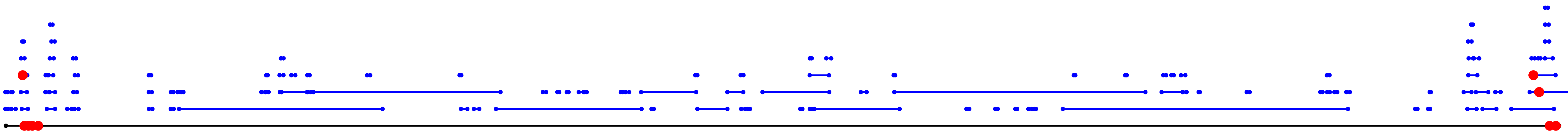

318_1 Chrm 11

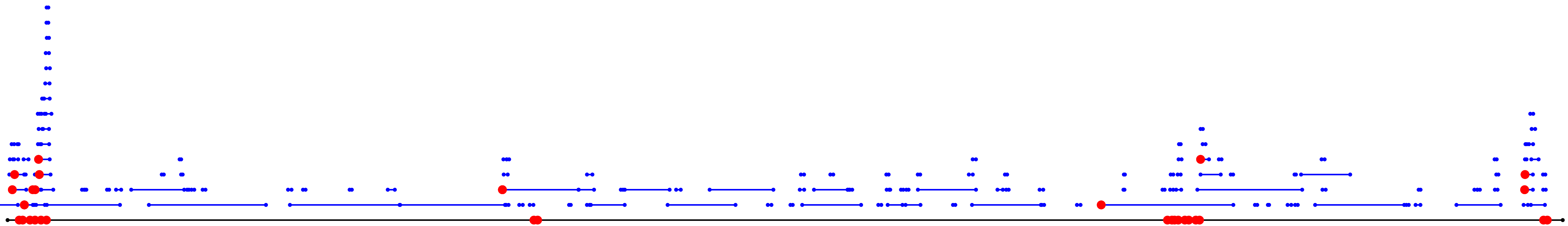

318_1 Chrm 12

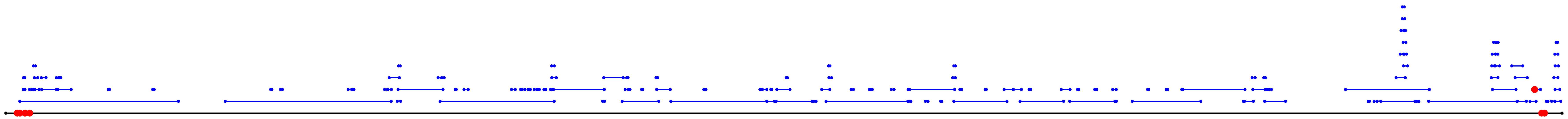

318_1 Chrm 13

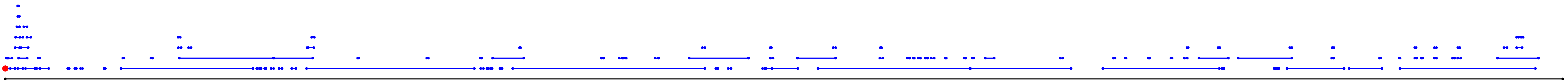

318_1 Chrm 14

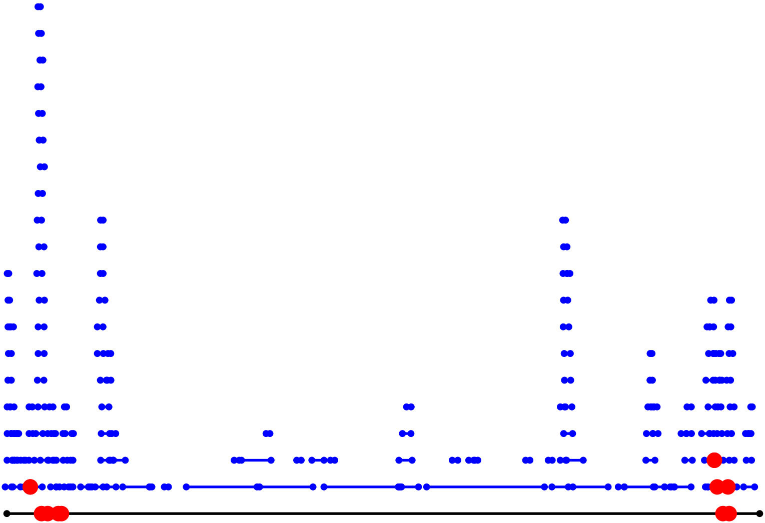

326_1 Chrm 1

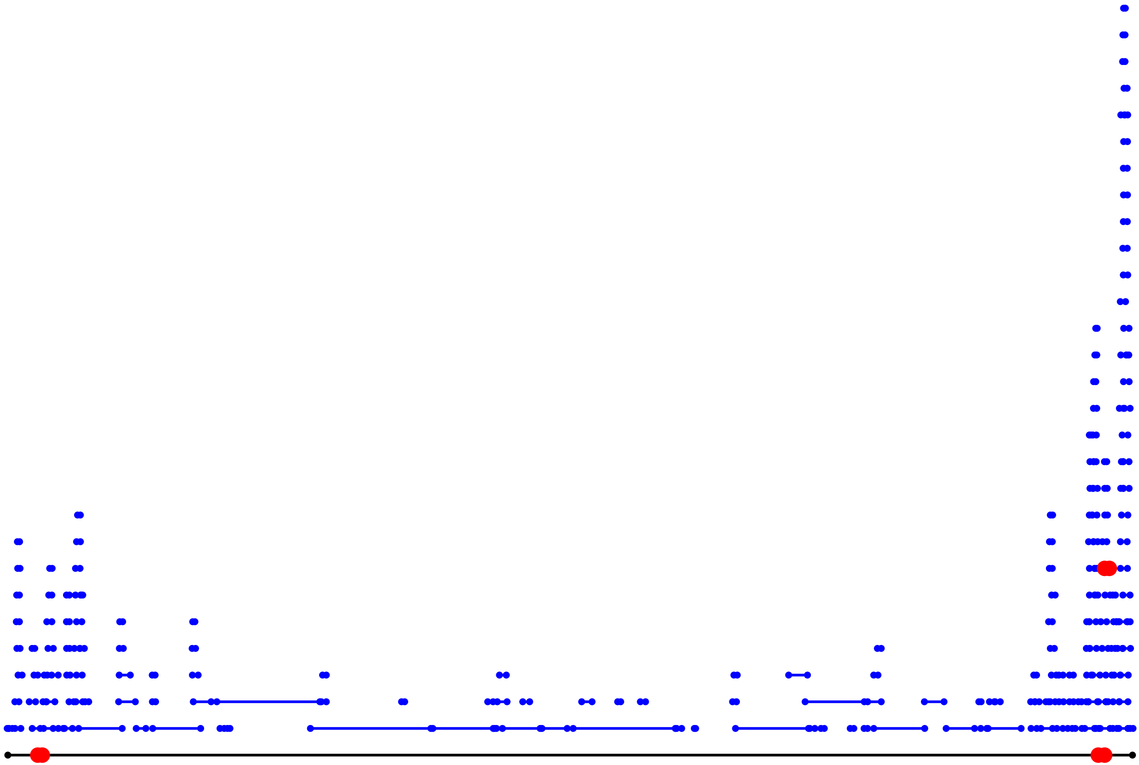

326_1 Chrm 2

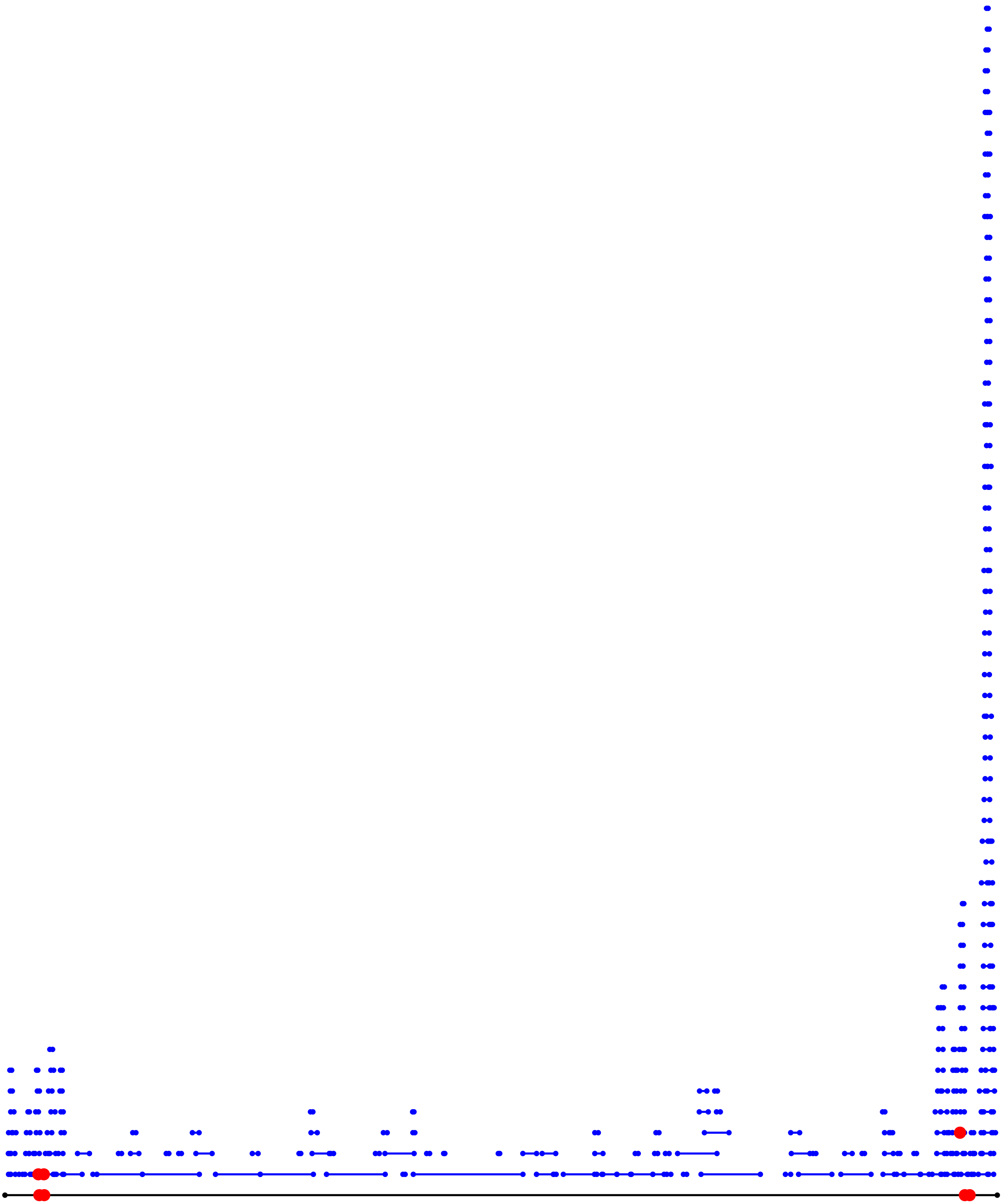

326_1 Chrm 3

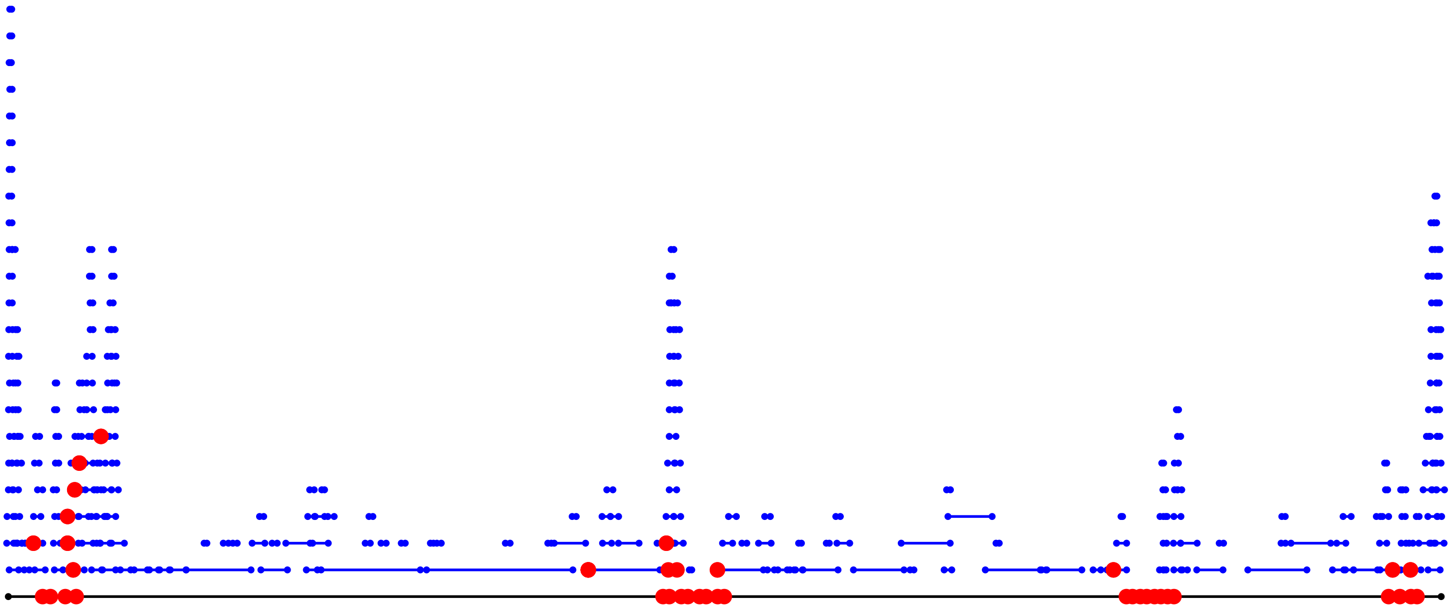

326_1 Chrm 4

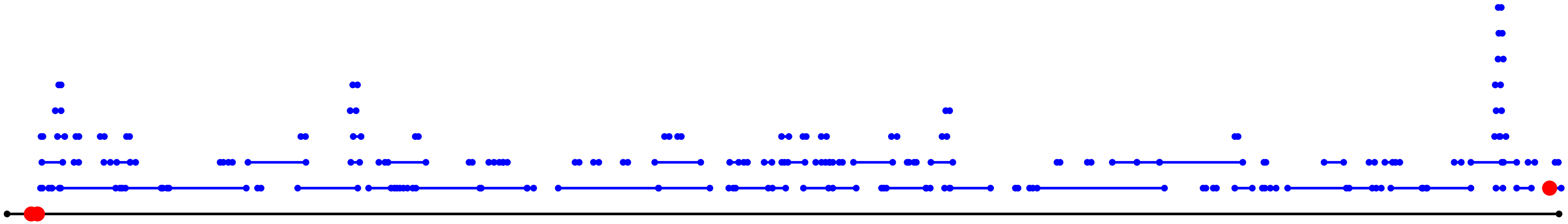

326_1 Chrm 5

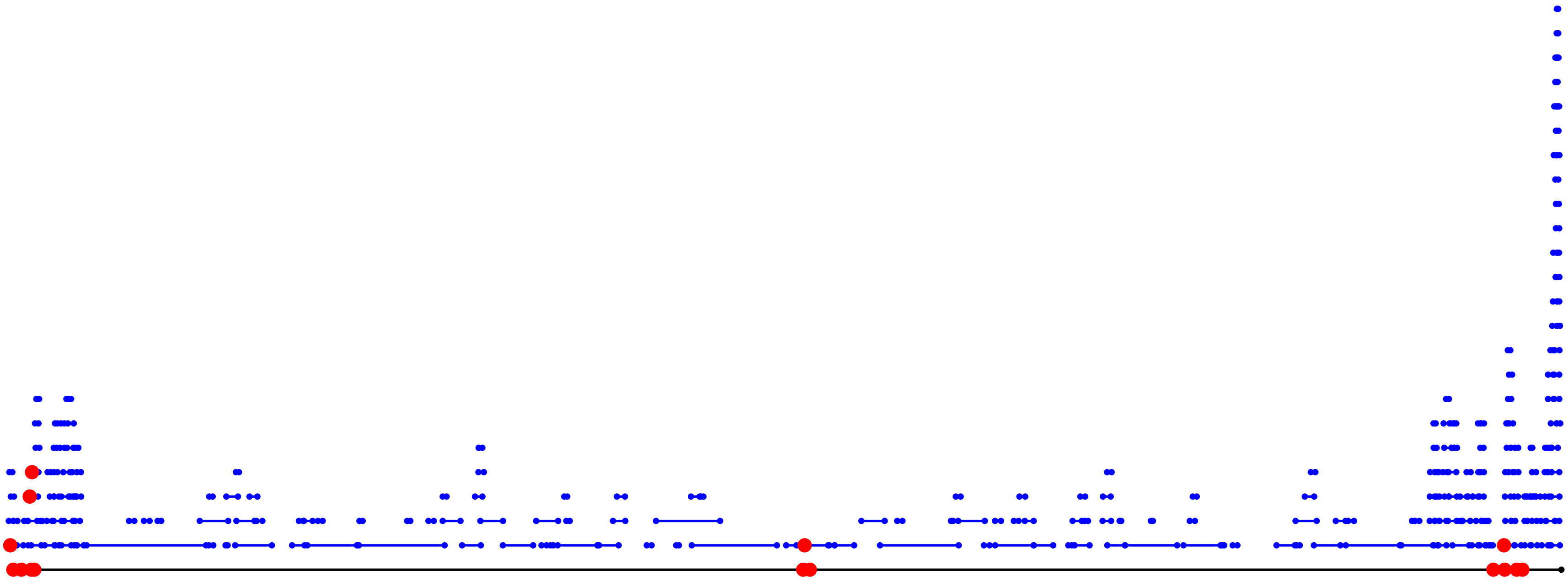

326_1 Chrm 6

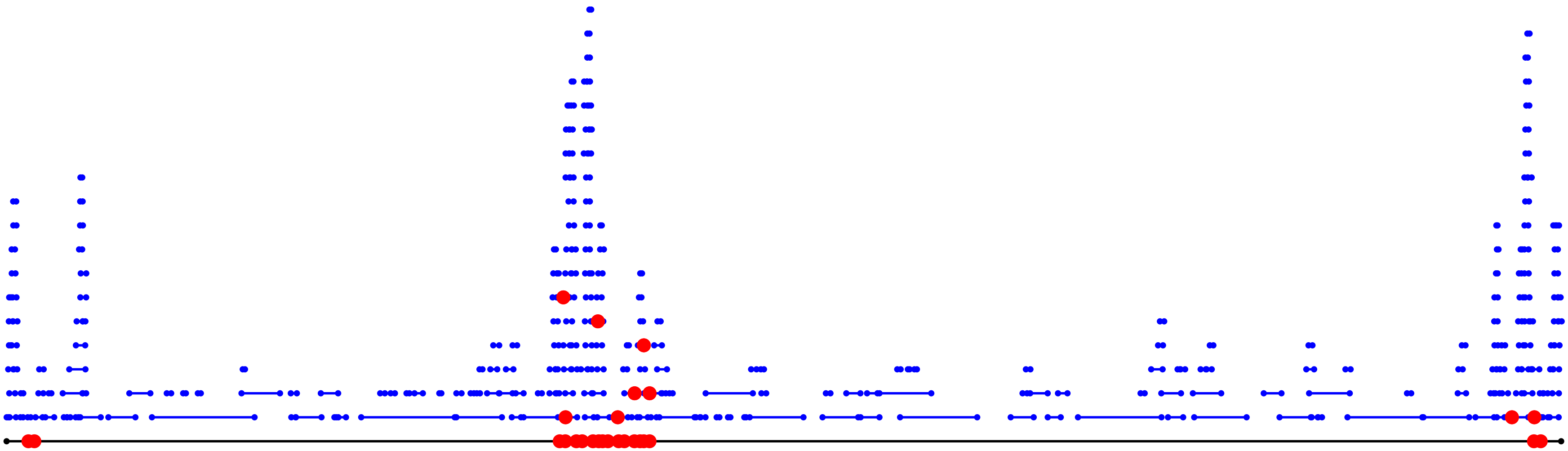

326_1 Chrm 7

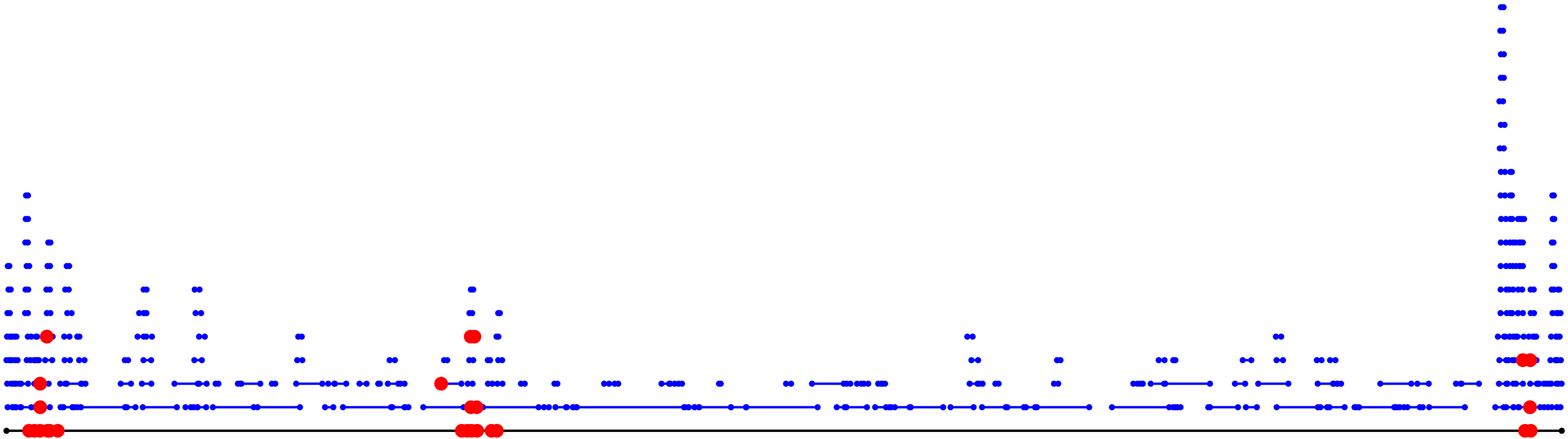

326_1 Chrm 8

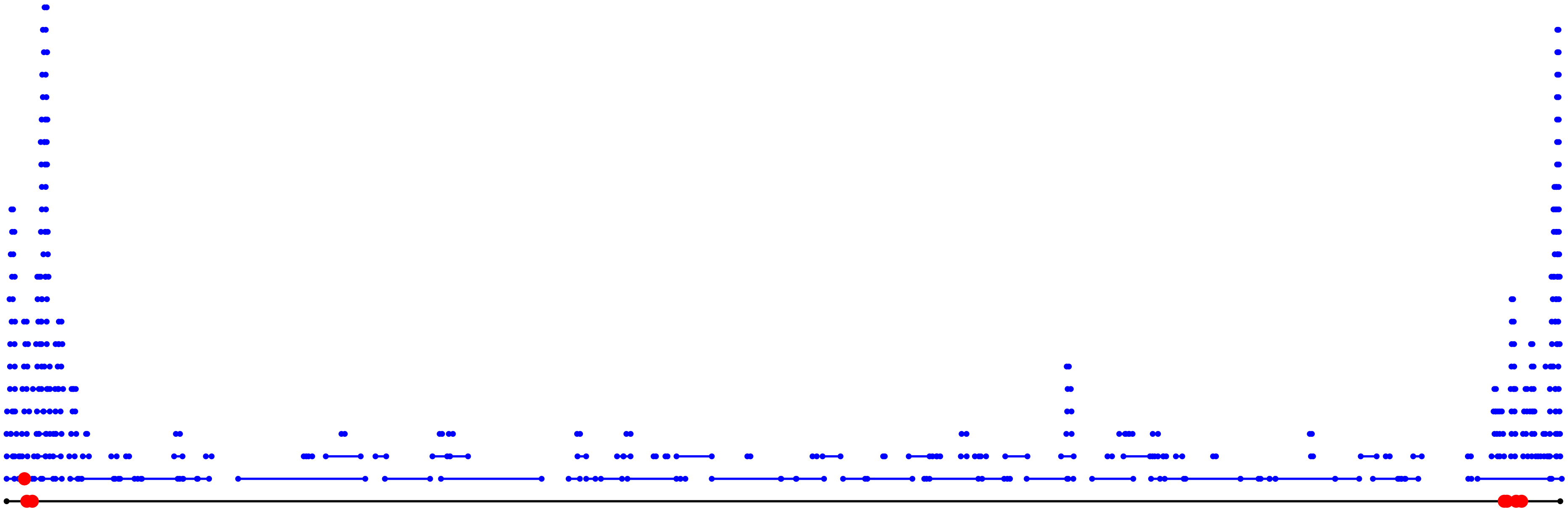

326_1 Chrm 9

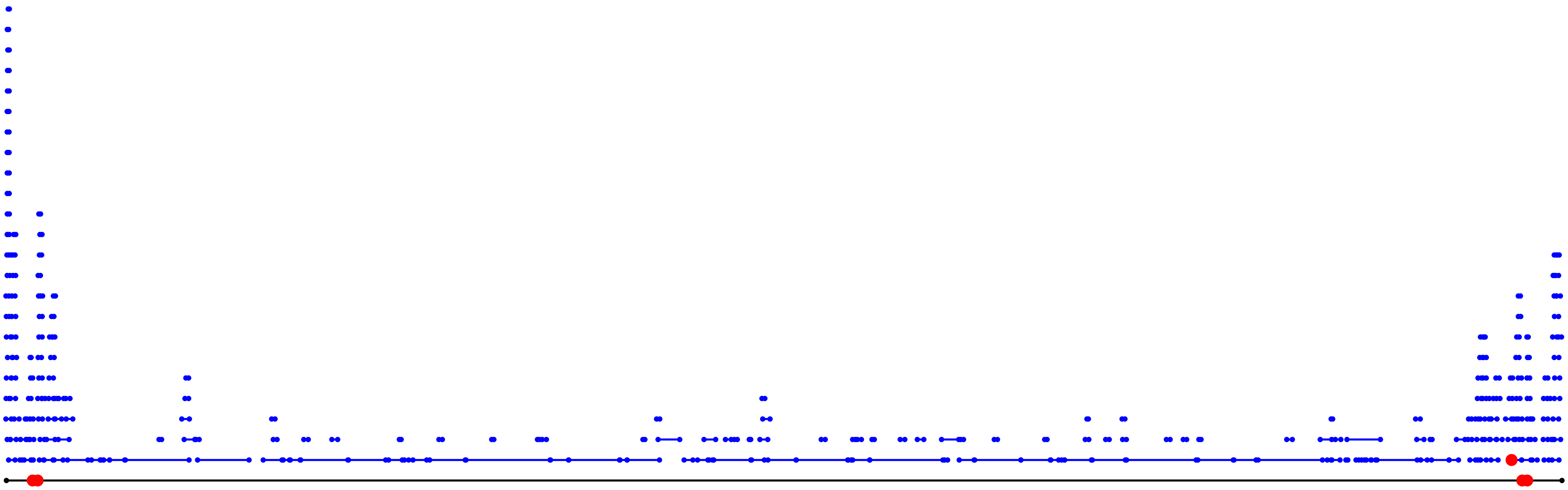

326_1 Chrm 10

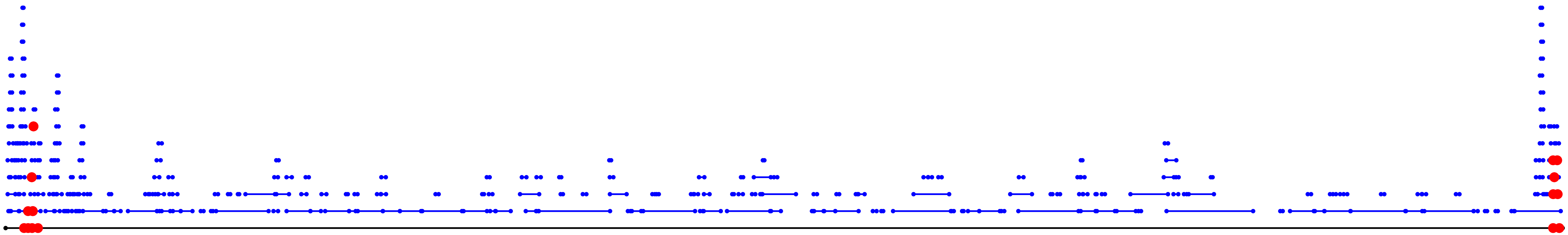

326_1 Chrm 11

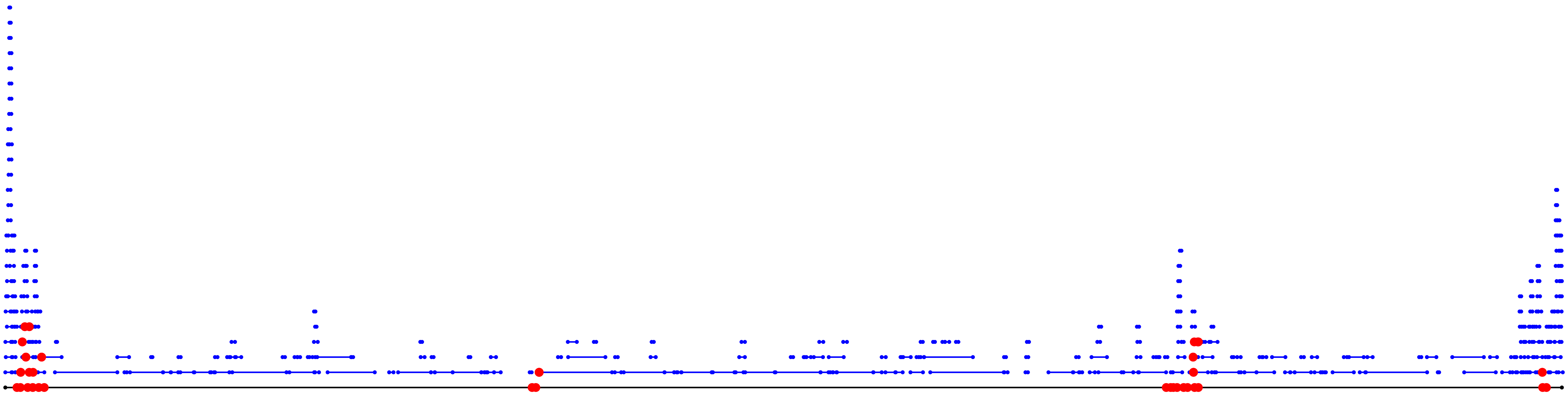

326_1 Chrm 12

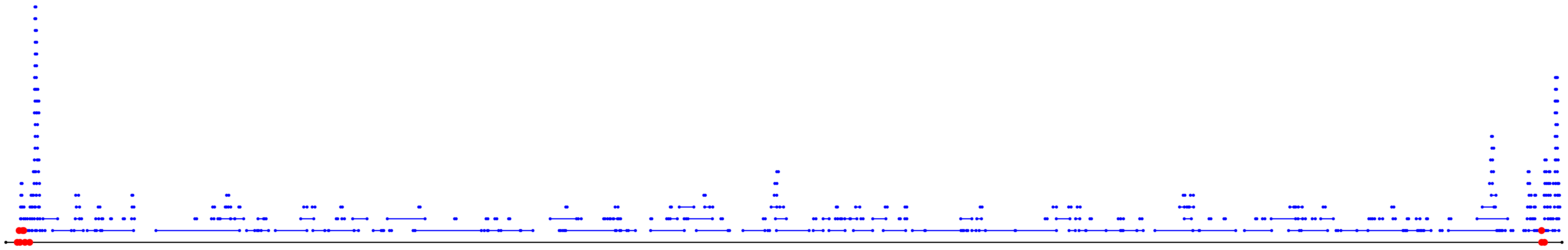

326_1 Chrm 13

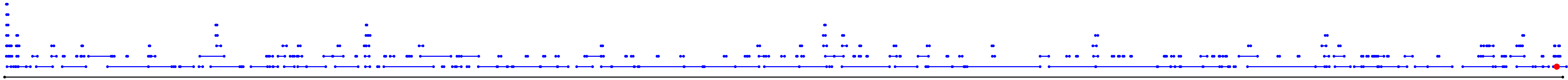

326_1 Chrm 14

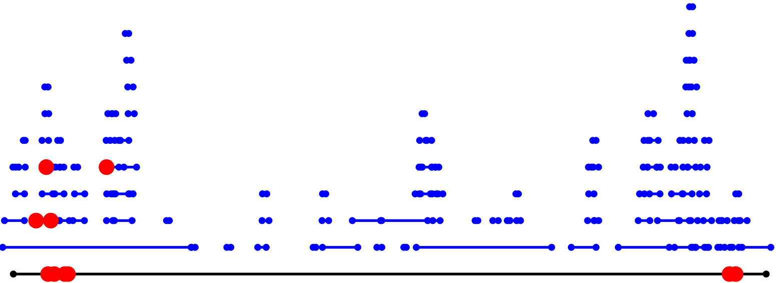

327_1 Chrm 1

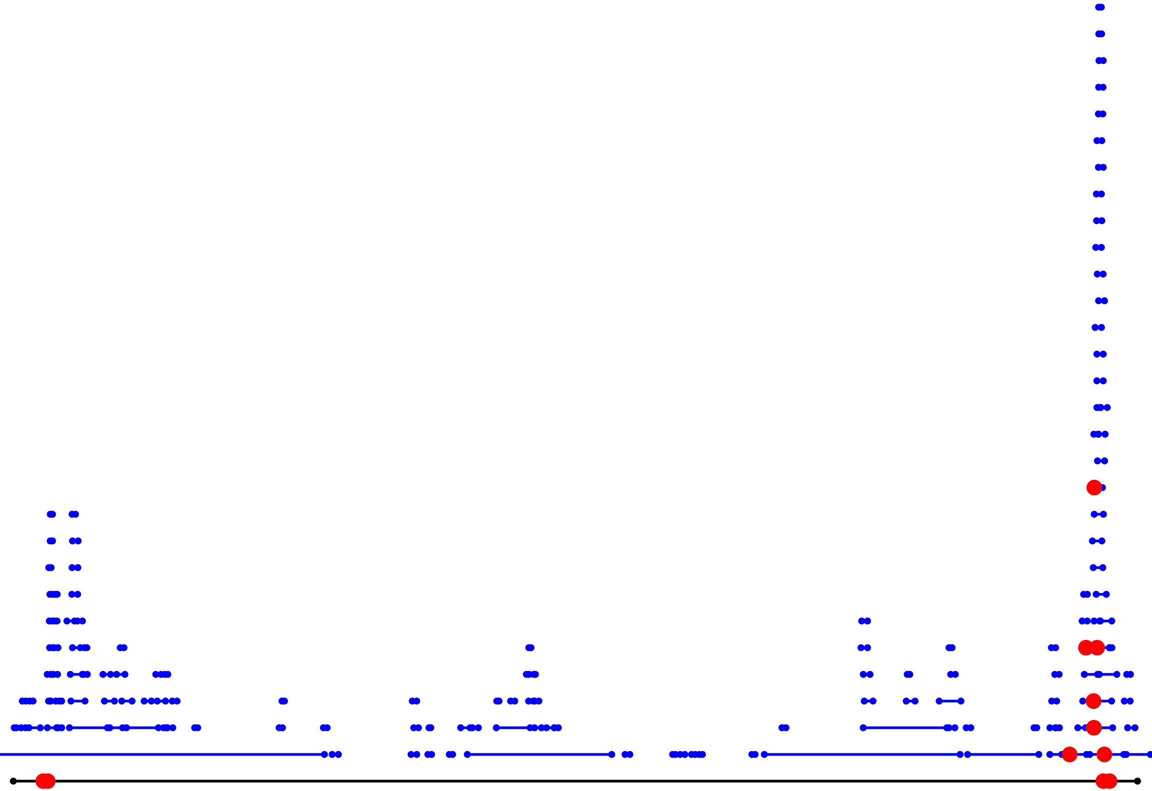

327_1 Chrm 2

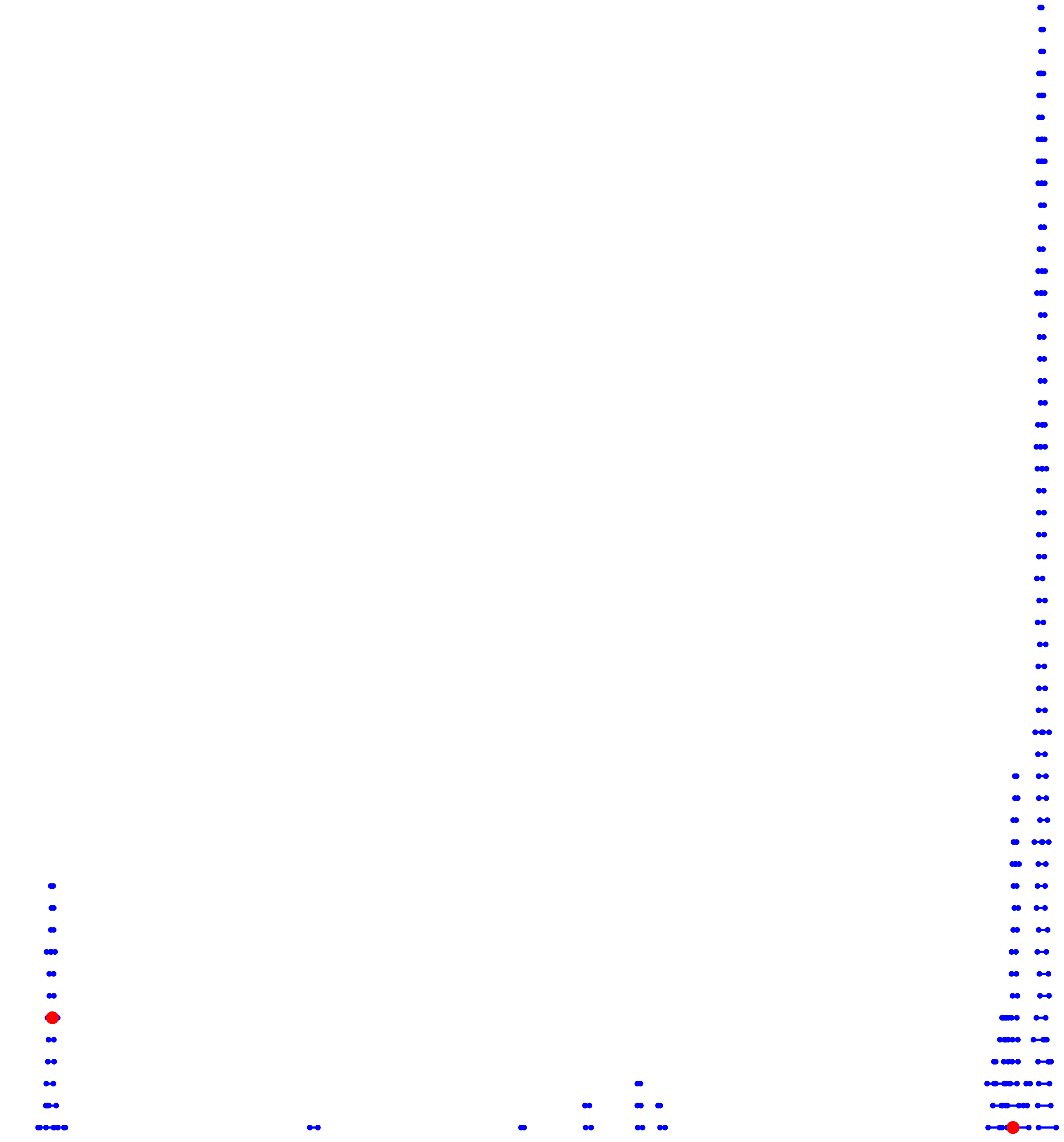

327_1 Chrm 3

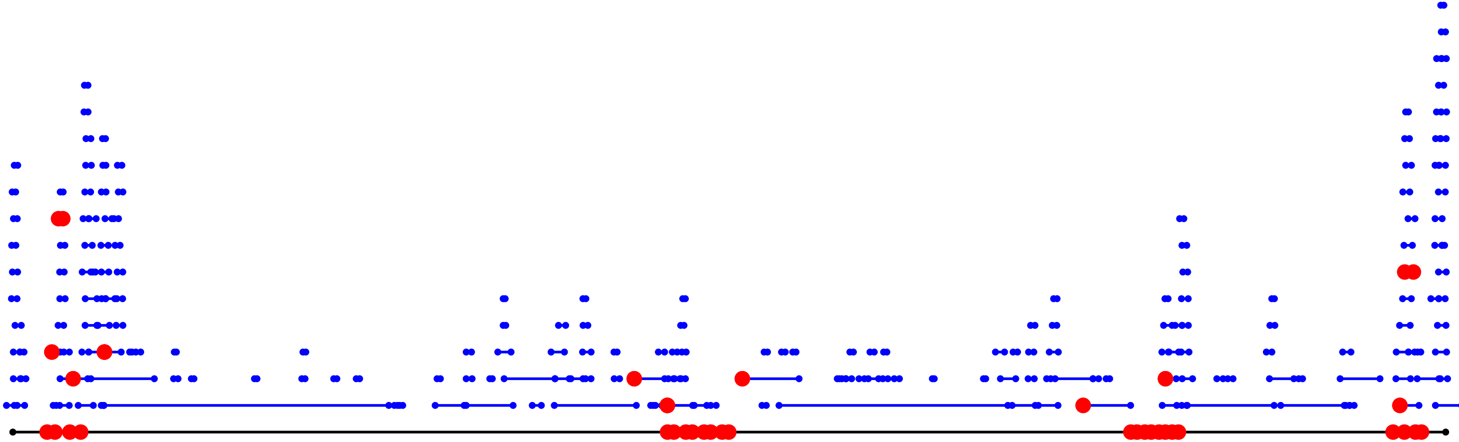

327_1 Chrm 4

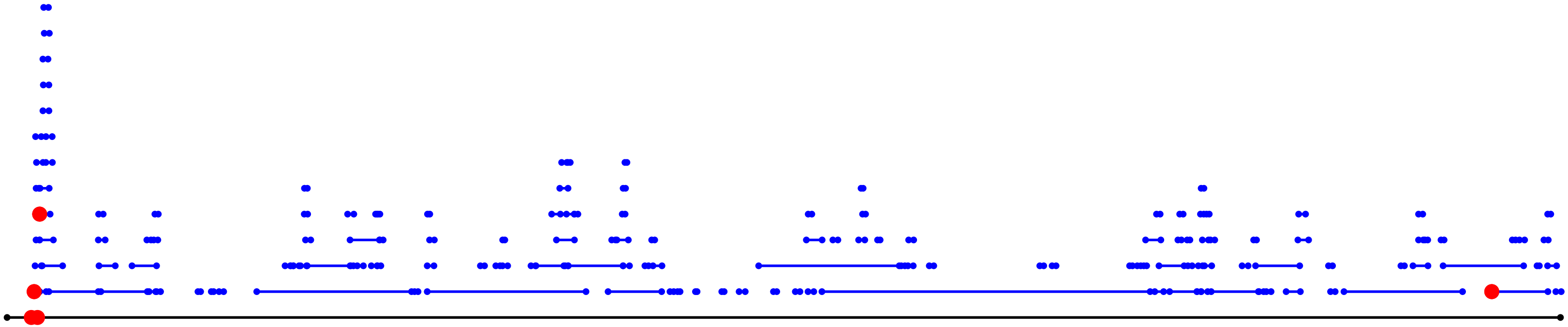

327_1 Chrm 5

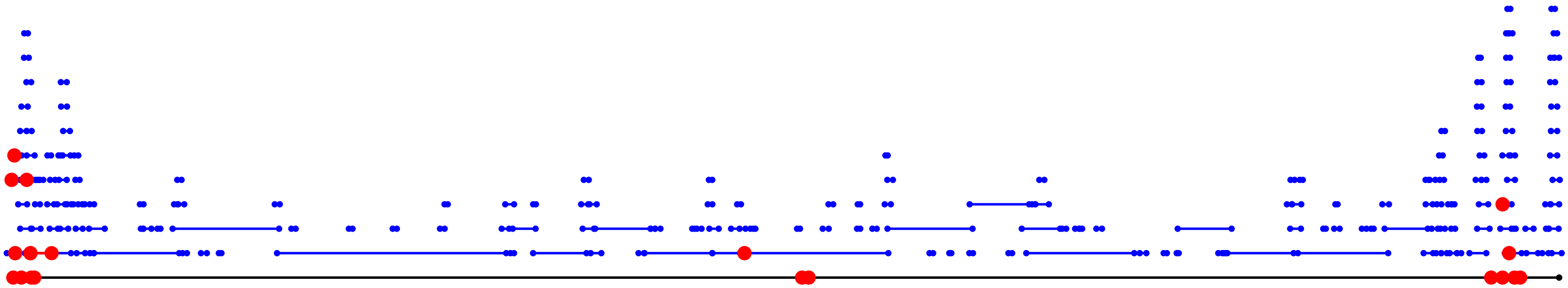

327_1 Chrm 6

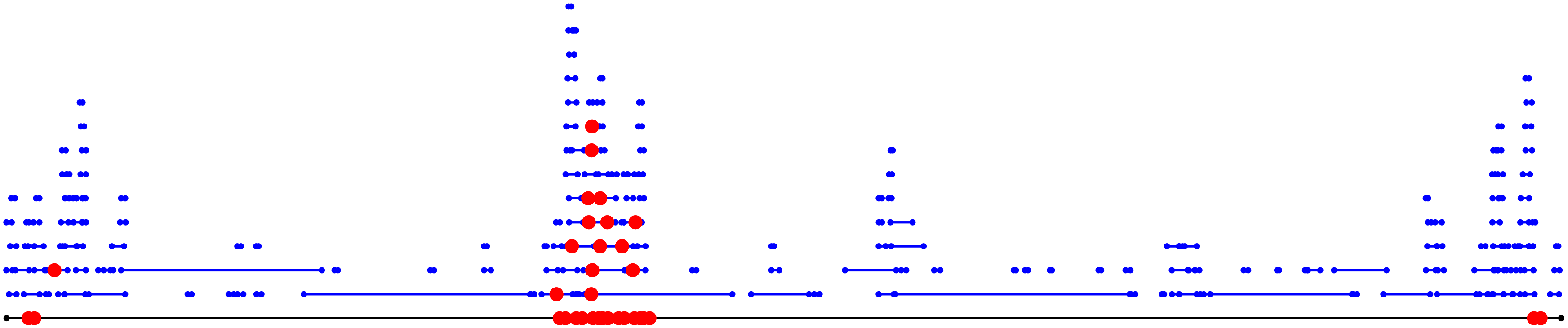

327_1 Chrm 7

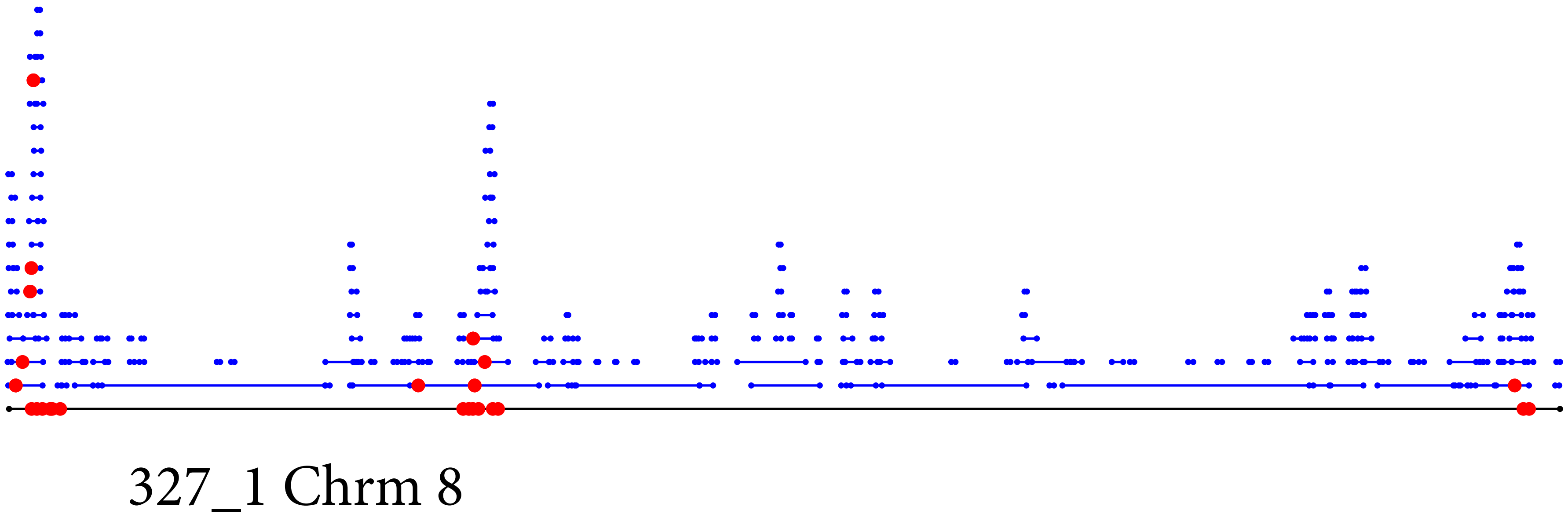
327_1 Chrm 8

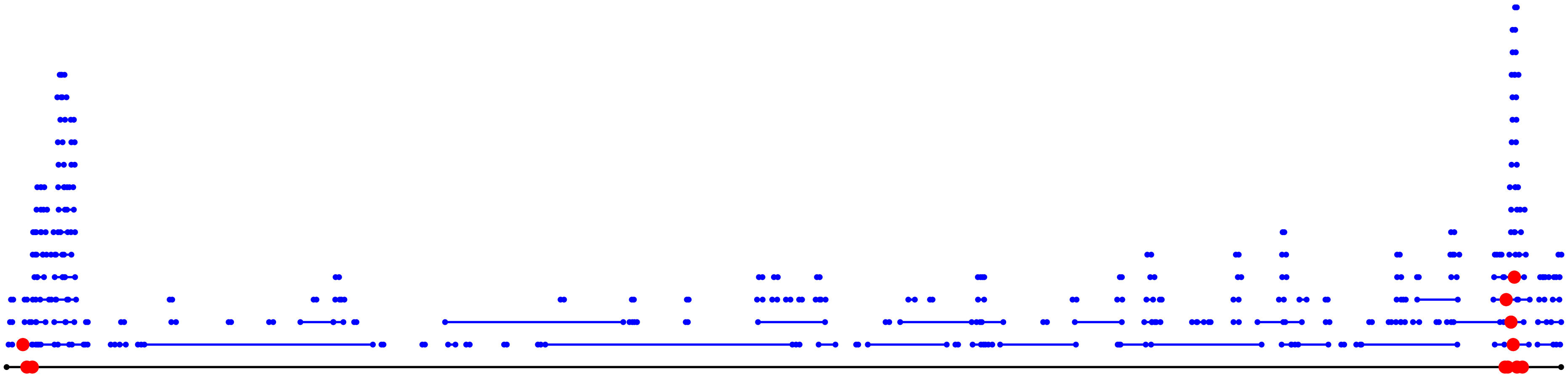

327_1 Chrm 9

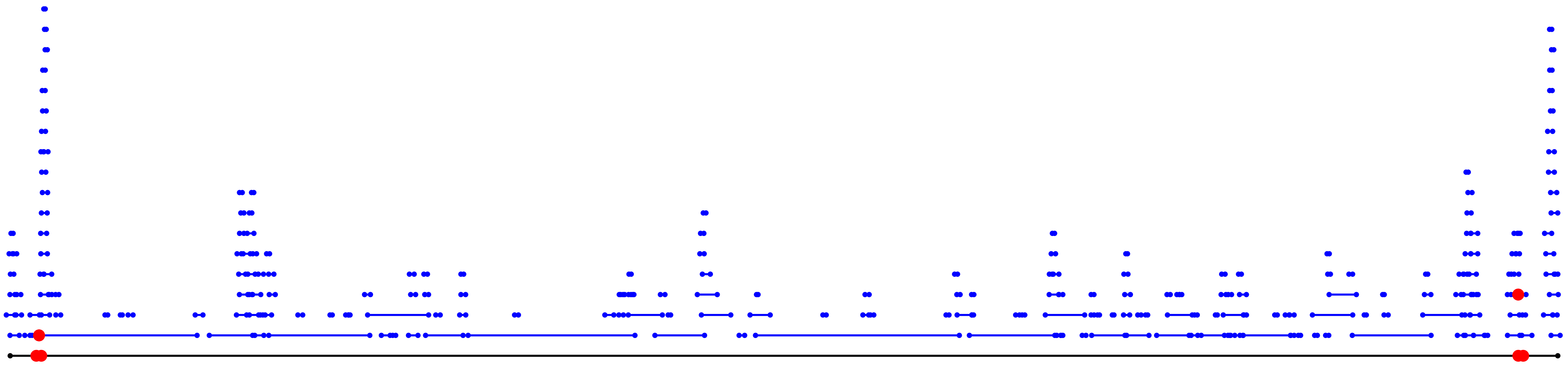

327_1 Chrm 10

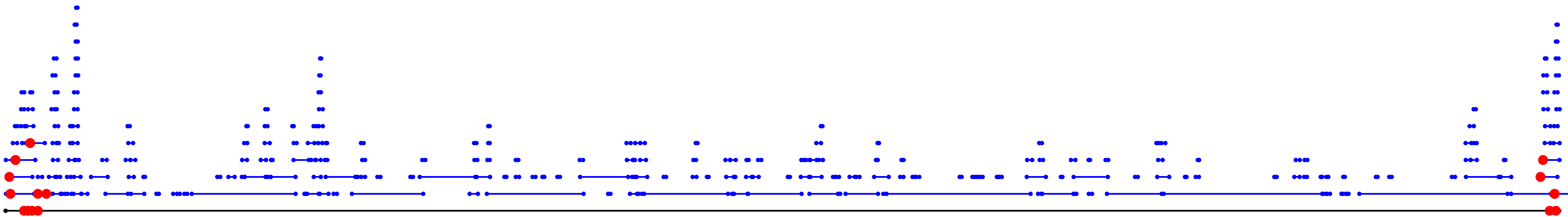

327_1 Chrm 11

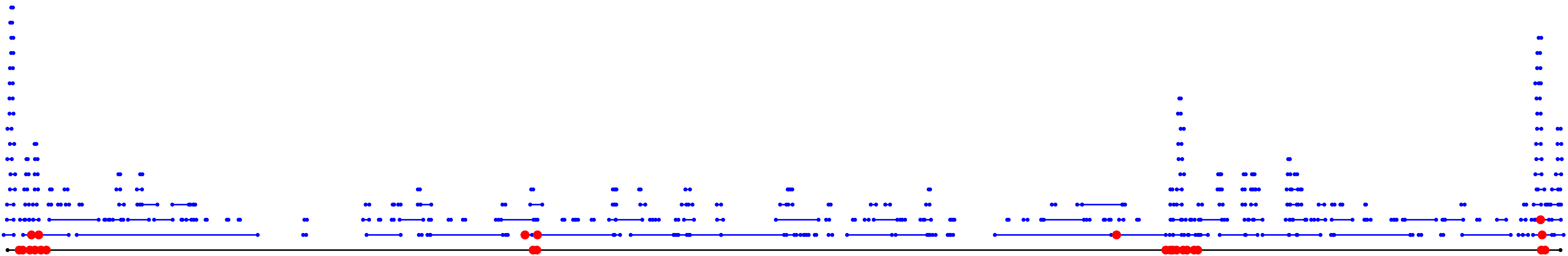

327_1 Chrm 12

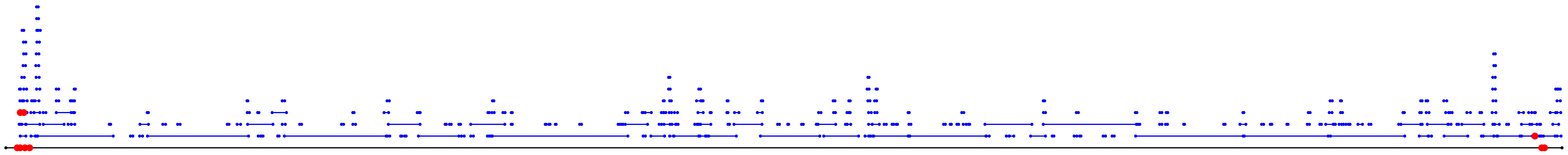

327_1 Chrm 13

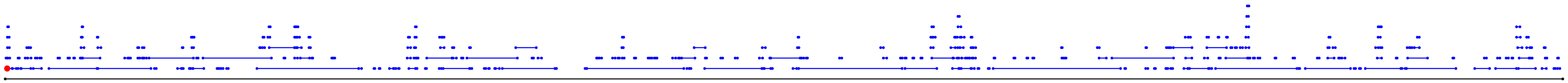

327_1 Chrm 14

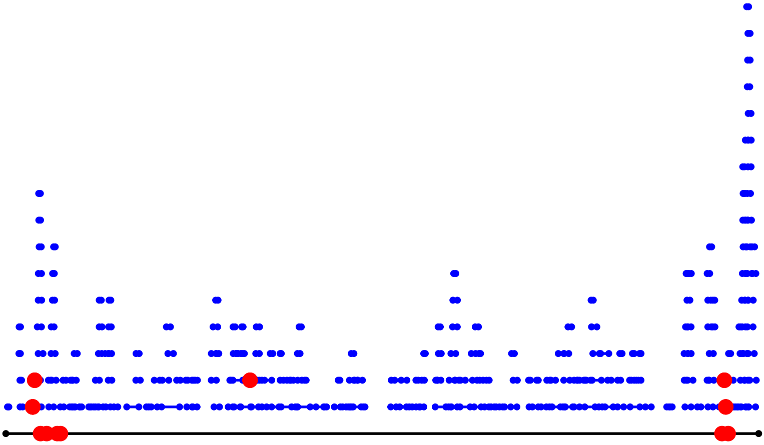

365_1 Chrm 1

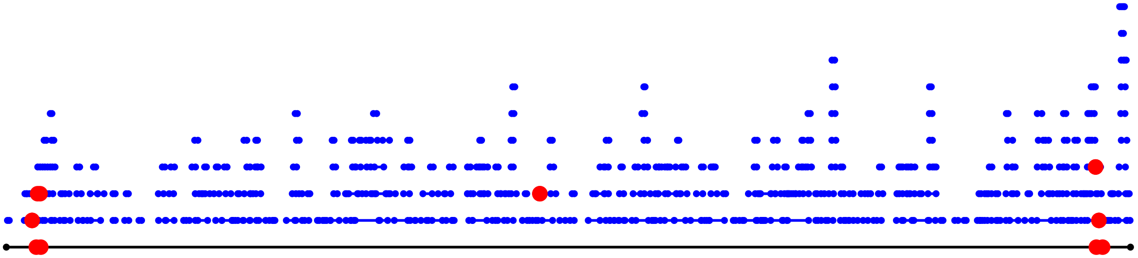

365_1 Chrm 2

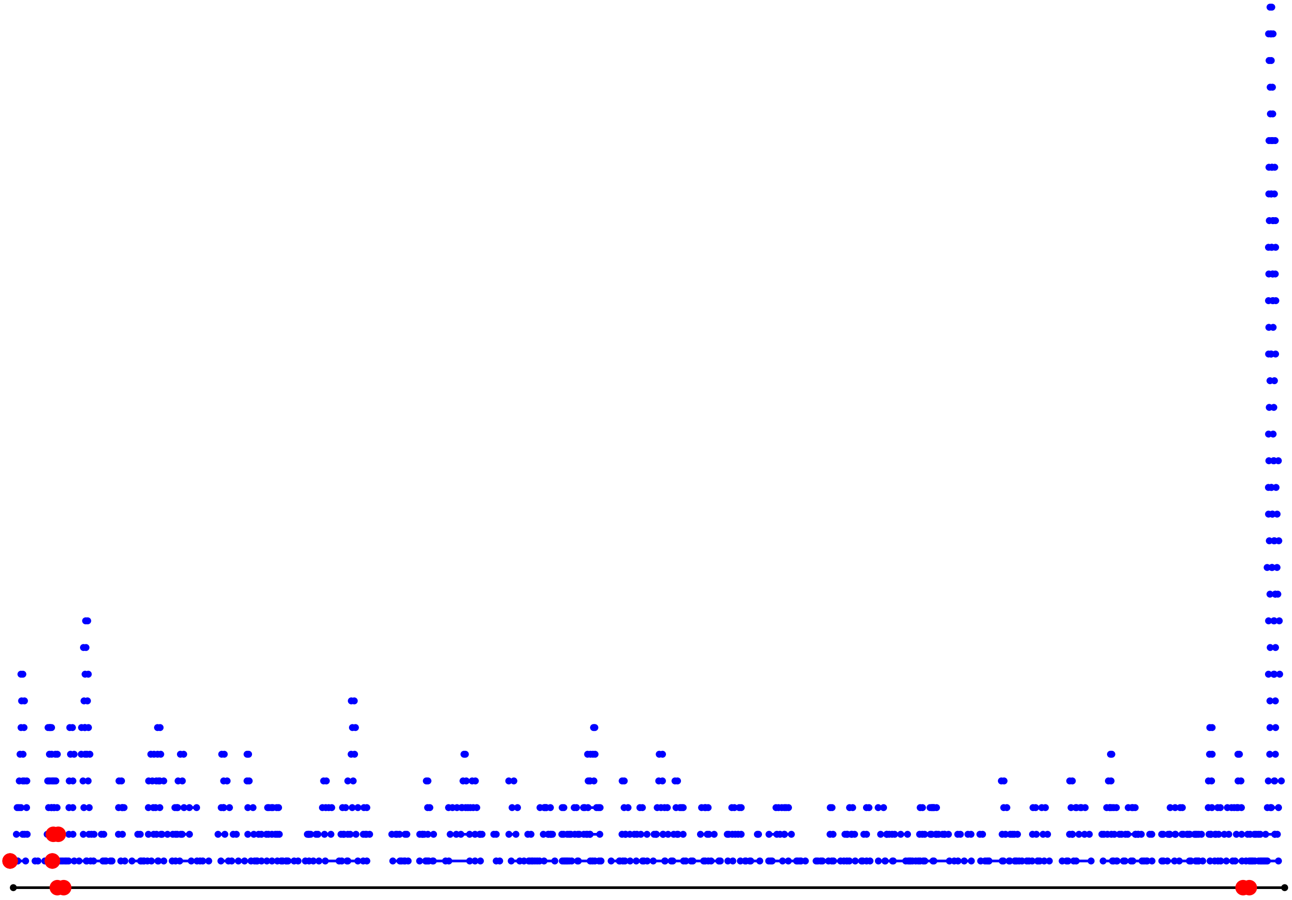

365_1 Chrm 3

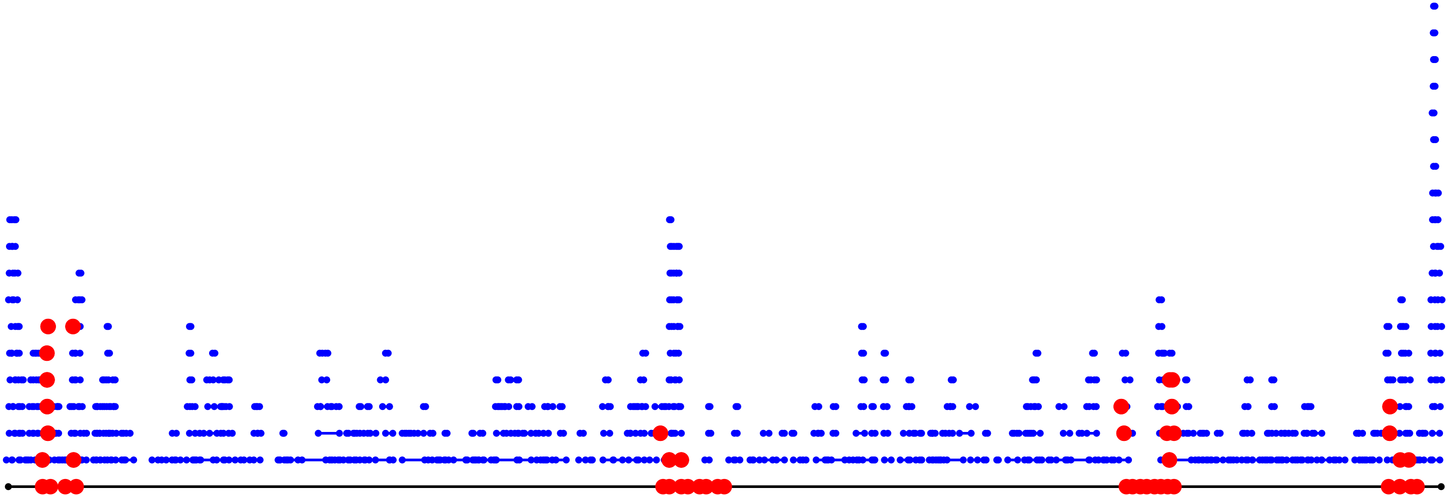

365_1 Chrm 4

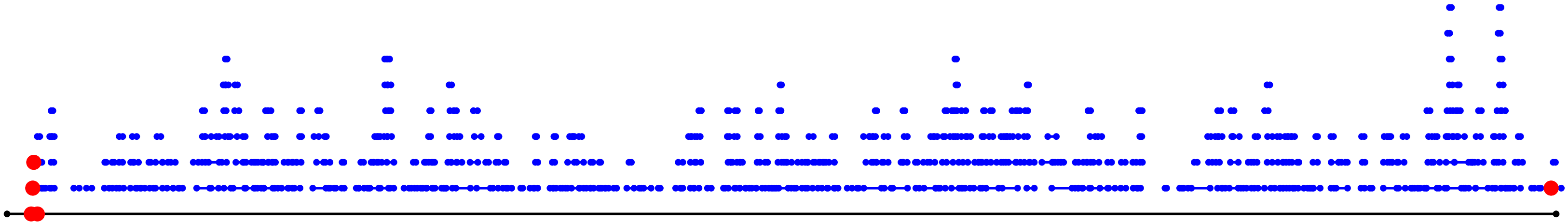

365_1 Chrm 5

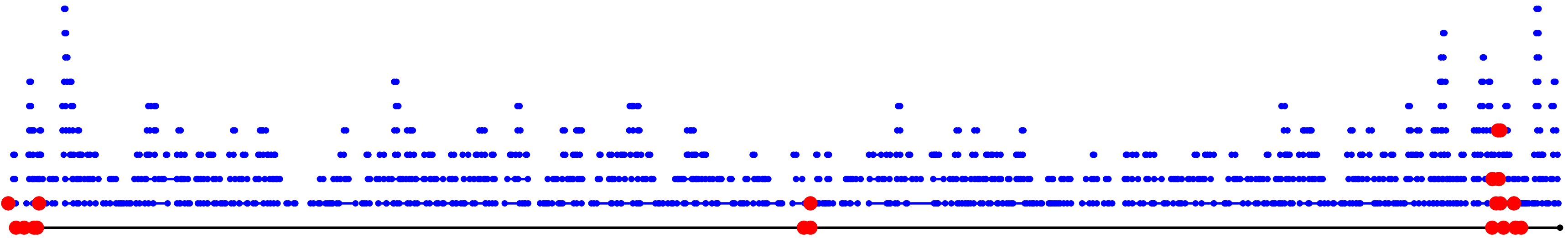

365_1 Chrm 6

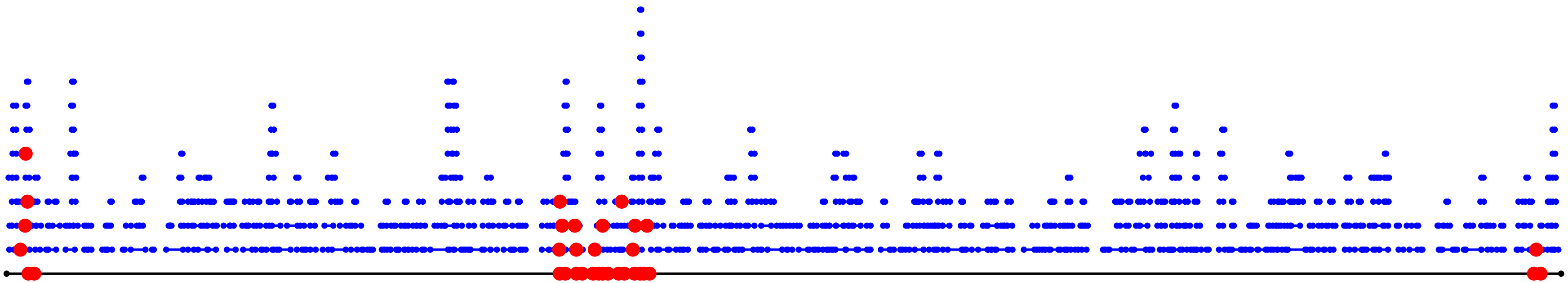

365_1 Chrm 7

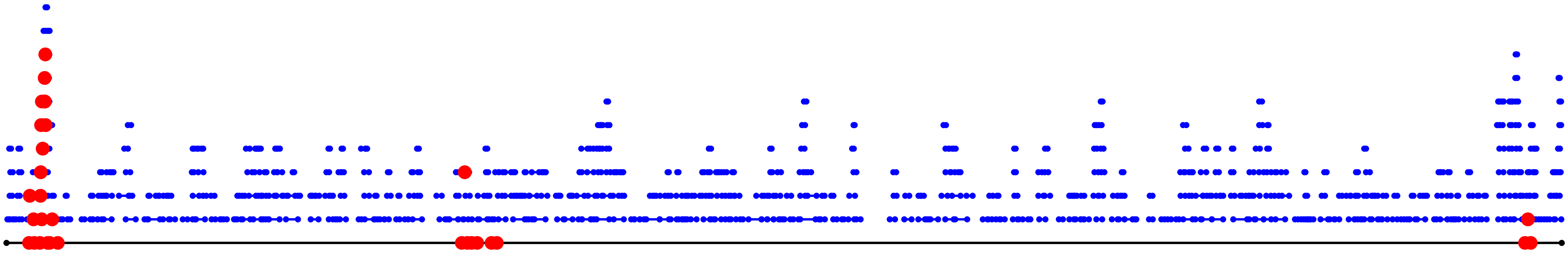

365_1 Chrm 8

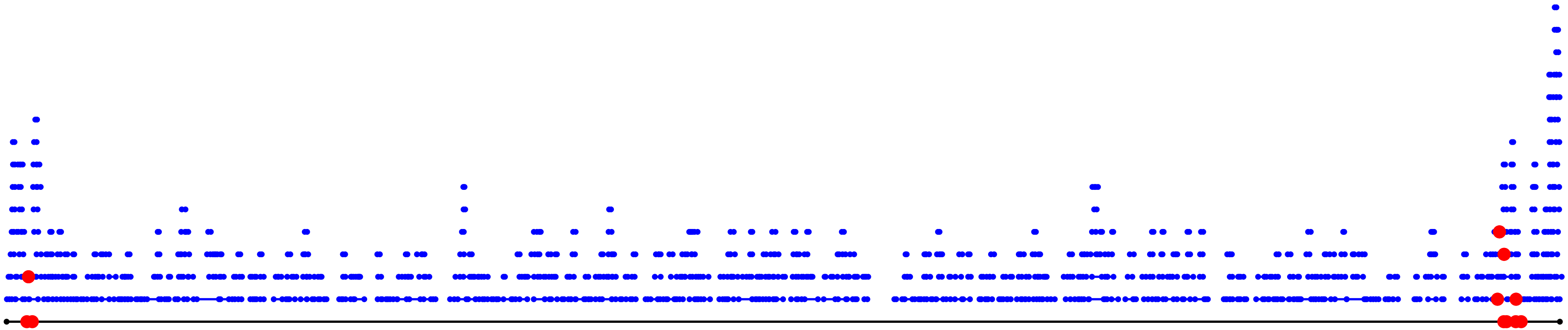

365_1 Chrm 9

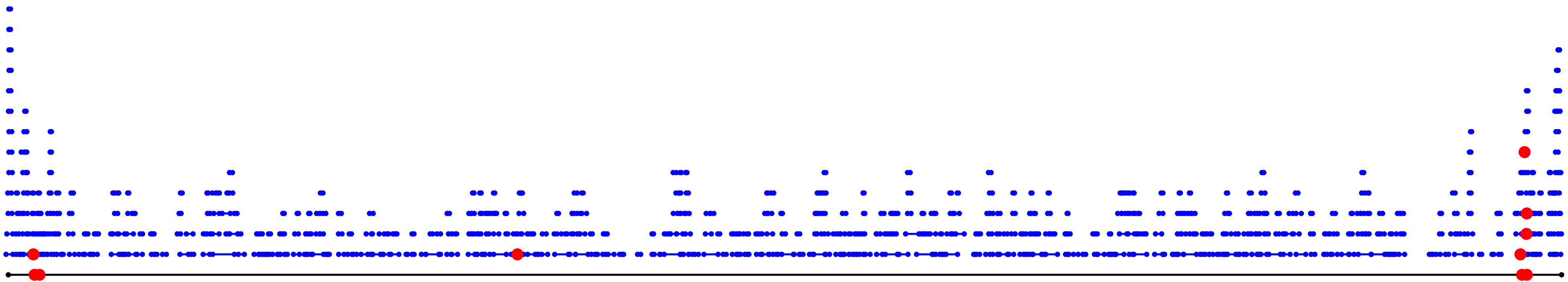

365_1 Chrm 10

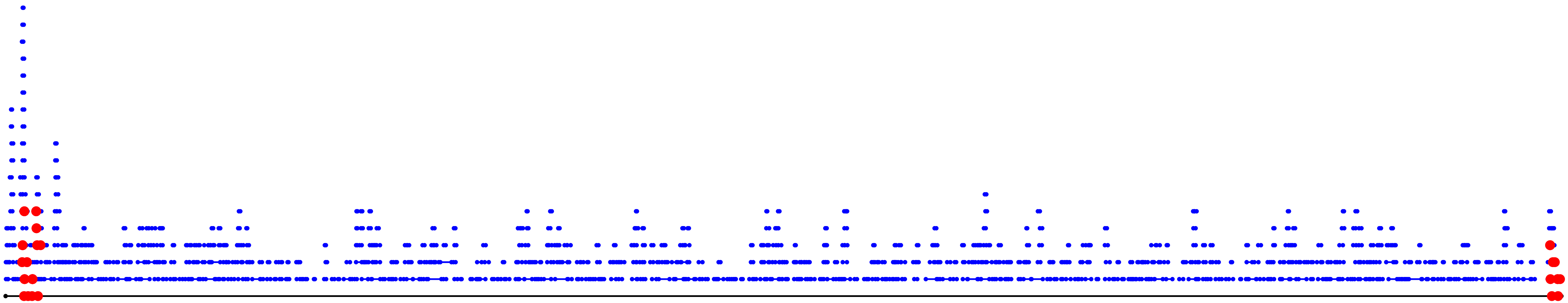

365_1 Chrm 11

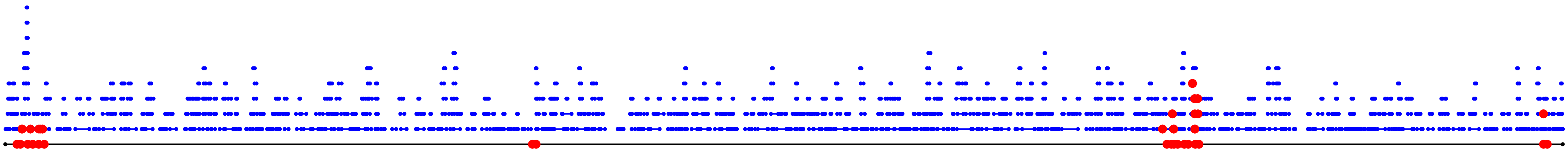

365_1 Chrm 12

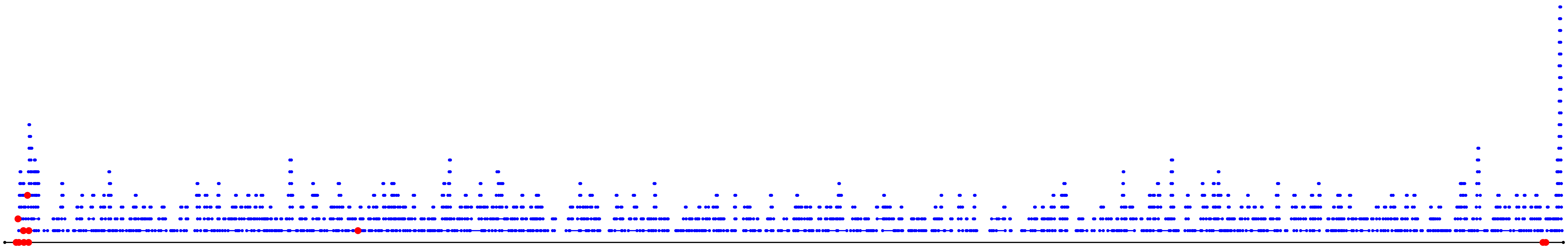

365_1 Chrm 13

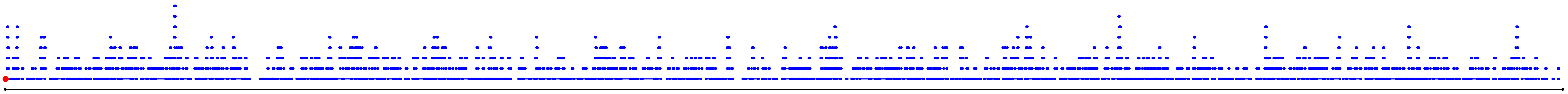

365_1 Chrm 14

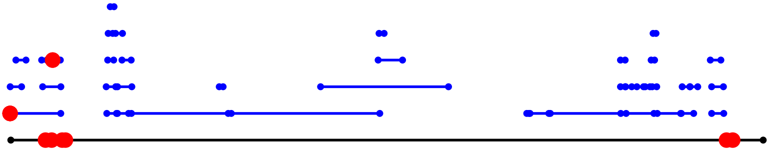

366_1 Chrm 1

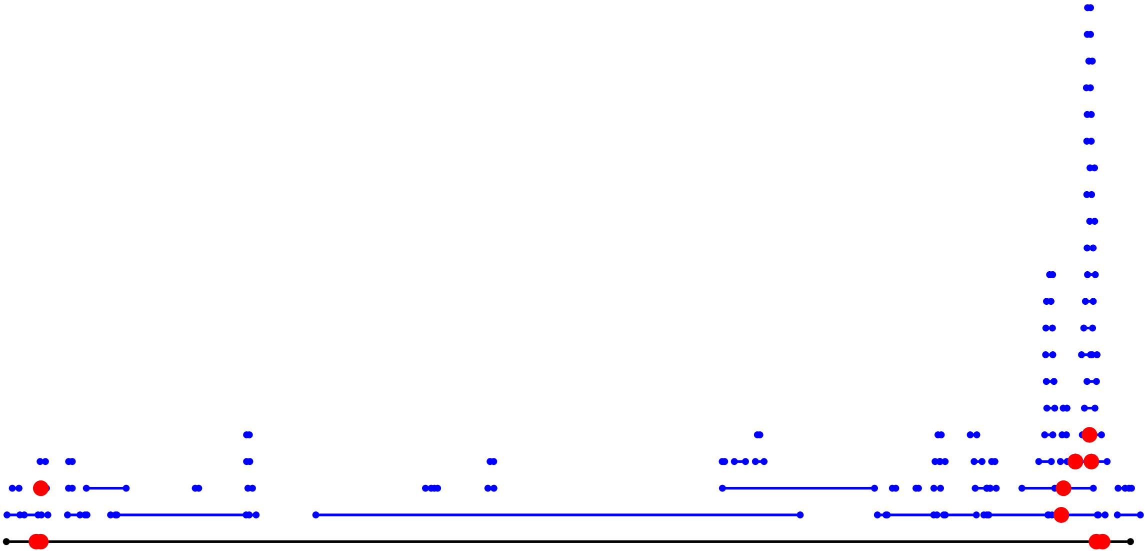

366_1 Chrm 2

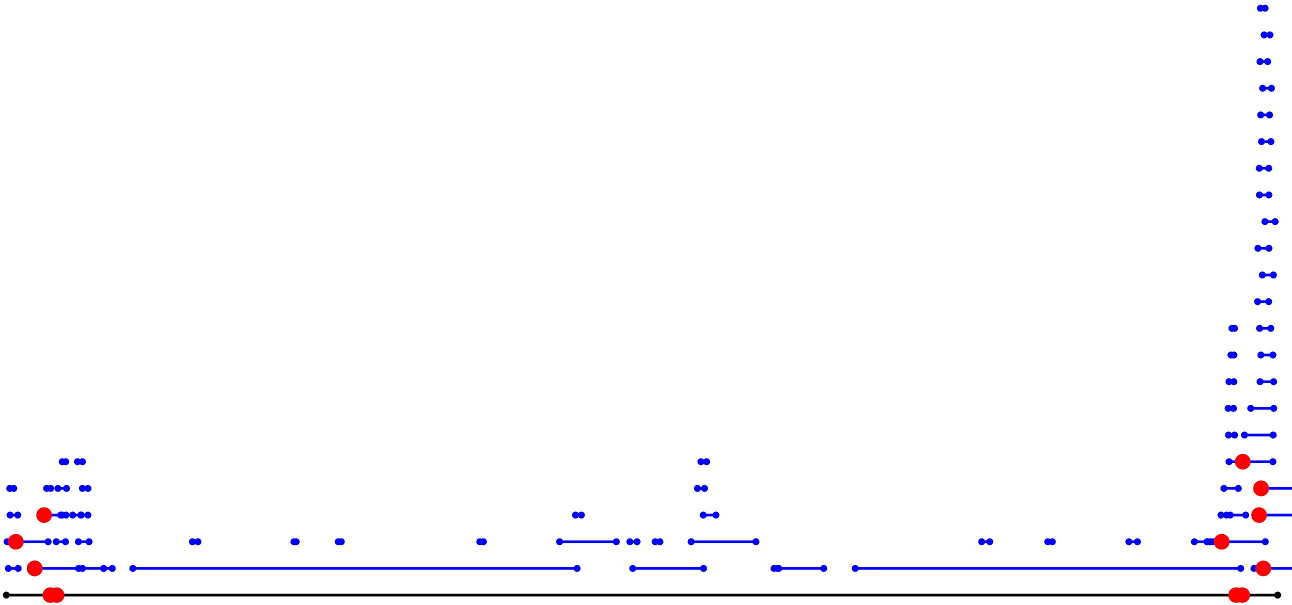

366_1 Chrm 3

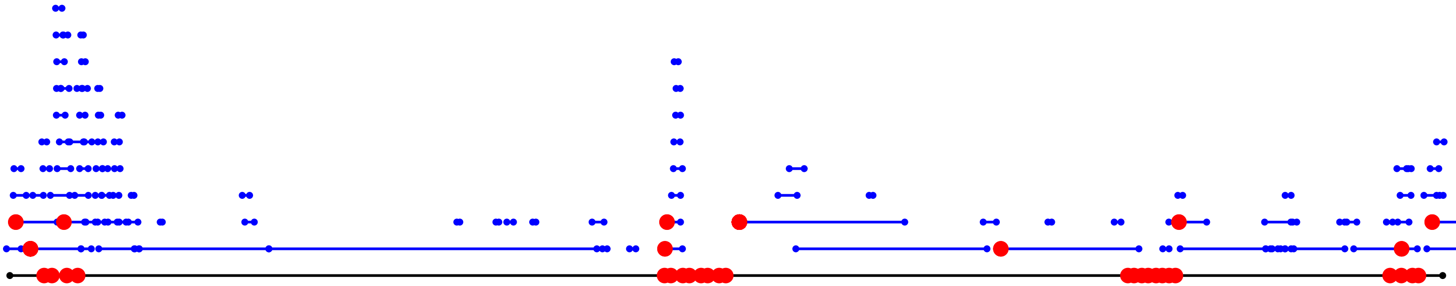

366_1 Chrm 4

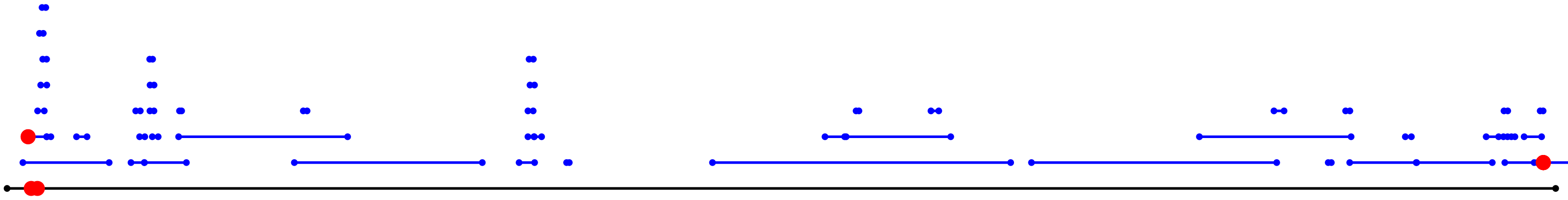

366_1 Chrm 5

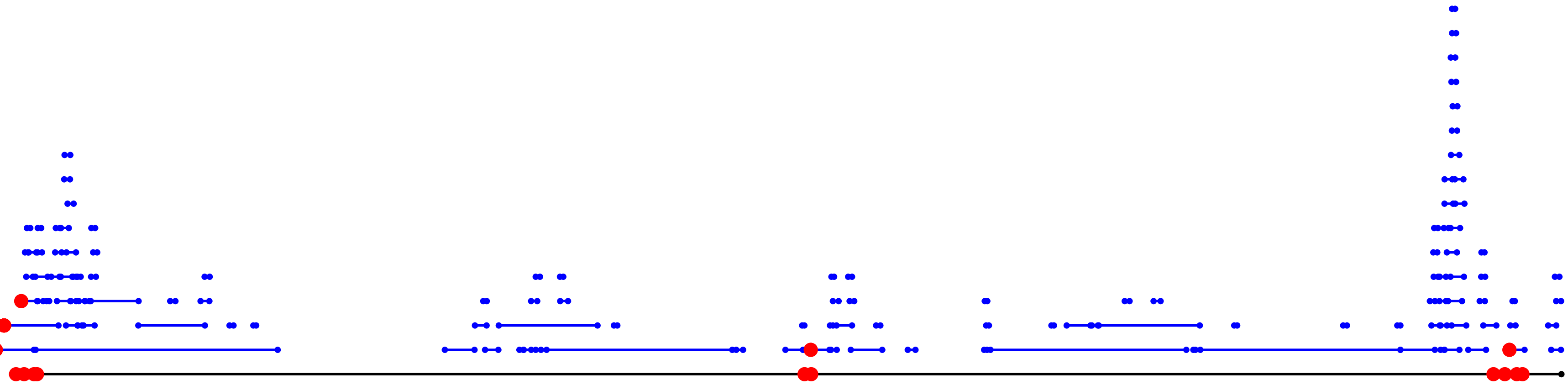

366_1 Chrm 6

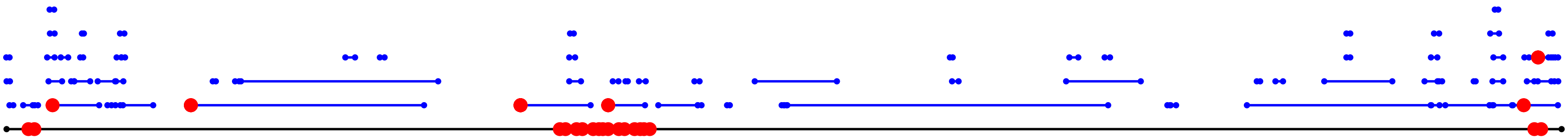

366_1 Chrm 7

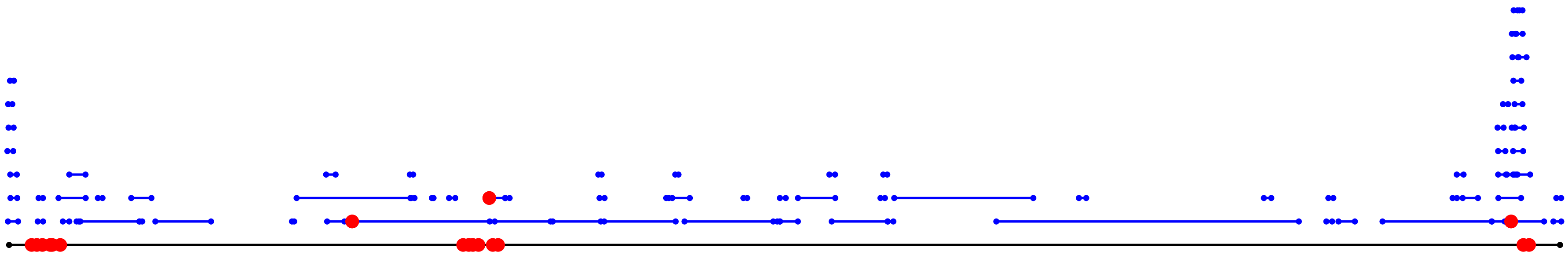

366_1 Chrm 8

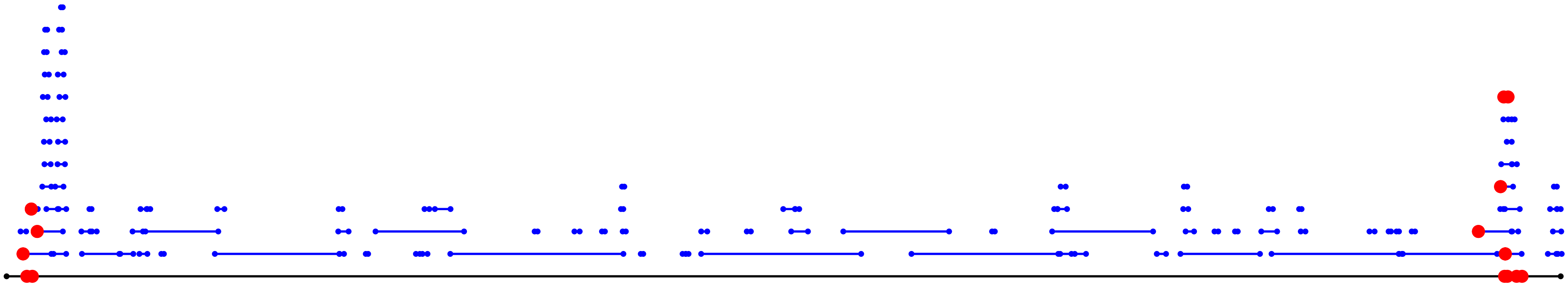

366_1 Chrm 9

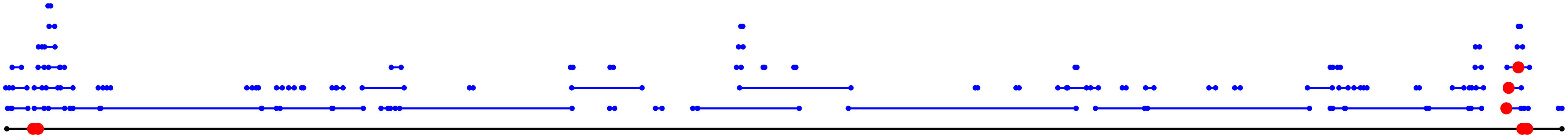

366_1 Chrm 10

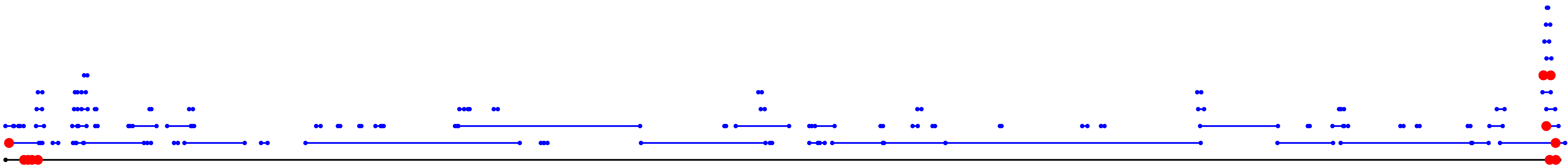

366_1 Chrm 11

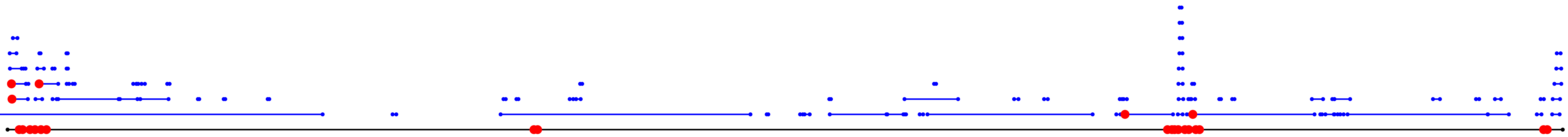

366_1 Chrm 12

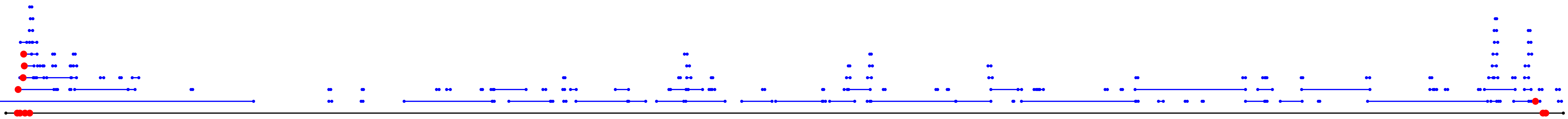

366_1 Chrm 13

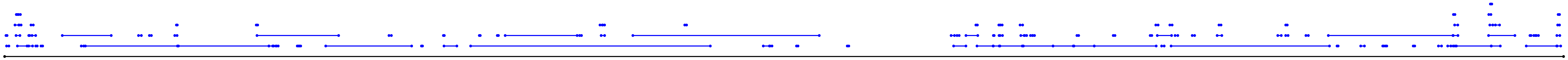

366_1 Chrm 14

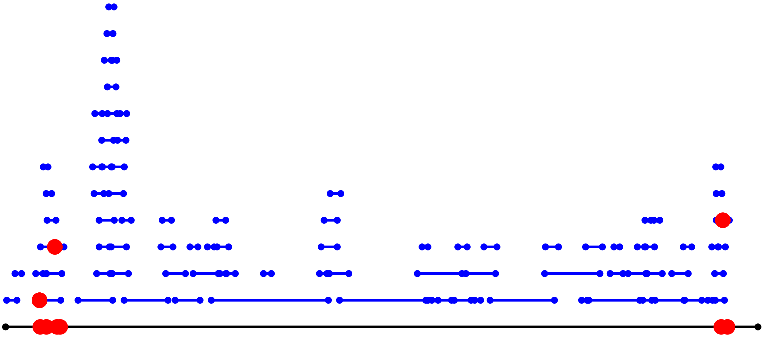

377_1 Chrm 1

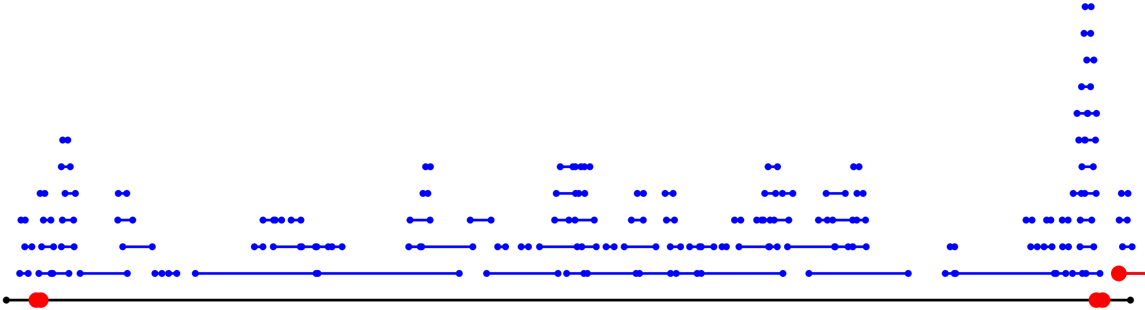

377_1 Chrm 2

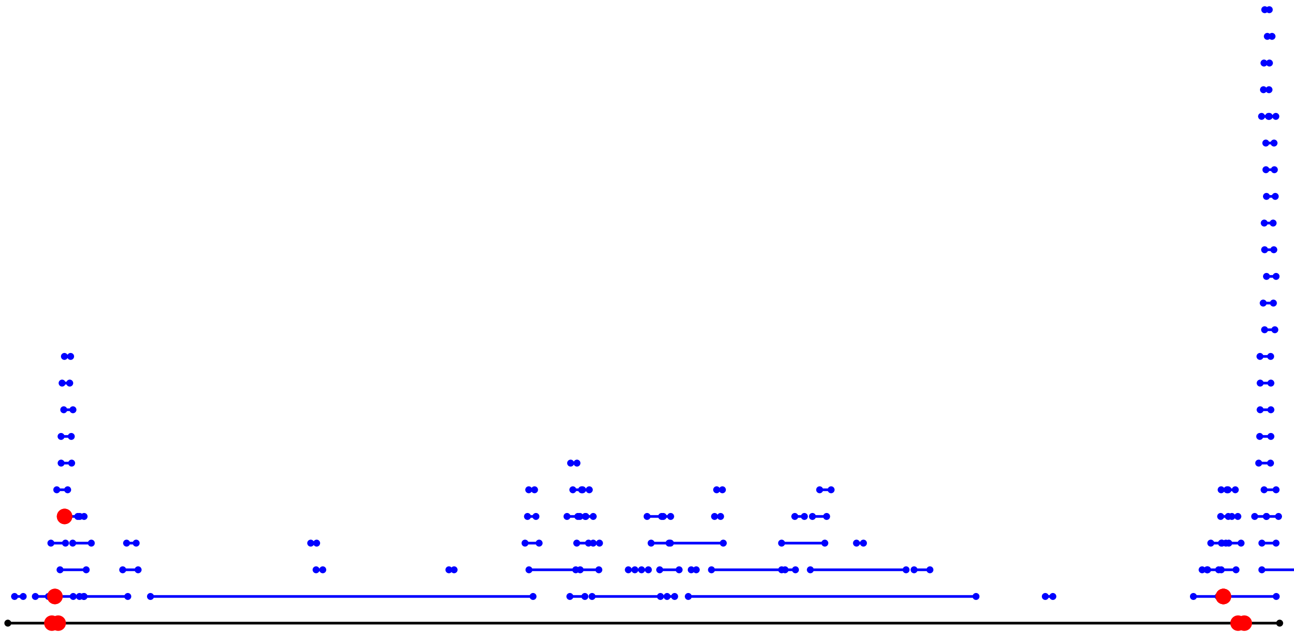

377_1 Chrm 3

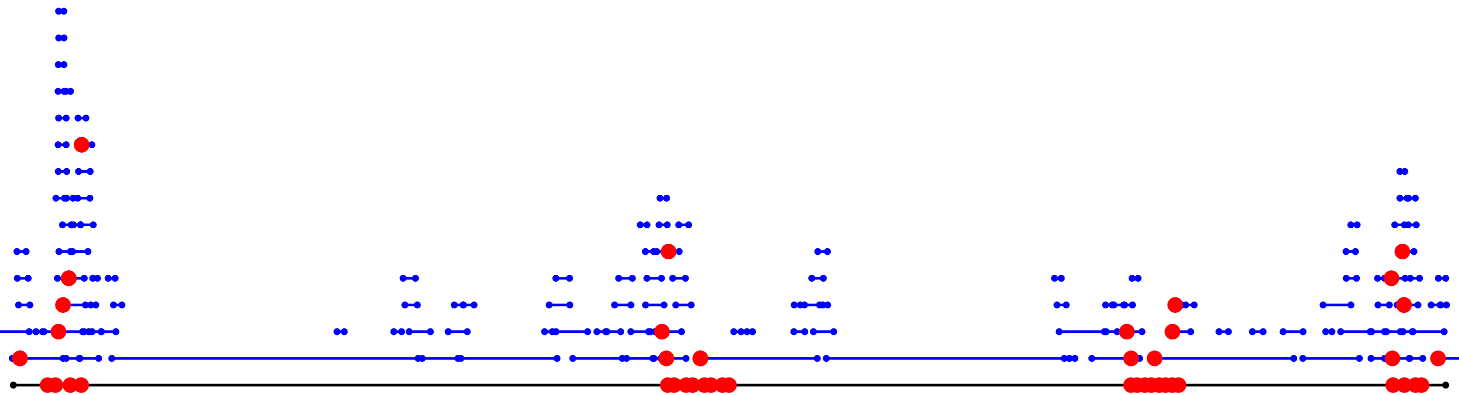

377_1 Chrm 4

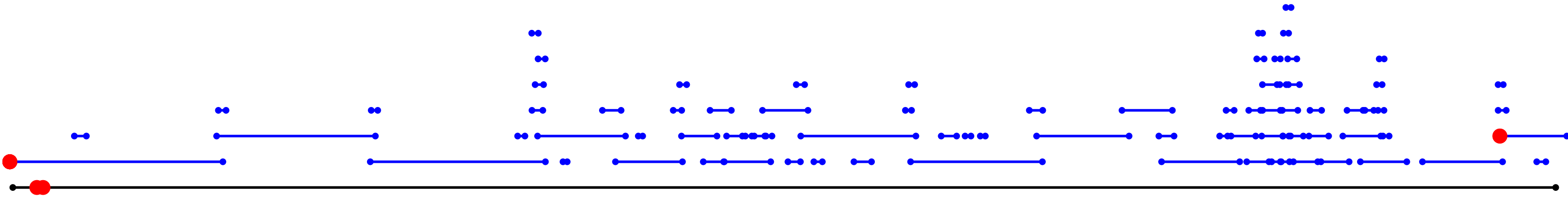

377_1 Chrm 5

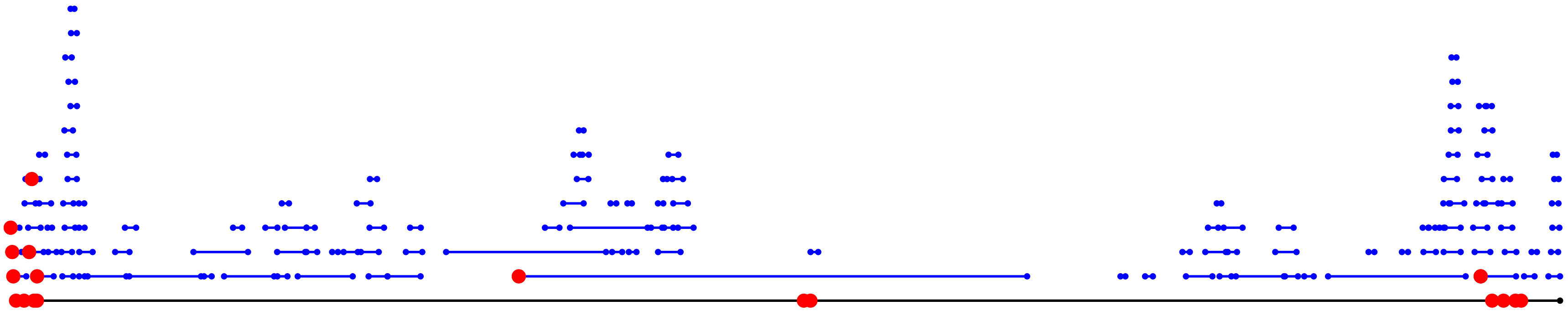

377_1 Chrm 6

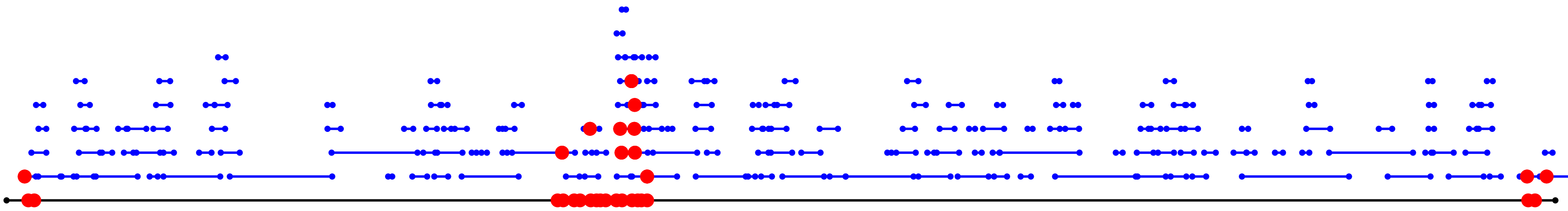

377_1 Chrm 7

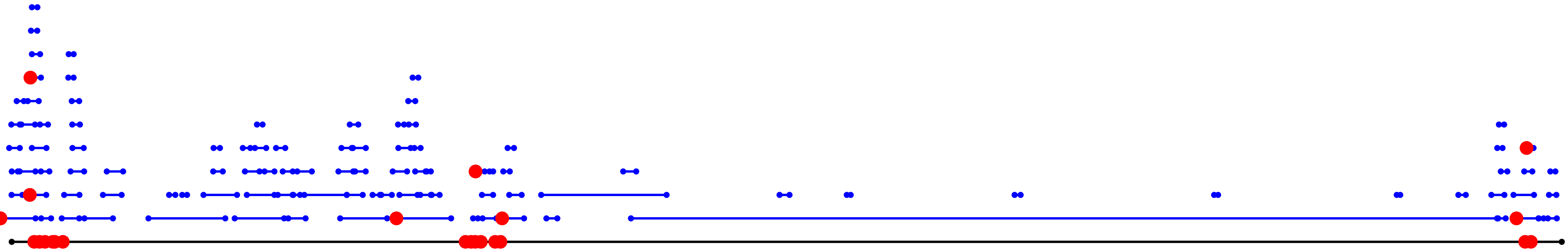

377_1 Chrm 8

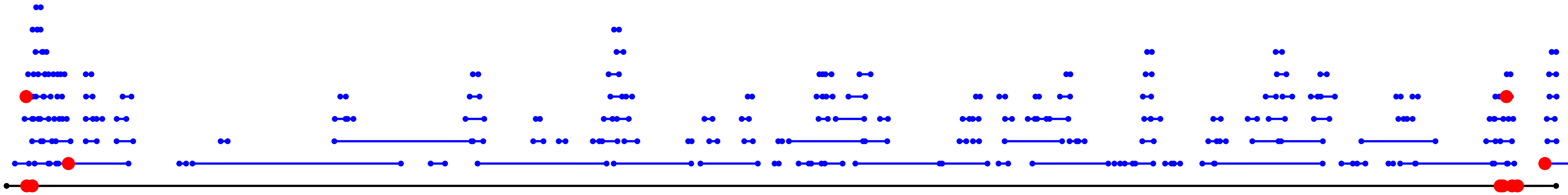

377_1 Chrm 9

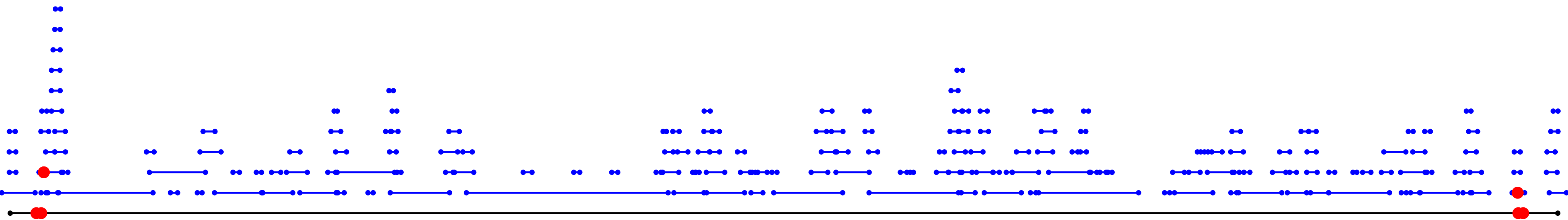

377_1 Chrm 10

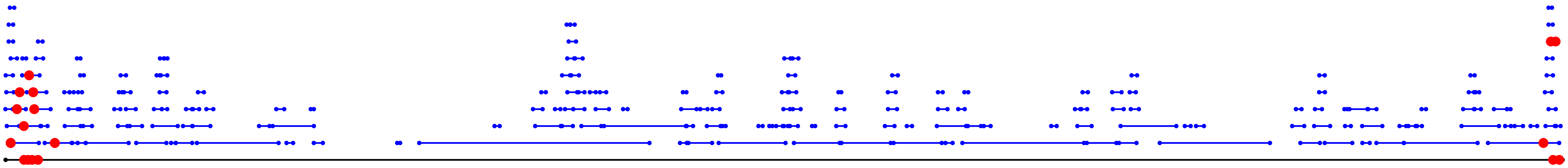

377_1 Chrm 11

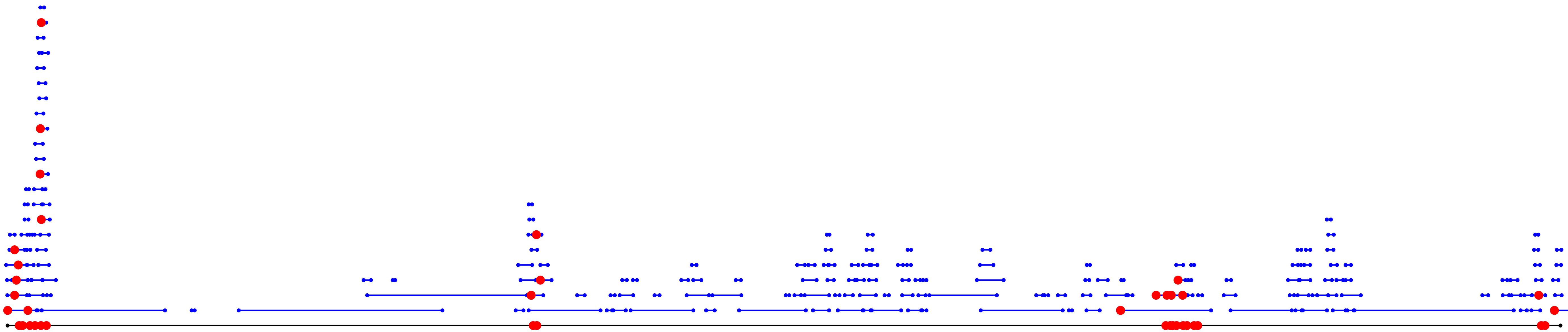

377_1 Chrm 12

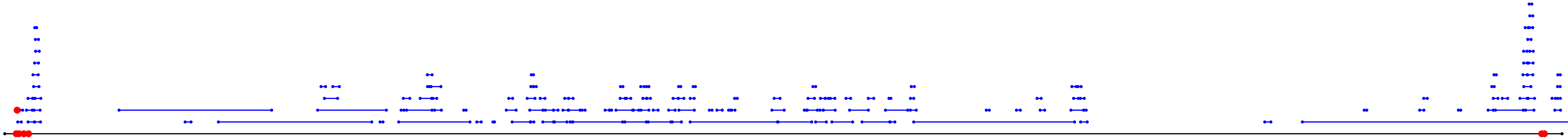

377_1 Chrm 13

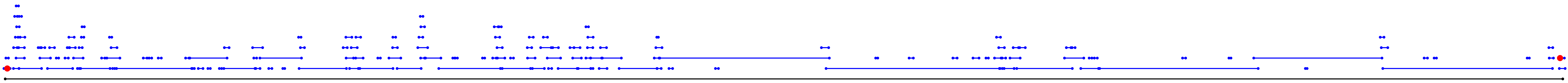

377_1 Chrm 14

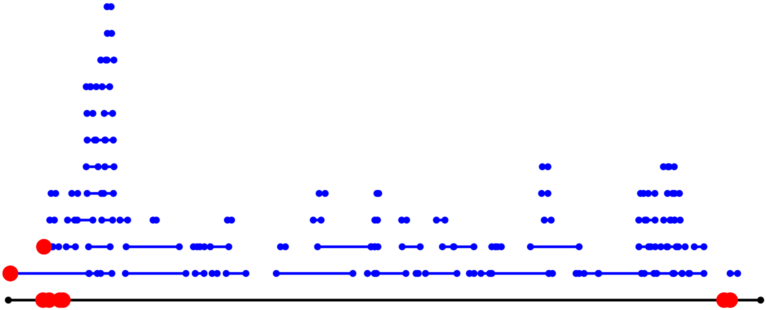

383_1 Chrm 1

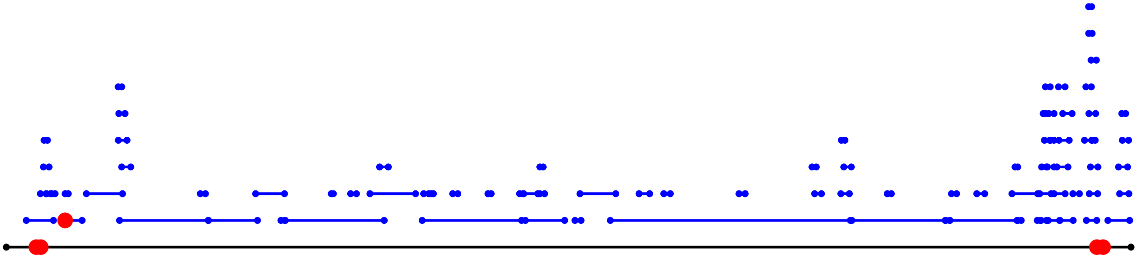

383_1 Chrm 2

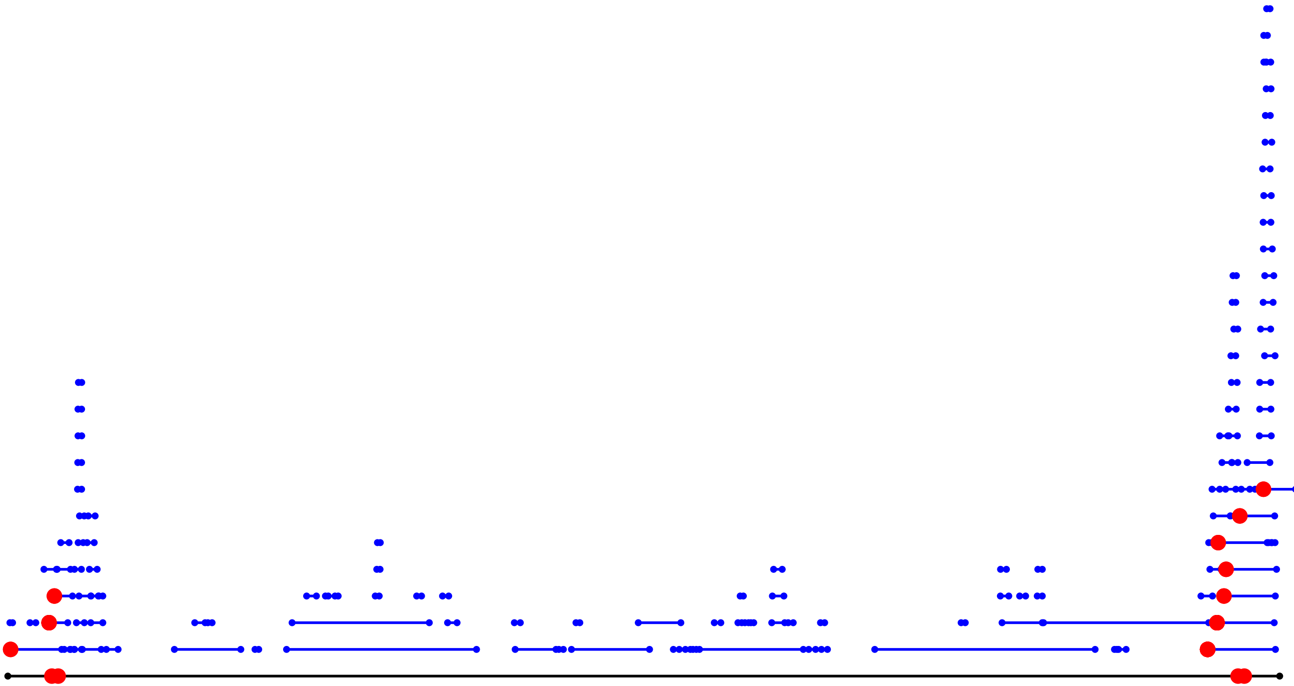

383_1 Chrm 3

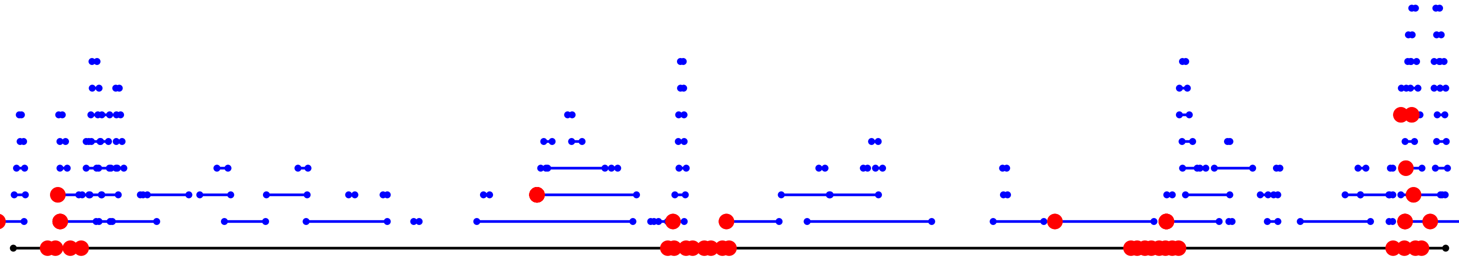

383_1 Chrm 4

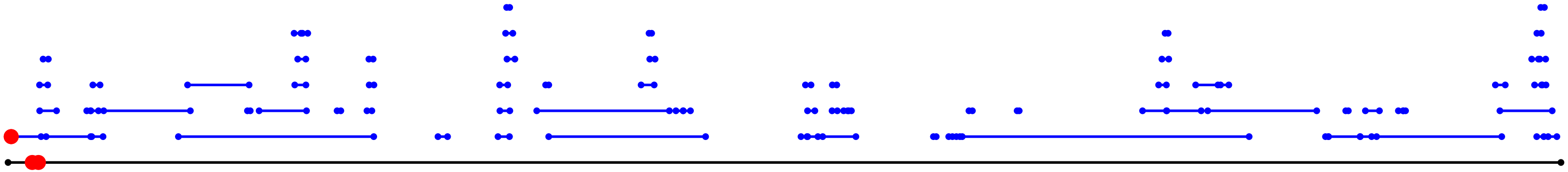

383_1 Chrm 5

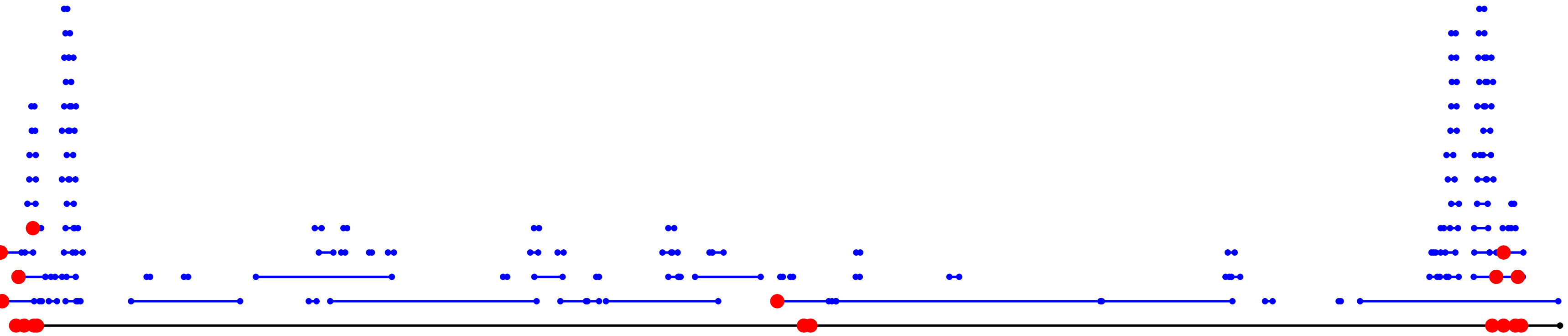

383_1 Chrm 6

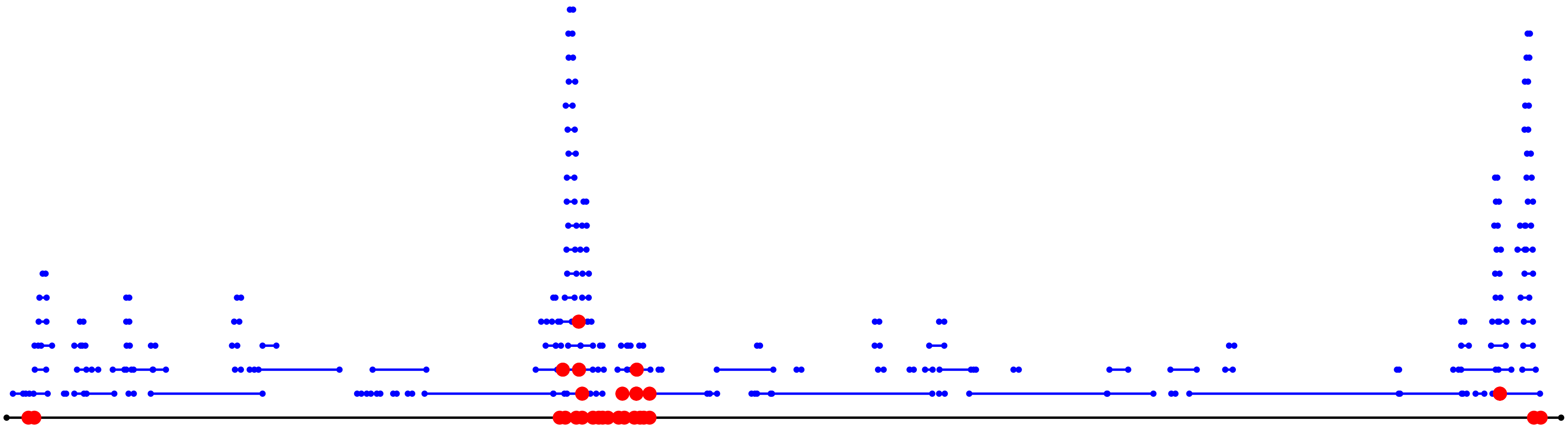

383_1 Chrm 7

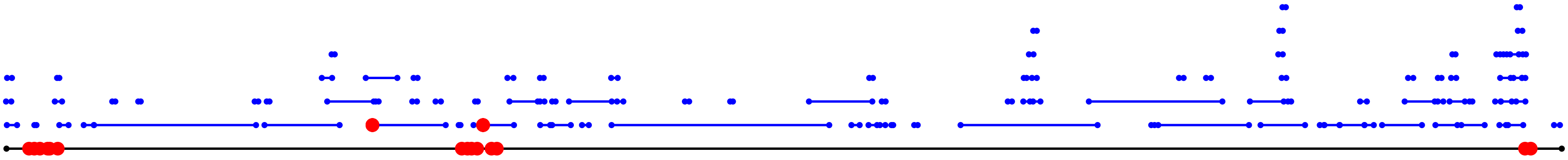

383_1 Chrm 8

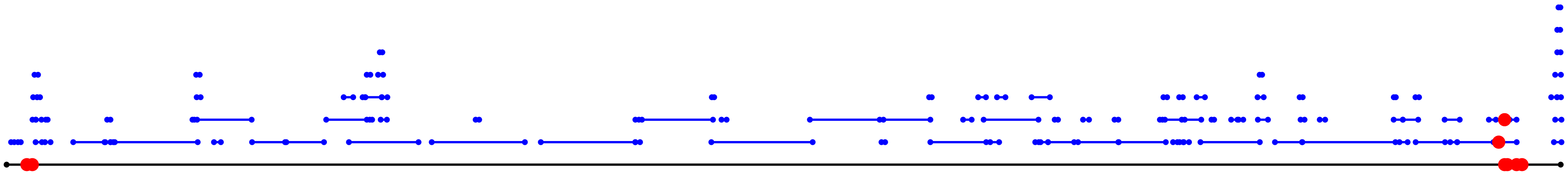

383_1 Chrm 9

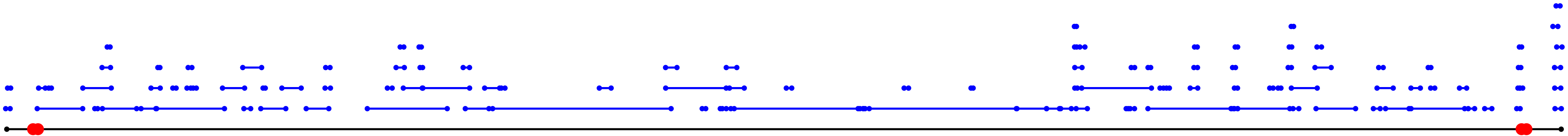

383_1 Chrm 10

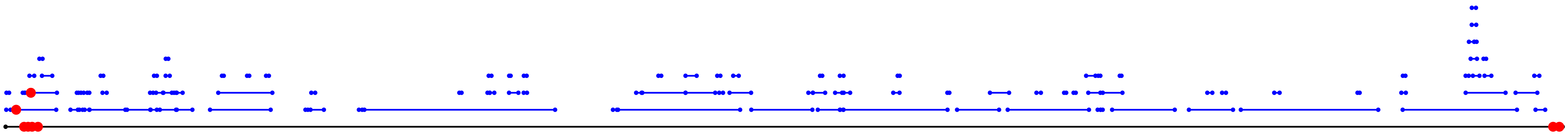

383_1 Chrm 11

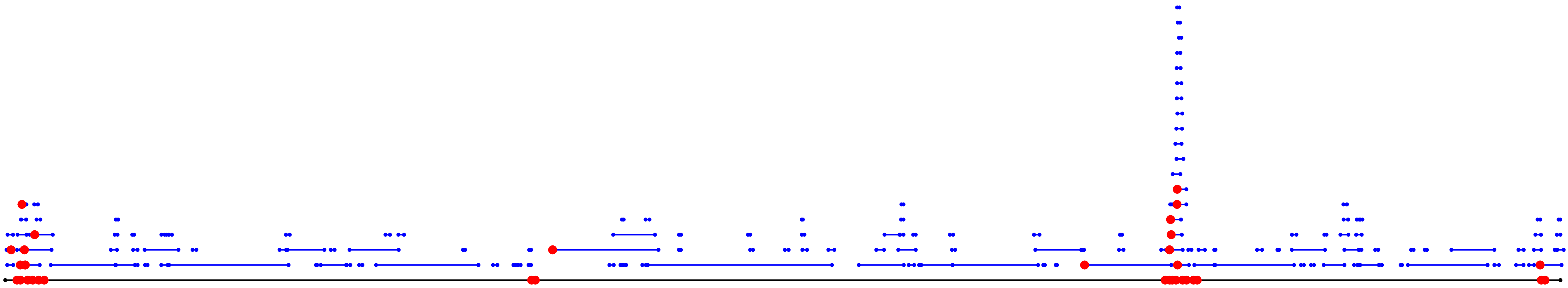

383_1 Chrm 12

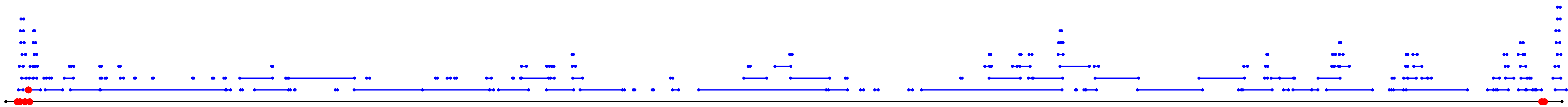

383_1 Chrm 13

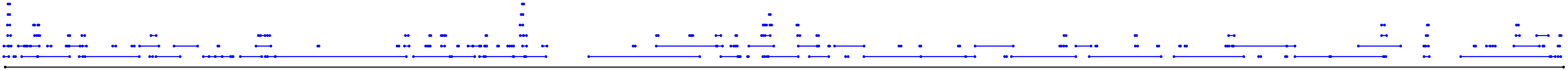

383_1 Chrm 14

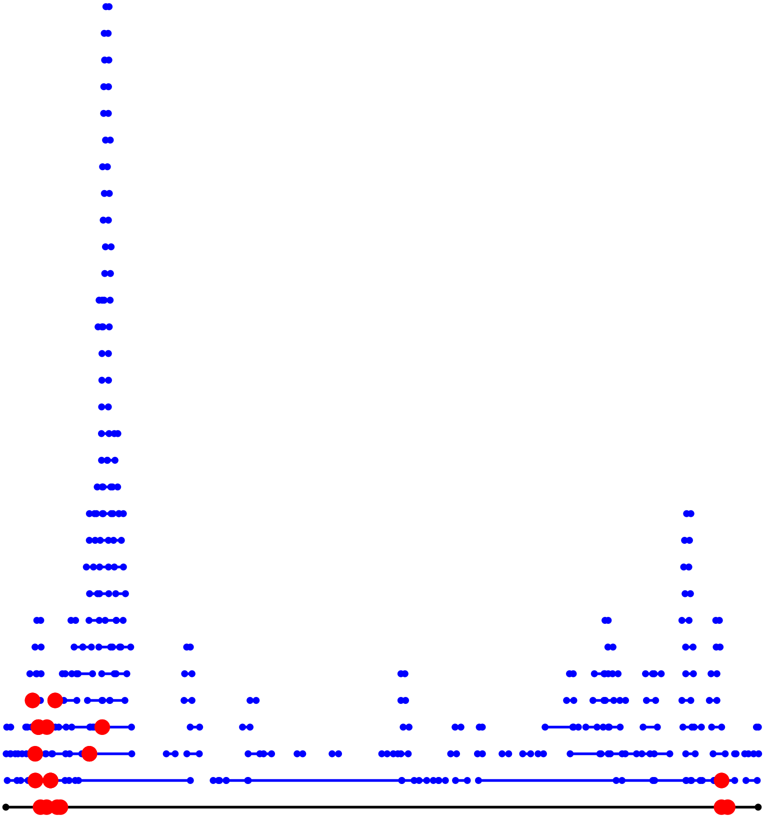

397_1 Chrm 1

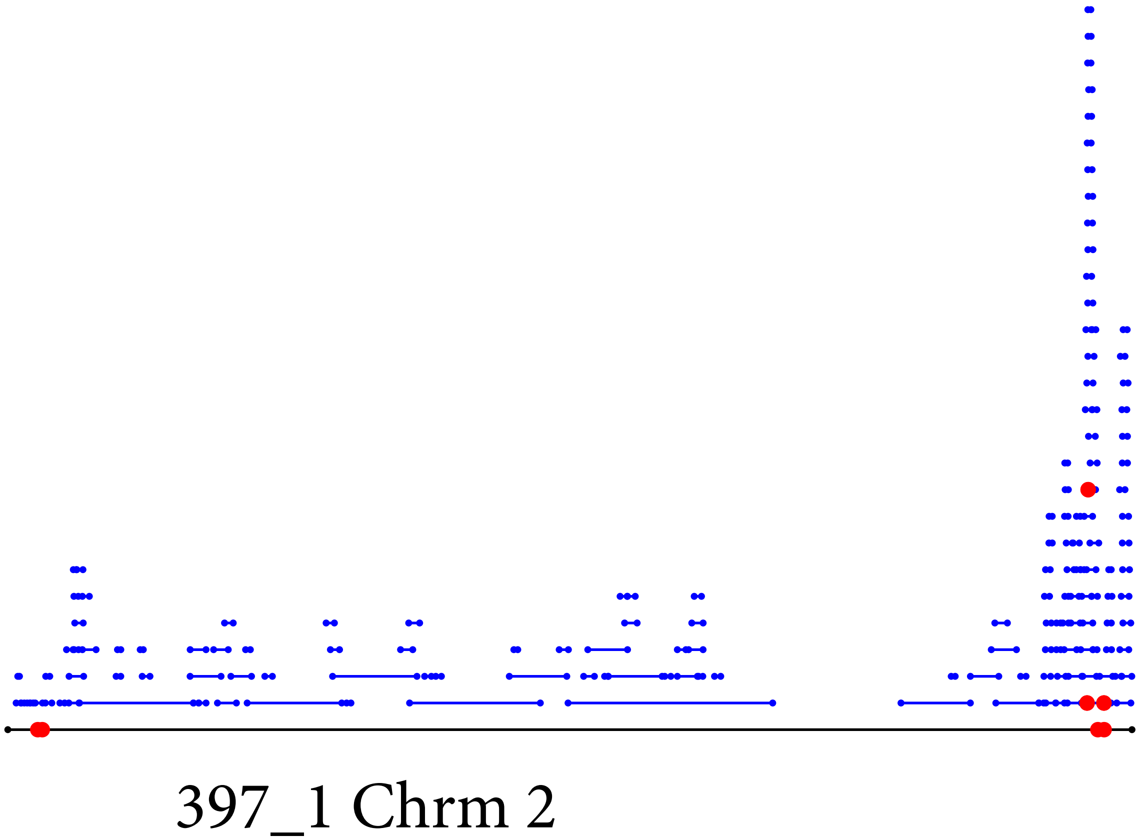
397_1 Chrm 2

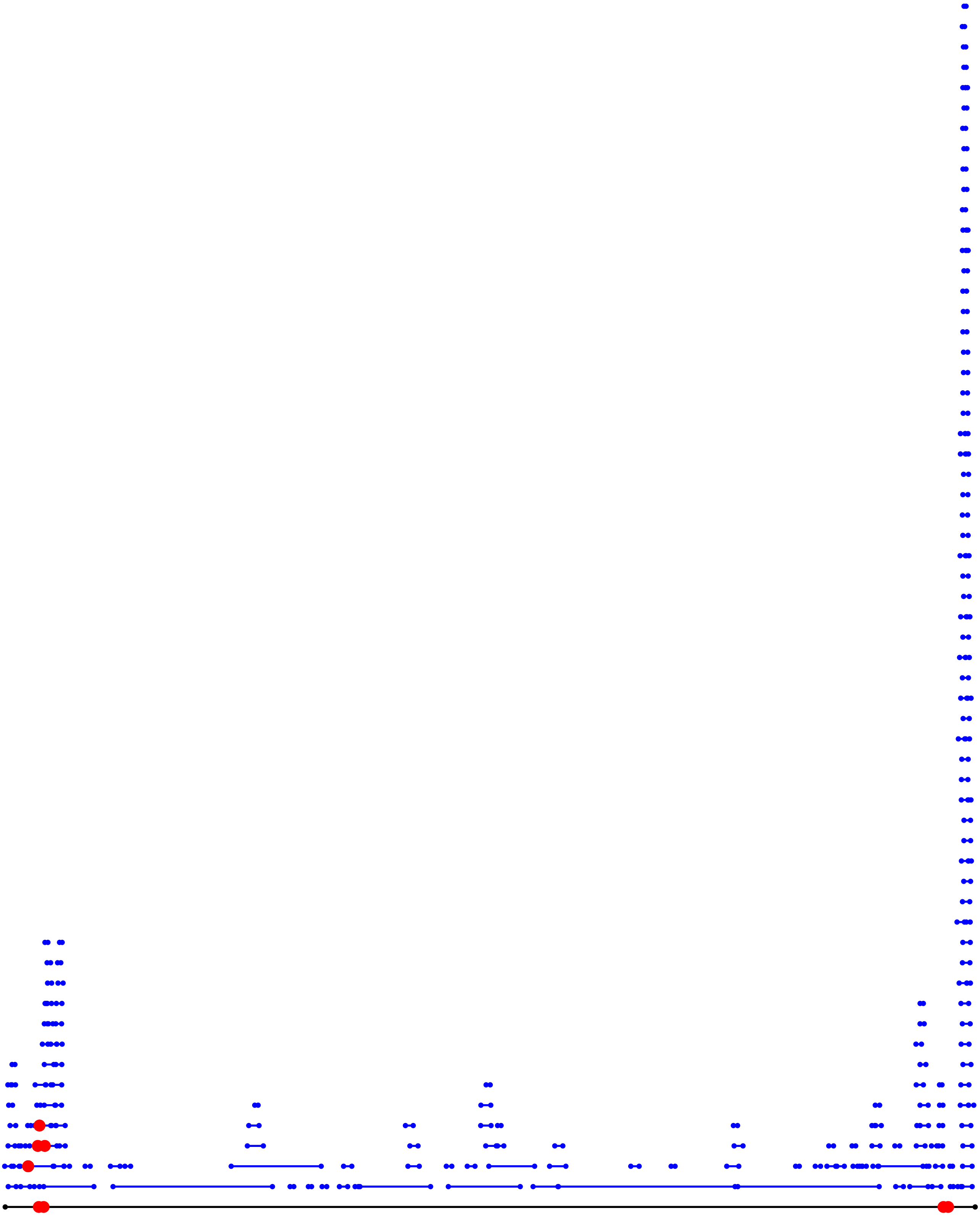

397_1 Chrm 3

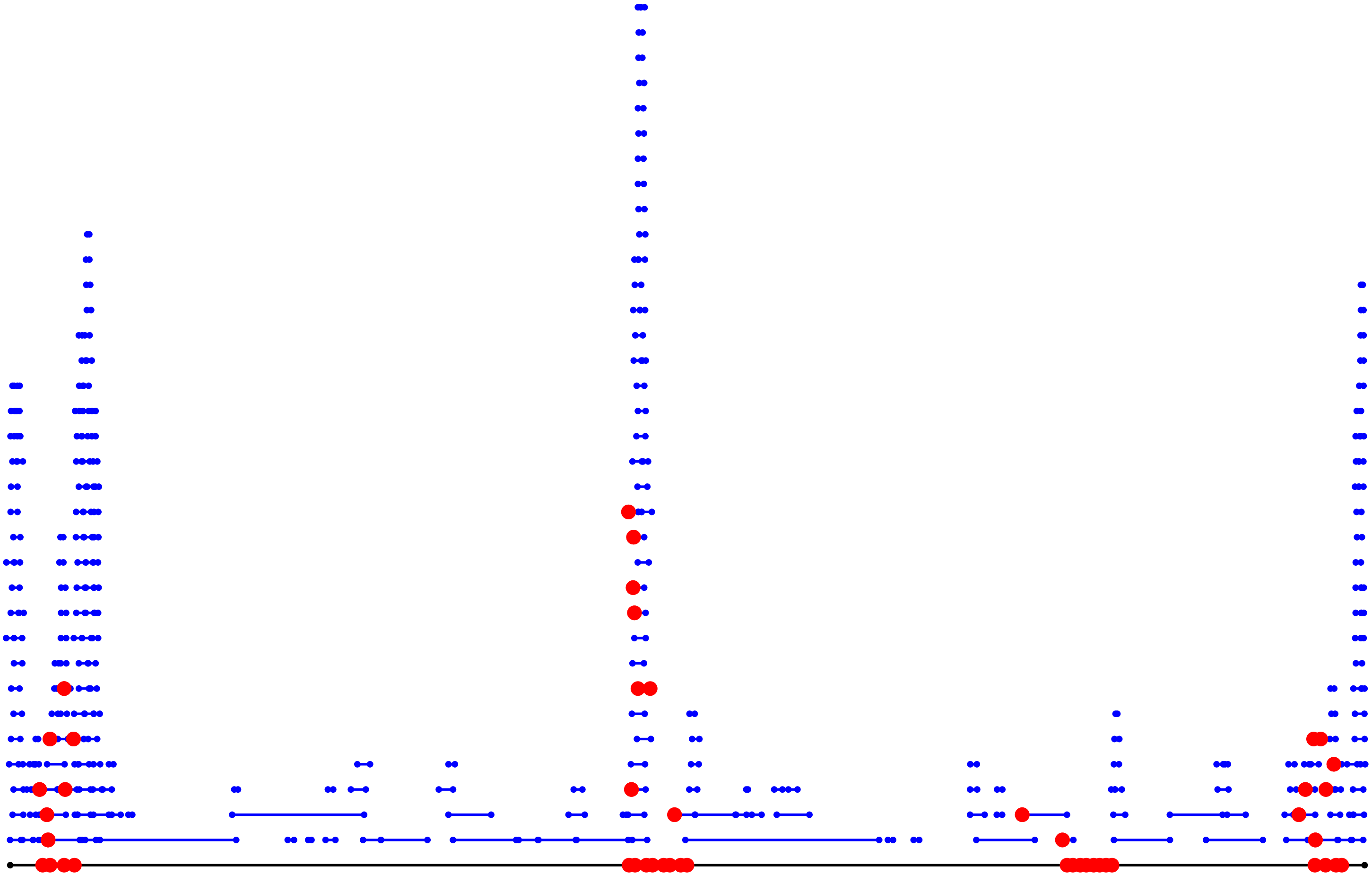

397_1 Chrm 4

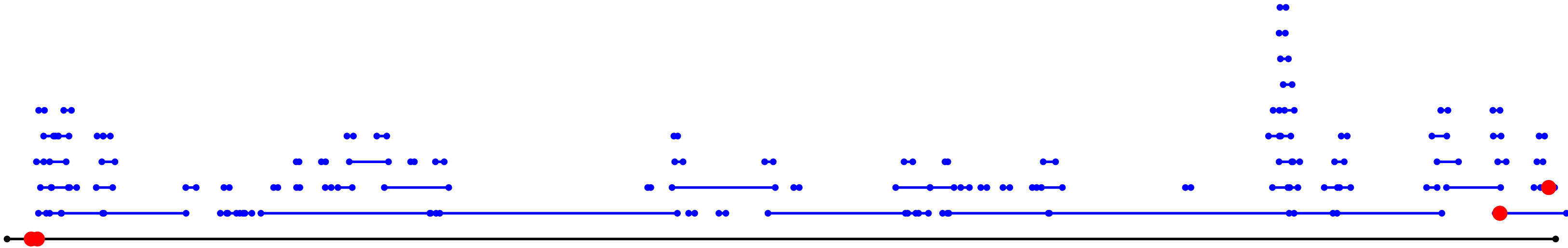

397_1 Chrm 5

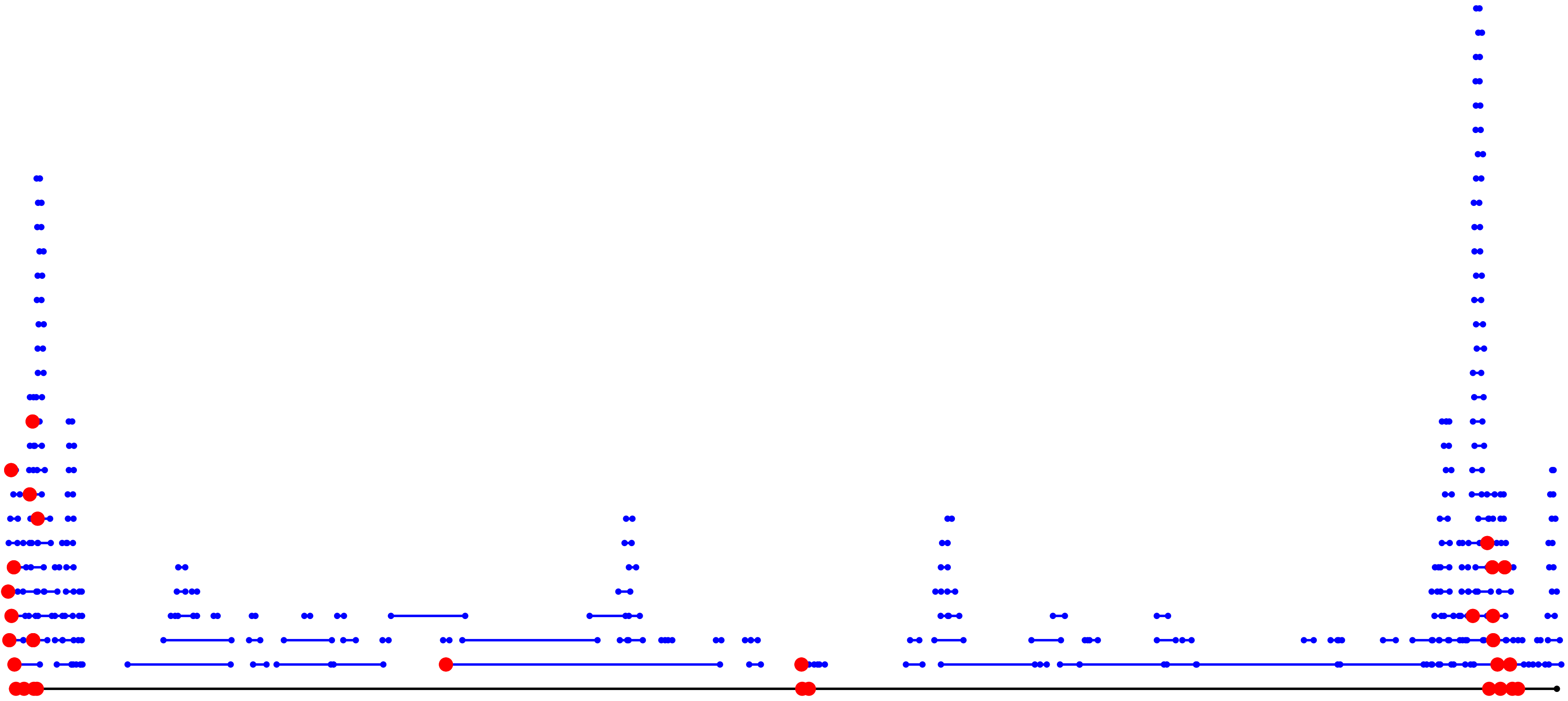

397_1 Chrm 6

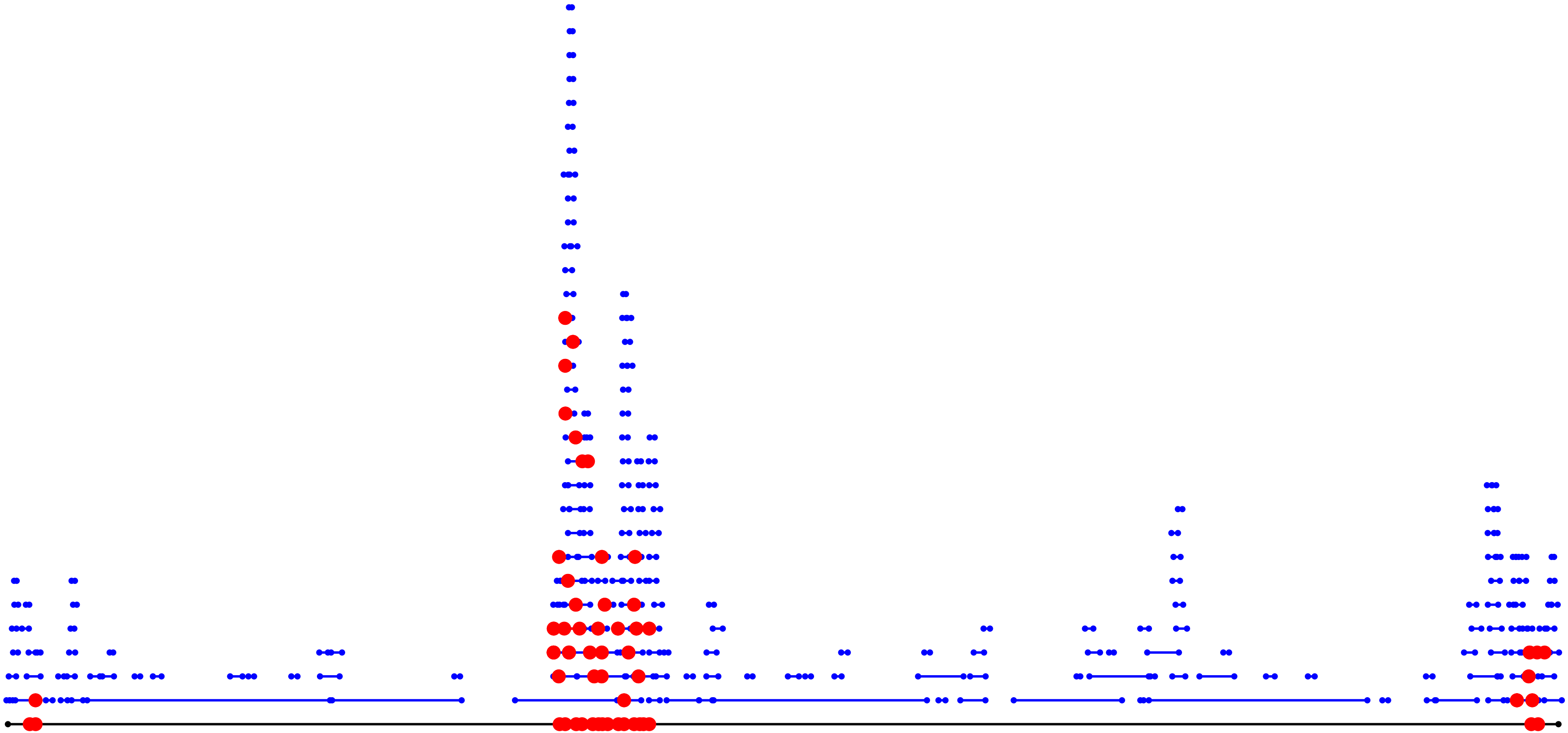

397_1 Chrm 7

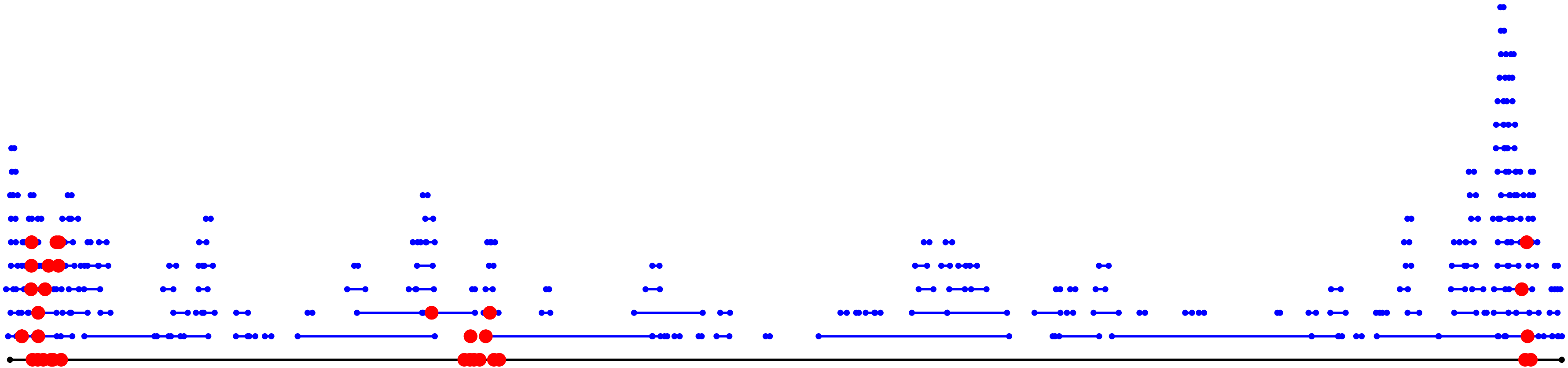

397_1 Chrm 8

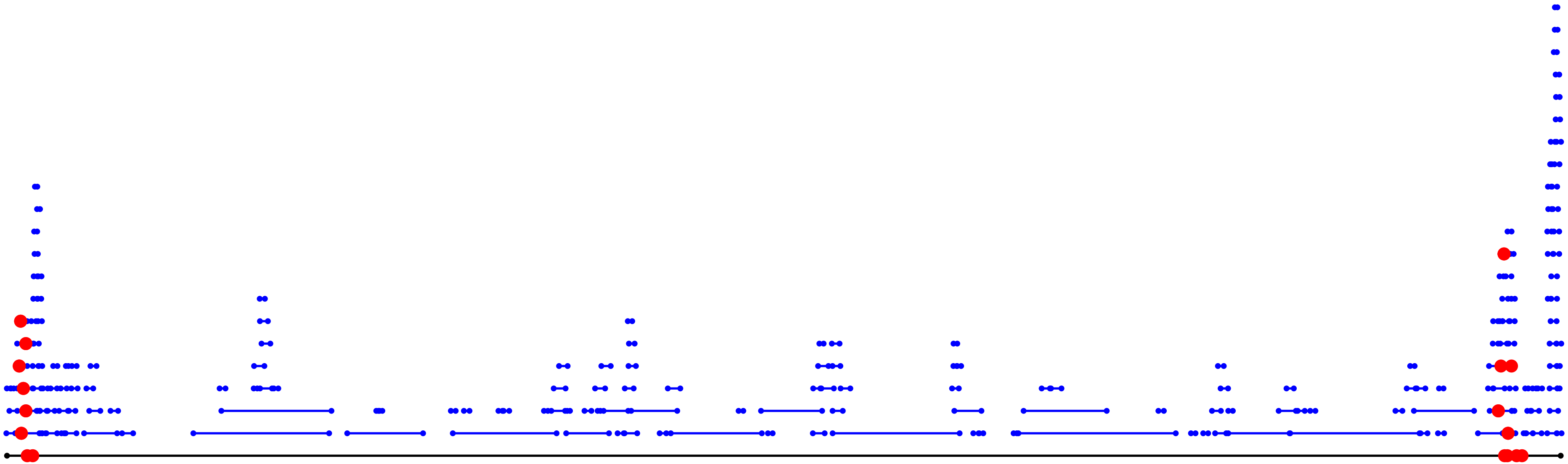

397_1 Chrm 9

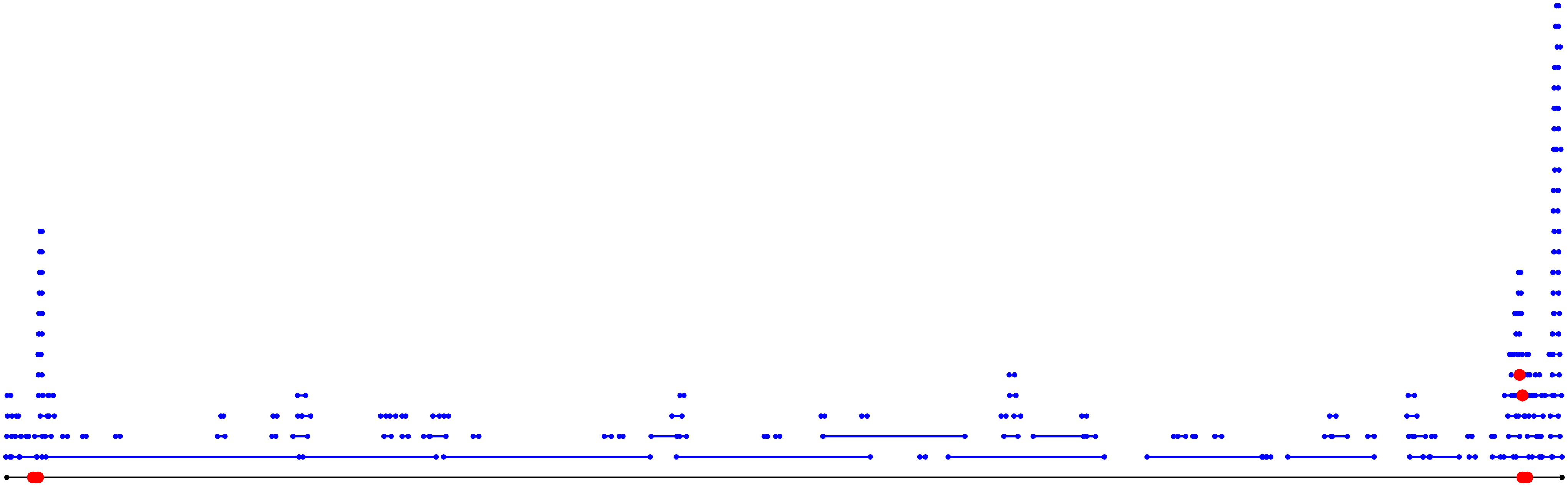

397_1 Chrm 10

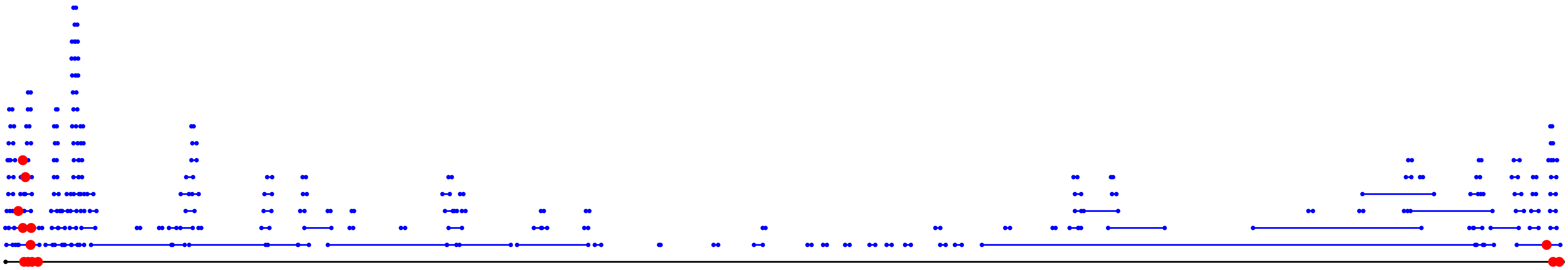

397_1 Chrm 11

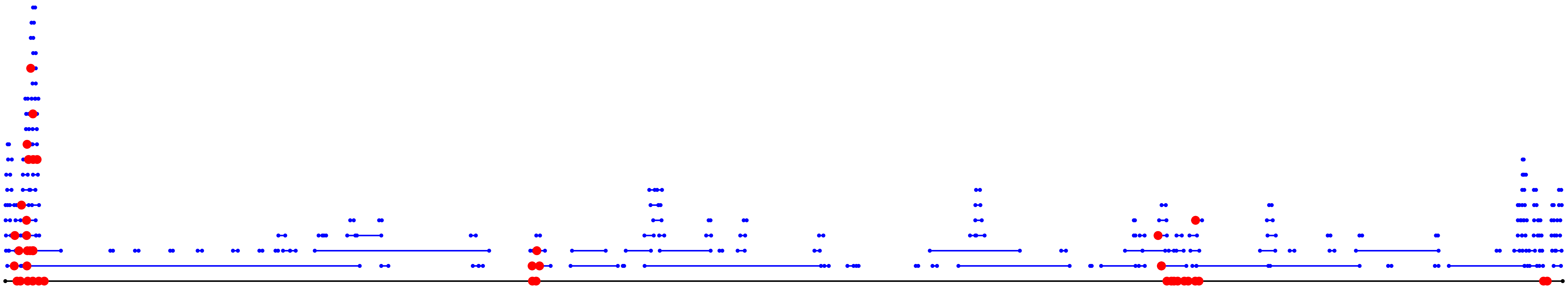

397_1 Chrm 12

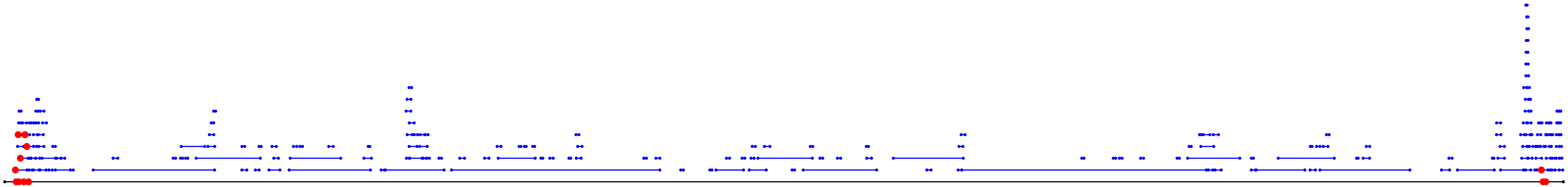

397_1 Chrm 13

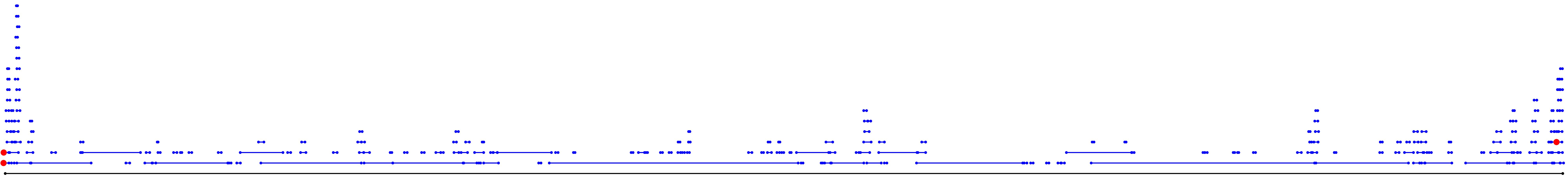

397_1 Chrm 14

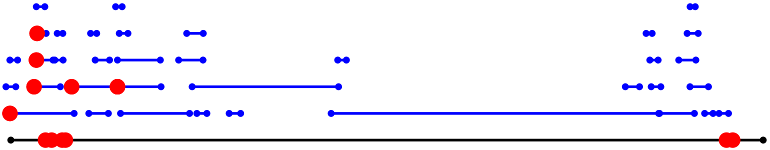

398_1 Chrm 1

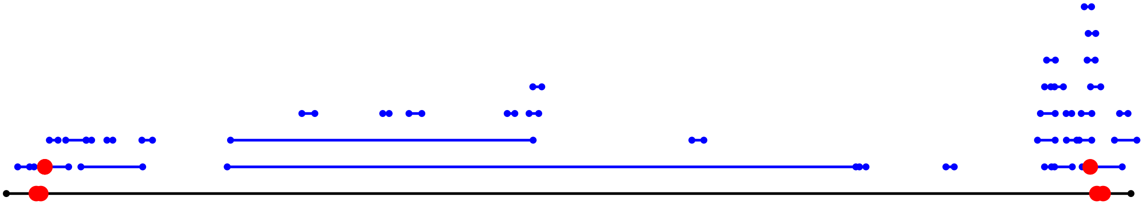

398_1 Chrm 2

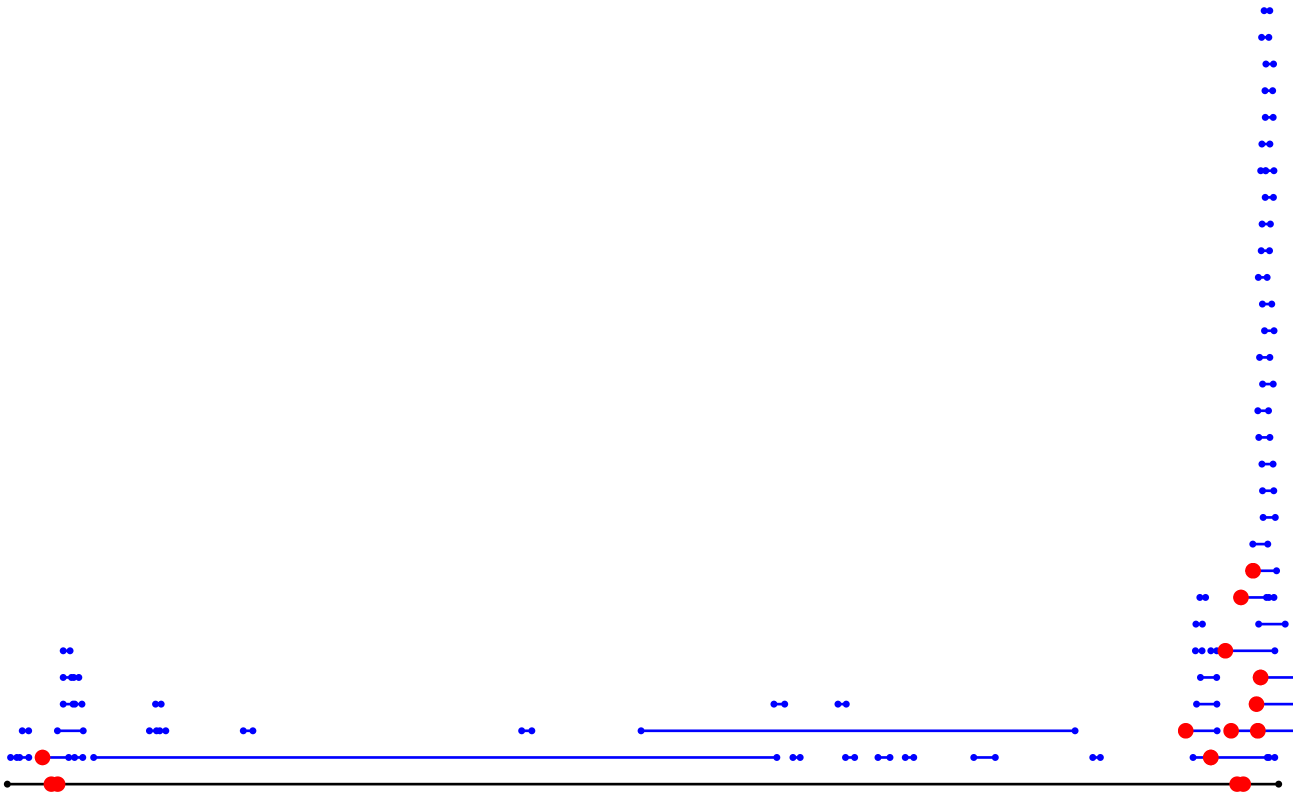

398_1 Chrm 3

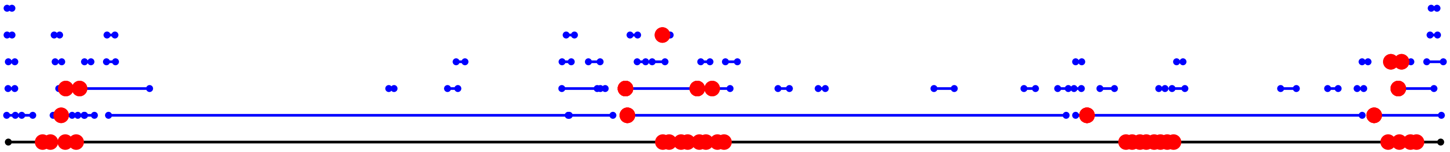

398_1 Chrm 4

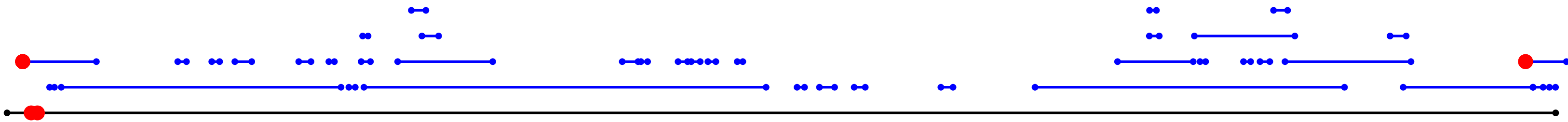

398_1 Chrm 5

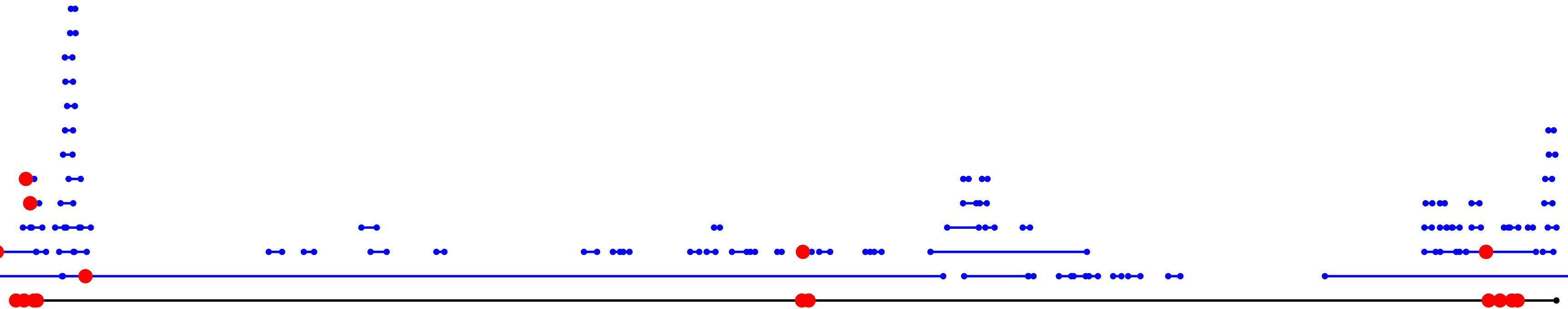

398_1 Chrm 6

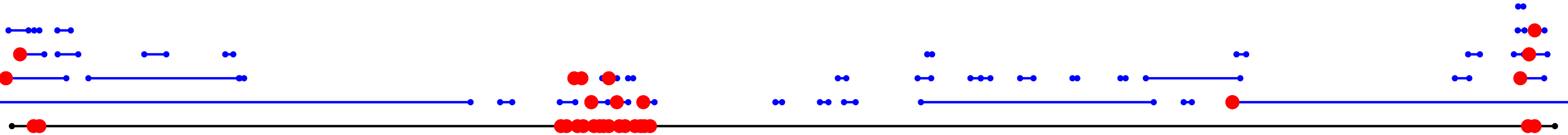

398_1 Chrm 7

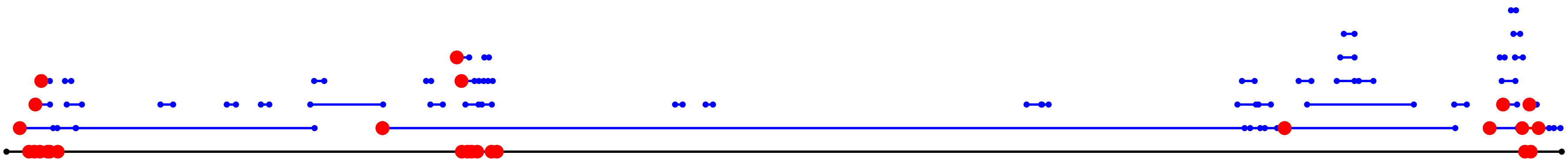

398_1 Chrm 8

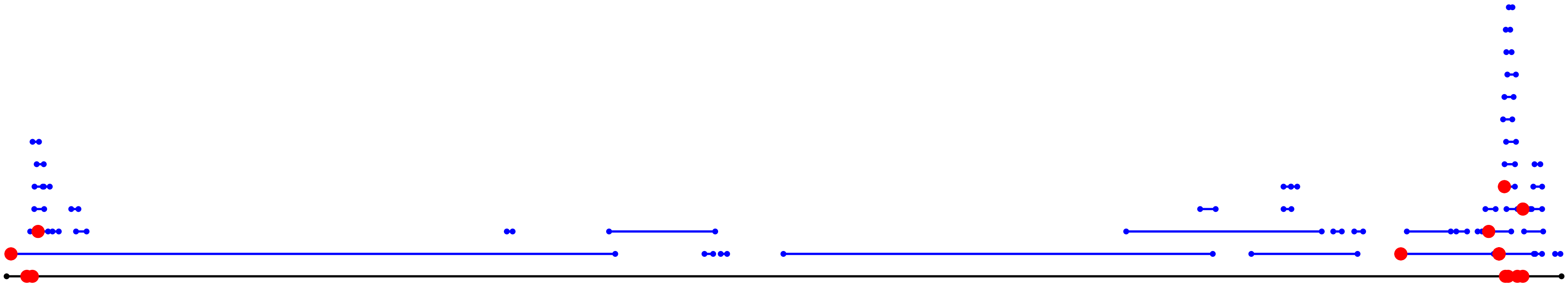

398_1 Chrm 9

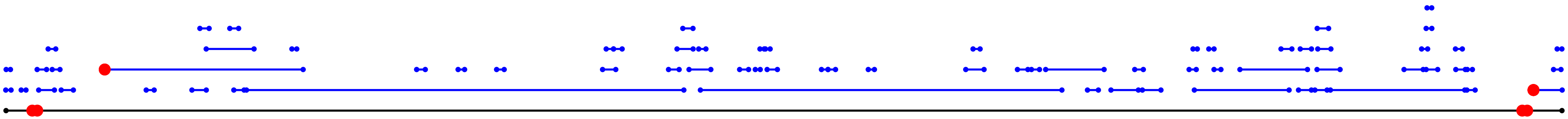

398_1 Chrm 10

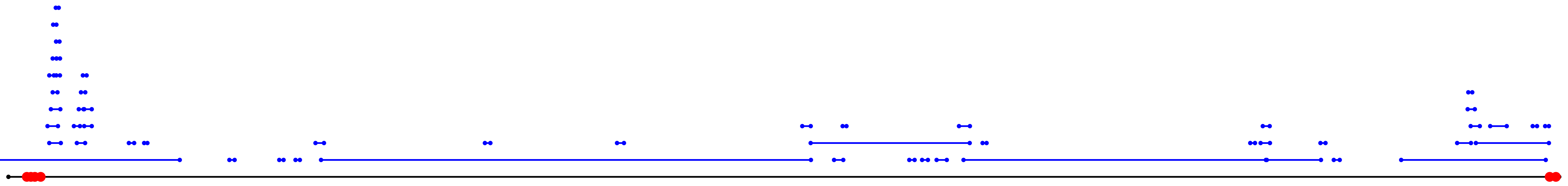

398_1 Chrm 11

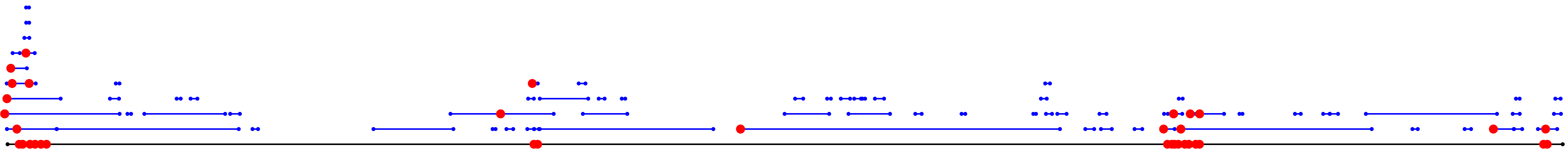

398_1 Chrm 12

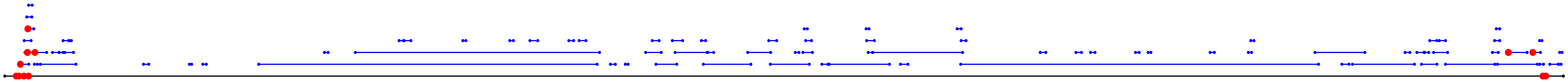

398_1 Chrm 13

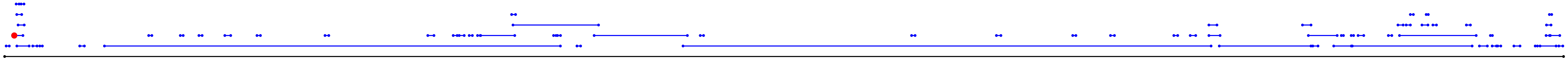

398_1 Chrm 14
