# Supplementary material for: New var reconstruction algorithm exposes high var sequence diversity in a single geographic location in Mali
Source: Genome Med. 2017 Mar 28;9:30. doi: 10.1186/s13073-017-0422-4 (PMC5368897; doi:10.1186/s13073-017-0422-4)
Supplement: Supplementary file 3 — A file containing Supplementary Figures S1–S5. Figure S1: Domain organization of PfEMP1 in each of 12 Malian isolates. Figure S2: Maximum likelihood phylogeny of DBLα domain sequences. Figure S3: Maximum likelihood phylogeny of CIDRα domain sequences. Figure S4: Detection and visualization of recombination events within and between var2csa sequences. Figure S5: Phylogenetic tree of reconstructed var2csa exon 1 sequences. (DOCX 3594 kb) [file 13073_2017_422_MOESM3_ESM.docx]

**Reconstruction of full-length *Plasmodium falciparum* *var* exon 1 sequences reveals severe malaria and pregnancy-associated malaria *var*s in uncomplicated malaria infections in Malian children.**

**Dara et al.**

**Additional File 3: Supplemental Figures**

|  | count |  |  |  |  |  |  |  |  |  |  |  |
| --- | --- | --- | --- | --- | --- | --- | --- | --- | --- | --- | --- | --- |
|  | **Isolate 303_1** | | | | | | | | | | | |
|  | 1 | CIDRa |  |  |  |  |  |  |  |  |  |  |
|  | 1 | CIDRa | DBLd |  |  |  |  |  |  |  |  |  |
|  | 3 | CIDRa | DBLd | CIDRb |  |  |  |  |  |  |  |  |
|  | 4 | CIDRb |  |  |  |  |  |  |  |  |  |  |
|  | 1 | CIDRb | DBLe |  |  |  |  |  |  |  |  |  |
|  | 1 | CIDRg |  |  |  |  |  |  |  |  |  |  |
|  | 1 | CIDRg | DBLe | DBLz | DBLe |  |  |  |  |  |  |  |
|  | 1 | DBLa | CIDRa | DBLb | DBLg |  |  |  |  |  |  |  |
|  | 1 | DBLa | CIDRa | DBLd |  |  |  |  |  |  |  |  |
|  | 1 | DBLa | CIDRa | DBLd | CIDRb |  |  |  |  |  |  |  |
|  | 1 | DBLb |  |  |  |  |  |  |  |  |  |  |
|  | 1 | DBLb | DBLb | DBLd | CIDRb |  |  |  |  |  |  |  |
|  | 1 | DBLb | DBLg | DBLe | DBLg | DBLz | DBLe |  |  |  |  |  |
|  | 3 | DBLd |  |  |  |  |  |  |  |  |  |  |
|  | 2 | DBLd | CIDRb |  |  |  |  |  |  |  |  |  |
|  | 1 | DBLd | CIDRb | DBLg |  |  |  |  |  |  |  |  |
|  | 1 | DBLe |  |  |  |  |  |  |  |  |  |  |
|  | 2 | DBLz | DBLe |  |  |  |  |  |  |  |  |  |
|  | 1 | NTS |  |  |  |  |  |  |  |  |  |  |
|  | 2 | NTS | DBLa |  |  |  |  |  |  |  |  |  |
|  | 7 | NTS | DBLa | CIDRa |  |  |  |  |  |  |  |  |
|  | 1 | NTS | DBLa | CIDRa | DBLb |  |  |  |  |  |  |  |
|  | 3 | NTS | DBLa | CIDRa | DBLb | DBLd | CIDRb |  |  |  |  |  |
|  | 1 | NTS | DBLa | CIDRa | DBLb | DBLd | CIDRg | DBLe | DBLe | DBLe |  |  |
|  | 2 | NTS | DBLa | CIDRa | DBLb | DBLg |  |  |  |  |  |  |
|  | 1 | NTS | DBLa | CIDRa | DBLb | DBLg | DBLd | CIDRb |  |  |  |  |
|  | 1 | NTS | DBLa | CIDRa | DBLb | DBLg | DBLd | CIDRb | DBLb | DBLg |  |  |
|  | 1 | NTS | DBLa | CIDRa | DBLb | DBLg | DBLd | CIDRd |  |  |  |  |
|  | 1 | NTS | DBLa | CIDRa | DBLb | DBLg | DBLg | DBLd | CIDRb |  |  |  |
|  | 2 | NTS | DBLa | CIDRa | DBLb | DBLg | DBLz | DBLe |  |  |  |  |
|  | 2 | NTS | DBLa | CIDRa | DBLd |  |  |  |  |  |  |  |
|  | 15 | NTS | DBLa | CIDRa | DBLd | CIDRb |  |  |  |  |  |  |
|  | 6 | NTS | DBLa | CIDRa | DBLd | CIDRg |  |  |  |  |  |  |
|  | 1 | NTS | DBLa | CIDRa | DBLd | CIDRg | DBLz | DBLe |  |  |  |  |
|  | 1 | NTS | DBLa | CIDRa | DBLg | DBLd | CIDRg | DBLe | DBLz | DBLe |  |  |
|  | 1 | NTS | DBLa | CIDRb |  |  |  |  |  |  |  |  |
|  | 1 | NTS | DBLa | CIDRd | DBLb | DBLg | DBLe | DBLg | DBLg | DBLg |  |  |
|  | 1 | NTS | DBLa | DBLd | CIDRb |  |  |  |  |  |  |  |
|  | 1 | NTS | DBLa | DBLg |  |  |  |  |  |  |  |  |
|  | 1 | NTSpam | DBLpam1 | DBLpam2 | CIDRpam | DBLpam3 | DBLe | DBLe | DBLe |  |  |  |
|  |  |  |  |  |  |  |  |  |  |  |  |  |
|  | **Isolate 309_1** | | | | | | | | | | | |
|  | 2 | CIDRa |  |  |  |  |  |  |  |  |  |  |
|  | 1 | CIDRa | DBLb | DBLg | DBLd | CIDRb |  |  |  |  |  |  |
|  | 1 | CIDRa | DBLd |  |  |  |  |  |  |  |  |  |
|  | 4 | CIDRa | DBLd | CIDRb |  |  |  |  |  |  |  |  |
|  | 1 | CIDRa | DBLd | CIDRg |  |  |  |  |  |  |  |  |
|  | 4 | CIDRb |  |  |  |  |  |  |  |  |  |  |
|  | 1 | DBLa | CIDRa |  |  |  |  |  |  |  |  |  |
|  | 1 | DBLa | CIDRa | DBLb | DBLb | DBLg | DBLg | DBLd | CIDRb |  |  |  |
|  | 1 | DBLa | CIDRa | DBLb | DBLd | CIDRb |  |  |  |  |  |  |
|  | 1 | DBLa | CIDRa | DBLb | DBLg | DBLg | DBLg |  |  |  |  |  |
|  | 2 | DBLa | CIDRa | DBLd | CIDRb |  |  |  |  |  |  |  |
|  | 3 | DBLb |  |  |  |  |  |  |  |  |  |  |
|  | 1 | DBLb | DBLb | DBLb | DBLd | CIDRb |  |  |  |  |  |  |
|  | 1 | DBLb | DBLd | CIDRb |  |  |  |  |  |  |  |  |
|  | 1 | DBLb | DBLd | CIDRg |  |  |  |  |  |  |  |  |
|  | 1 | DBLb | DBLg |  |  |  |  |  |  |  |  |  |
|  | 4 | DBLd | CIDRb |  |  |  |  |  |  |  |  |  |
|  | 1 | DBLd | CIDRb | DBLb |  |  |  |  |  |  |  |  |
|  | 1 | DBLd | CIDRb | DBLe | DBLe | DBLe |  |  |  |  |  |  |
|  | 2 | DBLd | CIDRg |  |  |  |  |  |  |  |  |  |
|  | 1 | DBLg | DBLz | DBLe |  |  |  |  |  |  |  |  |
|  | 3 | NTS | DBLa |  |  |  |  |  |  |  |  |  |
|  | 8 | NTS | DBLa | CIDRa |  |  |  |  |  |  |  |  |
|  | 2 | NTS | DBLa | CIDRa | DBLb |  |  |  |  |  |  |  |
|  | 1 | NTS | DBLa | CIDRa | DBLb | DBLb |  |  |  |  |  |  |
|  | 2 | NTS | DBLa | CIDRa | DBLb | DBLb | DBLg |  |  |  |  |  |
|  | 1 | NTS | DBLa | CIDRa | DBLb | DBLd |  |  |  |  |  |  |
|  | 3 | NTS | DBLa | CIDRa | DBLb | DBLd | CIDRb |  |  |  |  |  |
|  | 1 | NTS | DBLa | CIDRa | DBLb | DBLd | CIDRb | DBLg | DBLz |  |  |  |
|  | 1 | NTS | DBLa | CIDRa | DBLb | DBLg | DBLd | CIDRb |  |  |  |  |
|  | 2 | NTS | DBLa | CIDRa | DBLb | DBLg | DBLd | CIDRg |  |  |  |  |
|  | 3 | NTS | DBLa | CIDRa | DBLb | DBLg | DBLz | DBLe |  |  |  |  |
|  | 2 | NTS | DBLa | CIDRa | DBLd |  |  |  |  |  |  |  |
|  | 13 | NTS | DBLa | CIDRa | DBLd | CIDRb |  |  |  |  |  |  |
|  | 1 | NTS | DBLa | CIDRa | DBLd | CIDRg | DBLz | DBLe |  |  |  |  |
|  | 1 | NTS | DBLa | CIDRa | DBLg | DBLd | CIDRb | DBLe | DBLz |  |  |  |
|  | 1 | NTS | DBLa | CIDRa | DBLg | DBLd | CIDRg | DBLz | DBLe |  |  |  |
|  | 1 | NTS | DBLa | CIDRb | DBLg | DBLe | DBLz | DBLe |  |  |  |  |
|  | 1 | NTS | DBLa | CIDRd |  |  |  |  |  |  |  |  |
|  | 1 | NTS | DBLa | CIDRd | DBLg | DBLd | CIDRb | DBLb |  |  |  |  |
|  | 1 | NTS | DBLa | CIDRg | DBLb |  |  |  |  |  |  |  |
|  | 1 | NTSpam | DBLpam1 | DBLpam2 | CIDRpam | DBLpam3 | DBLe | DBLe |  |  |  |  |
|  | 1 | NTSpam | DBLpam1 | DBLpam2 | CIDRpam | DBLpam3 | DBLe | DBLe | DBLe |  |  |  |
|  |  |  |  |  |  |  |  |  |  |  |  |  |
|  | **Isolate 318_1** | | | | | | | | | | | |
|  | 1 | CIDRa | DBLb | DBLd | CIDRb |  |  |  |  |  |  |  |
|  | 1 | CIDRa | DBLb | DBLg | DBLe | DBLg | DBLz | DBLe |  |  |  |  |
|  | 1 | CIDRa | DBLd | CIDRb |  |  |  |  |  |  |  |  |
|  | 1 | CIDRa | DBLd | CIDRg |  |  |  |  |  |  |  |  |
|  | 1 | CIDRb |  |  |  |  |  |  |  |  |  |  |
|  | 1 | CIDRd | DBLb | DBLg | DBLz | DBLe |  |  |  |  |  |  |
|  | 1 | DBLb |  |  |  |  |  |  |  |  |  |  |
|  | 1 | DBLd |  |  |  |  |  |  |  |  |  |  |
|  | 1 | DBLd | CIDRb |  |  |  |  |  |  |  |  |  |
|  | 1 | DBLd | CIDRg | DBLe |  |  |  |  |  |  |  |  |
|  | 1 | DBLd | CIDRg | DBLe | DBLz | DBLe |  |  |  |  |  |  |
|  | 1 | DBLe |  |  |  |  |  |  |  |  |  |  |
|  | 1 | DBLg |  |  |  |  |  |  |  |  |  |  |
|  | 1 | NTS | DBLa | CIDRa |  |  |  |  |  |  |  |  |
|  | 1 | NTS | DBLa | CIDRa | DBLb | DBLd |  |  |  |  |  |  |
|  | 1 | NTS | DBLa | CIDRa | DBLb | DBLd | CIDRb |  |  |  |  |  |
|  | 1 | NTS | DBLa | CIDRa | DBLb | DBLg |  |  |  |  |  |  |
|  | 2 | NTS | DBLa | CIDRa | DBLb | DBLg | DBLd | CIDRg |  |  |  |  |
|  | 2 | NTS | DBLa | CIDRa | DBLd |  |  |  |  |  |  |  |
|  | 7 | NTS | DBLa | CIDRa | DBLd | CIDRb |  |  |  |  |  |  |
|  | 2 | NTS | DBLa | CIDRa | DBLd | CIDRb | DBLg |  |  |  |  |  |
|  | 2 | NTS | DBLa | CIDRa | DBLd | CIDRb | DBLz | DBLe |  |  |  |  |
|  | 7 | NTS | DBLa | CIDRa | DBLd | CIDRg |  |  |  |  |  |  |
|  | 1 | NTS | DBLa | CIDRa | DBLd | CIDRg | DBLe |  |  |  |  |  |
|  | 1 | NTS | DBLa | CIDRa | DBLd | CIDRg | DBLe | DBLe | DBLe |  |  |  |
|  | 1 | NTS | DBLa | CIDRa | DBLd | CIDRg | DBLe | DBLz | DBLe |  |  |  |
|  | 1 | NTS | DBLa | CIDRa | DBLd | CIDRg | DBLg |  |  |  |  |  |
|  | 2 | NTS | DBLa | CIDRa | DBLd | CIDRg | DBLz | DBLe |  |  |  |  |
|  | 1 | NTS | DBLa | CIDRa | DBLg | DBLz | DBLe |  |  |  |  |  |
|  | 1 | NTS | DBLa | CIDRb | DBLg | DBLd | CIDRg | DBLe | DBLe | DBLe |  |  |
|  | 1 | NTS | DBLa | CIDRd | DBLb | DBLg | DBLd | CIDRg |  |  |  |  |
|  | 1 | NTS | DBLa | CIDRd | DBLg | DBLd | CIDRg | DBLz | DBLe |  |  |  |
|  | 1 | NTS | DBLa | DBLd | CIDRb |  |  |  |  |  |  |  |
|  | 1 | NTS | DBLa | DBLd | CIDRg |  |  |  |  |  |  |  |
|  | 1 | NTS | DBLa | DBLe |  |  |  |  |  |  |  |  |
|  | 1 | NTSpam | DBLpam1 | DBLpam2 | CIDRpam | DBLpam3 | DBLe | DBLe | DBLe | DBLe |  |  |
|  | 1 | NTSpam | DBLpam1 | DBLpam2 | CIDRpam | DBLpam3 | DBLe | DBLe | DBLe | DBLe | DBLe |  |
|  |  |  |  |  |  |  |  |  |  |  |  |  |
|  | **Isolate 326_1** | | | | | | | | | | | |
|  | 1 | CIDRa |  |  |  |  |  |  |  |  |  |  |
|  | 1 | CIDRa | DBLb | DBLd | CIDRg |  |  |  |  |  |  |  |
|  | 1 | CIDRa | DBLb | DBLg | DBLd | CIDRb |  |  |  |  |  |  |
|  | 4 | CIDRa | DBLd | CIDRb |  |  |  |  |  |  |  |  |
|  | 1 | CIDRa | DBLd | CIDRg |  |  |  |  |  |  |  |  |
|  | 2 | CIDRb |  |  |  |  |  |  |  |  |  |  |
|  | 1 | CIDRb | DBLg | DBLe | DBLz | DBLe |  |  |  |  |  |  |
|  | 1 | CIDRg |  |  |  |  |  |  |  |  |  |  |
|  | 1 | CIDRg | DBLb | DBLg | DBLg | DBLz |  |  |  |  |  |  |
|  | 1 | CIDRg | DBLg | DBLd | CIDRb | DBLb |  |  |  |  |  |  |
|  | 2 | DBLa | CIDRa | DBLb |  |  |  |  |  |  |  |  |
|  | 1 | DBLa | CIDRa | DBLd | CIDRb |  |  |  |  |  |  |  |
|  | 1 | DBLb |  |  |  |  |  |  |  |  |  |  |
|  | 1 | DBLb | DBLd | CIDRb |  |  |  |  |  |  |  |  |
|  | 1 | DBLb | DBLd | CIDRg |  |  |  |  |  |  |  |  |
|  | 1 | DBLb | DBLd | CIDRg | DBLz | DBLe |  |  |  |  |  |  |
|  | 6 | DBLd | CIDRb |  |  |  |  |  |  |  |  |  |
|  | 1 | DBLd | CIDRg |  |  |  |  |  |  |  |  |  |
|  | 1 | DBLe |  |  |  |  |  |  |  |  |  |  |
|  | 1 | DBLe | DBLe | DBLe |  |  |  |  |  |  |  |  |
|  | 2 | NTS | DBLa |  |  |  |  |  |  |  |  |  |
|  | 4 | NTS | DBLa | CIDRa |  |  |  |  |  |  |  |  |
|  | 1 | NTS | DBLa | CIDRa | DBLb |  |  |  |  |  |  |  |
|  | 1 | NTS | DBLa | CIDRa | DBLb | DBLb |  |  |  |  |  |  |
|  | 1 | NTS | DBLa | CIDRa | DBLb | DBLb | DBLg | DBLd | CIDRb |  |  |  |
|  | 1 | NTS | DBLa | CIDRa | DBLb | DBLd | CIDRb |  |  |  |  |  |
|  | 1 | NTS | DBLa | CIDRa | DBLb | DBLd | CIDRg | DBLg | DBLz |  |  |  |
|  | 1 | NTS | DBLa | CIDRa | DBLb | DBLg |  |  |  |  |  |  |
|  | 1 | NTS | DBLa | CIDRa | DBLb | DBLg | DBLd | CIDRb |  |  |  |  |
|  | 1 | NTS | DBLa | CIDRa | DBLb | DBLg | DBLd | CIDRg |  |  |  |  |
|  | 1 | NTS | DBLa | CIDRa | DBLb | DBLg | DBLg | DBLd | CIDRb |  |  |  |
|  | 1 | NTS | DBLa | CIDRa | DBLb | DBLg | DBLg | DBLz | DBLe |  |  |  |
|  | 2 | NTS | DBLa | CIDRa | DBLb | DBLg | DBLz |  |  |  |  |  |
|  | 11 | NTS | DBLa | CIDRa | DBLd | CIDRb |  |  |  |  |  |  |
|  | 1 | NTS | DBLa | CIDRa | DBLd | CIDRb | DBLg | DBLz | DBLe |  |  |  |
|  | 3 | NTS | DBLa | CIDRa | DBLd | CIDRg |  |  |  |  |  |  |
|  | 1 | NTS | DBLa | CIDRa | DBLd | CIDRg | DBLe |  |  |  |  |  |
|  | 1 | NTS | DBLa | CIDRa | DBLd | CIDRg | DBLg |  |  |  |  |  |
|  | 1 | NTS | DBLa | CIDRa | DBLg | DBLd | CIDRb | DBLb | DBLg |  |  |  |
|  | 1 | NTS | DBLa | CIDRb | DBLg | DBLe | DBLz | DBLe |  |  |  |  |
|  | 1 | NTS | DBLa | CIDRd | DBLb | DBLg | DBLe | DBLd | CIDRb |  |  |  |
|  | 1 | NTS | DBLa | CIDRd | DBLb | DBLg | DBLg | DBLe | DBLg |  |  |  |
|  | 1 | NTS | DBLa | CIDRd | DBLb | DBLg | DBLz | DBLe |  |  |  |  |
|  | 1 | NTSpam | DBLpam1 | DBLpam2 | CIDRpam | DBLpam3 | DBLe | DBLe | DBLe |  |  |  |
|  |  |  |  |  |  |  |  |  |  |  |  |  |
|  | **Isolate 327_1** | | | | | | | | | | | |
|  | 1 | CIDRa | DBLd |  |  |  |  |  |  |  |  |  |
|  | 6 | CIDRa | DBLd | CIDRb |  |  |  |  |  |  |  |  |
|  | 1 | CIDRa | DBLd | CIDRg |  |  |  |  |  |  |  |  |
|  | 2 | CIDRb |  |  |  |  |  |  |  |  |  |  |
|  | 1 | CIDRg | DBLe | DBLz | DBLe |  |  |  |  |  |  |  |
|  | 1 | DBLb |  |  |  |  |  |  |  |  |  |  |
|  | 2 | DBLb | DBLb | DBLd | CIDRb |  |  |  |  |  |  |  |
|  | 1 | DBLb | DBLd | CIDRb |  |  |  |  |  |  |  |  |
|  | 3 | DBLd | CIDRb |  |  |  |  |  |  |  |  |  |
|  | 1 | DBLg |  |  |  |  |  |  |  |  |  |  |
|  | 1 | NTS |  |  |  |  |  |  |  |  |  |  |
|  | 2 | NTS | DBLa | CIDRa |  |  |  |  |  |  |  |  |
|  | 1 | NTS | DBLa | CIDRa | DBLb |  |  |  |  |  |  |  |
|  | 1 | NTS | DBLa | CIDRa | DBLb | DBLb | DBLg | DBLd | CIDRb |  |  |  |
|  | 1 | NTS | DBLa | CIDRa | DBLb | DBLb | DBLg | DBLg | DBLd | CIDRb |  |  |
|  | 4 | NTS | DBLa | CIDRa | DBLb | DBLd | CIDRb |  |  |  |  |  |
|  | 1 | NTS | DBLa | CIDRa | DBLb | DBLd | CIDRb | DBLg | DBLz | DBLe |  |  |
|  | 1 | NTS | DBLa | CIDRa | DBLb | DBLd | CIDRg |  |  |  |  |  |
|  | 2 | NTS | DBLa | CIDRa | DBLb | DBLg | DBLd | CIDRb | DBLb | DBLg |  |  |
|  | 1 | NTS | DBLa | CIDRa | DBLb | DBLg | DBLd | CIDRd |  |  |  |  |
|  | 1 | NTS | DBLa | CIDRa | DBLb | DBLg | DBLe | DBLg | DBLz | DBLe |  |  |
|  | 1 | NTS | DBLa | CIDRa | DBLb | DBLg | DBLg | DBLg |  |  |  |  |
|  | 1 | NTS | DBLa | CIDRa | DBLb | DBLg | DBLg | DBLg | DBLd | CIDRb |  |  |
|  | 1 | NTS | DBLa | CIDRa | DBLb | DBLg | DBLz |  |  |  |  |  |
|  | 2 | NTS | DBLa | CIDRa | DBLb | DBLg | DBLz | DBLe |  |  |  |  |
|  | 21 | NTS | DBLa | CIDRa | DBLd | CIDRb |  |  |  |  |  |  |
|  | 1 | NTS | DBLa | CIDRa | DBLd | CIDRb | DBLe | DBLe |  |  |  |  |
|  | 1 | NTS | DBLa | CIDRa | DBLd | CIDRb | DBLe | DBLz | DBLe |  |  |  |
|  | 4 | NTS | DBLa | CIDRa | DBLd | CIDRg |  |  |  |  |  |  |
|  | 1 | NTS | DBLa | CIDRa | DBLd | CIDRg | DBLe | DBLz | DBLe |  |  |  |
|  | 1 | NTS | DBLa | CIDRa | DBLd | CIDRg | DBLz | DBLe |  |  |  |  |
|  | 2 | NTS | DBLa | CIDRa | DBLg | DBLd | CIDRb |  |  |  |  |  |
|  | 1 | NTS | DBLa | CIDRa | DBLg | DBLd | CIDRb | DBLg |  |  |  |  |
|  | 1 | NTS | DBLa | CIDRa | DBLg | DBLd | CIDRg |  |  |  |  |  |
|  | 1 | NTS | DBLa | CIDRa | DBLg | DBLg | DBLz | DBLe |  |  |  |  |
|  | 1 | NTS | DBLa | CIDRb | DBLg | DBLe | DBLz | DBLe |  |  |  |  |
|  | 1 | NTS | DBLa | CIDRg | DBLb | DBLd | CIDRb |  |  |  |  |  |
|  | 1 | NTS | DBLa | DBLe |  |  |  |  |  |  |  |  |
|  | 1 | NTSpam | DBLpam1 | DBLpam2 | CIDRpam | DBLpam3 | DBLe | DBLe | DBLe |  |  |  |
|  |  |  |  |  |  |  |  |  |  |  |  |  |
|  | **Isolate 365_1** | | | | | | | | | | | |
|  | 3 | CIDRa |  |  |  |  |  |  |  |  |  |  |
|  | 1 | CIDRa | DBLb | DBLg |  |  |  |  |  |  |  |  |
|  | 1 | CIDRa | DBLb | DBLg | DBLd | CIDRb |  |  |  |  |  |  |
|  | 2 | CIDRa | DBLd | CIDRb |  |  |  |  |  |  |  |  |
|  | 4 | CIDRa | DBLd | CIDRg |  |  |  |  |  |  |  |  |
|  | 6 | CIDRb |  |  |  |  |  |  |  |  |  |  |
|  | 1 | CIDRb | DBLb | DBLg | DBLe |  |  |  |  |  |  |  |
|  | 1 | CIDRb | DBLe | DBLe | DBLe |  |  |  |  |  |  |  |
|  | 1 | CIDRd | DBLb |  |  |  |  |  |  |  |  |  |
|  | 1 | CIDRd | DBLb | DBLg |  |  |  |  |  |  |  |  |
|  | 3 | CIDRg |  |  |  |  |  |  |  |  |  |  |
|  | 1 | CIDRg | DBLg | DBLz | DBLe |  |  |  |  |  |  |  |
|  | 2 | DBLa | CIDRa |  |  |  |  |  |  |  |  |  |
|  | 1 | DBLa | CIDRa | DBLd | CIDRg |  |  |  |  |  |  |  |
|  | 2 | DBLb |  |  |  |  |  |  |  |  |  |  |
|  | 1 | DBLb | DBLb |  |  |  |  |  |  |  |  |  |
|  | 1 | DBLb | DBLb | DBLg |  |  |  |  |  |  |  |  |
|  | 1 | DBLb | DBLd | CIDRb |  |  |  |  |  |  |  |  |
|  | 2 | DBLd |  |  |  |  |  |  |  |  |  |  |
|  | 3 | DBLd | CIDRb |  |  |  |  |  |  |  |  |  |
|  | 1 | DBLd | CIDRb | DBLg | DBLz |  |  |  |  |  |  |  |
|  | 1 | DBLd | CIDRb | DBLg | DBLz | DBLe |  |  |  |  |  |  |
|  | 2 | DBLd | CIDRg |  |  |  |  |  |  |  |  |  |
|  | 1 | DBLe | DBLe |  |  |  |  |  |  |  |  |  |
|  | 1 | DBLe | DBLe | DBLe |  |  |  |  |  |  |  |  |
|  | 1 | DBLg | DBLd | CIDRb | DBLb |  |  |  |  |  |  |  |
|  | 1 | DBLg | DBLd | CIDRg |  |  |  |  |  |  |  |  |
|  | 1 | DBLg | DBLz |  |  |  |  |  |  |  |  |  |
|  | 2 | DBLz | DBLe |  |  |  |  |  |  |  |  |  |
|  | 2 | NTS |  |  |  |  |  |  |  |  |  |  |
|  | 4 | NTS | DBLa |  |  |  |  |  |  |  |  |  |
|  | 4 | NTS | DBLa | CIDRa |  |  |  |  |  |  |  |  |
|  | 3 | NTS | DBLa | CIDRa | DBLb |  |  |  |  |  |  |  |
|  | 1 | NTS | DBLa | CIDRa | DBLb | DBLd | CIDRb |  |  |  |  |  |
|  | 1 | NTS | DBLa | CIDRa | DBLb | DBLd | CIDRb | DBLg | DBLe | DBLe |  |  |
|  | 1 | NTS | DBLa | CIDRa | DBLb | DBLg | DBLd | CIDRb |  |  |  |  |
|  | 1 | NTS | DBLa | CIDRa | DBLb | DBLg | DBLz |  |  |  |  |  |
|  | 2 | NTS | DBLa | CIDRa | DBLd |  |  |  |  |  |  |  |
|  | 4 | NTS | DBLa | CIDRa | DBLd | CIDRb |  |  |  |  |  |  |
|  | 4 | NTS | DBLa | CIDRa | DBLd | CIDRg |  |  |  |  |  |  |
|  | 1 | NTS | DBLa | CIDRa | DBLe |  |  |  |  |  |  |  |
|  | 1 | NTS | DBLa | CIDRa | DBLg |  |  |  |  |  |  |  |
|  | 1 | NTS | DBLa | CIDRa | DBLg | DBLd |  |  |  |  |  |  |
|  | 1 | NTS | DBLa | CIDRa | DBLg | DBLz | DBLe | DBLe |  |  |  |  |
|  | 1 | NTS | DBLa | CIDRd |  |  |  |  |  |  |  |  |
|  | 1 | NTS | DBLa | CIDRd | DBLb | DBLg | DBLz | DBLe |  |  |  |  |
|  | 1 | NTS | DBLa | CIDRg | DBLb | DBLg | DBLz | DBLe |  |  |  |  |
|  | 2 | NTS | DBLa | DBLe |  |  |  |  |  |  |  |  |
|  | 1 | NTSpam | DBLpam1 | DBLpam2 | CIDRpam | DBLpam3 | DBLe | DBLe | DBLe |  |  |  |
|  |  |  |  |  |  |  |  |  |  |  |  |  |
|  | **Isolate 366_1** | | | | | | | | | | | |
|  | 1 | CIDRa |  |  |  |  |  |  |  |  |  |  |
|  | 1 | CIDRa | DBLb | DBLd | CIDRb |  |  |  |  |  |  |  |
|  | 1 | CIDRa | DBLb | DBLg | DBLe | DBLe |  |  |  |  |  |  |
|  | 1 | CIDRa | DBLb | DBLz | DBLe |  |  |  |  |  |  |  |
|  | 2 | CIDRa | DBLd | CIDRb |  |  |  |  |  |  |  |  |
|  | 1 | CIDRa | DBLd | CIDRb | DBLe |  |  |  |  |  |  |  |
|  | 2 | CIDRa | DBLd | CIDRg |  |  |  |  |  |  |  |  |
|  | 1 | CIDRa | DBLd | CIDRg | DBLe |  |  |  |  |  |  |  |
|  | 1 | CIDRa | DBLg | DBLd | CIDRg | DBLz | DBLe |  |  |  |  |  |
|  | 1 | CIDRb |  |  |  |  |  |  |  |  |  |  |
|  | 1 | CIDRg | DBLz | DBLe |  |  |  |  |  |  |  |  |
|  | 1 | DBLa | CIDRa | DBLd | CIDRb |  |  |  |  |  |  |  |
|  | 1 | DBLa | CIDRg | DBLg | DBLd | CIDRb | DBLb |  |  |  |  |  |
|  | 1 | DBLb |  |  |  |  |  |  |  |  |  |  |
|  | 1 | DBLb | DBLb |  |  |  |  |  |  |  |  |  |
|  | 2 | DBLb | DBLb | DBLd | CIDRg |  |  |  |  |  |  |  |
|  | 1 | DBLb | DBLb | DBLg | DBLd | CIDRg |  |  |  |  |  |  |
|  | 2 | DBLb | DBLd | CIDRb |  |  |  |  |  |  |  |  |
|  | 1 | DBLb | DBLd | CIDRb | DBLb | DBLg |  |  |  |  |  |  |
|  | 3 | DBLd | CIDRb |  |  |  |  |  |  |  |  |  |
|  | 1 | DBLd | CIDRg |  |  |  |  |  |  |  |  |  |
|  | 1 | DBLg |  |  |  |  |  |  |  |  |  |  |
|  | 1 | DBLg | DBLz | DBLe |  |  |  |  |  |  |  |  |
|  | 1 | DBLz | DBLe |  |  |  |  |  |  |  |  |  |
|  | 1 | NTS |  |  |  |  |  |  |  |  |  |  |
|  | 3 | NTS | DBLa |  |  |  |  |  |  |  |  |  |
|  | 2 | NTS | DBLa | CIDRa | DBLb |  |  |  |  |  |  |  |
|  | 1 | NTS | DBLa | CIDRa | DBLb | DBLb | DBLd | CIDRb |  |  |  |  |
|  | 1 | NTS | DBLa | CIDRa | DBLb | DBLd | CIDRb |  |  |  |  |  |
|  | 1 | NTS | DBLa | CIDRa | DBLb | DBLd | CIDRg | DBLe | DBLe |  |  |  |
|  | 1 | NTS | DBLa | CIDRa | DBLb | DBLg |  |  |  |  |  |  |
|  | 1 | NTS | DBLa | CIDRa | DBLb | DBLg | DBLd |  |  |  |  |  |
|  | 2 | NTS | DBLa | CIDRa | DBLb | DBLg | DBLd | CIDRb |  |  |  |  |
|  | 1 | NTS | DBLa | CIDRa | DBLb | DBLg | DBLd | CIDRg |  |  |  |  |
|  | 1 | NTS | DBLa | CIDRa | DBLb | DBLg | DBLd | CIDRg | DBLz | DBLe |  |  |
|  | 1 | NTS | DBLa | CIDRa | DBLb | DBLg | DBLe | DBLg | DBLz | DBLe |  |  |
|  | 1 | NTS | DBLa | CIDRa | DBLb | DBLg | DBLg |  |  |  |  |  |
|  | 1 | NTS | DBLa | CIDRa | DBLb | DBLg | DBLg | DBLg |  |  |  |  |
|  | 1 | NTS | DBLa | CIDRa | DBLb | DBLg | DBLz | DBLe |  |  |  |  |
|  | 3 | NTS | DBLa | CIDRa | DBLd |  |  |  |  |  |  |  |
|  | 16 | NTS | DBLa | CIDRa | DBLd | CIDRb |  |  |  |  |  |  |
|  | 1 | NTS | DBLa | CIDRa | DBLd | CIDRb | DBLg | DBLz |  |  |  |  |
|  | 7 | NTS | DBLa | CIDRa | DBLd | CIDRg |  |  |  |  |  |  |
|  | 1 | NTS | DBLa | CIDRa | DBLg | DBLd | CIDRg | DBLe | DBLz | DBLe |  |  |
|  | 1 | NTS | DBLa | CIDRb | DBLg | DBLe | DBLz | DBLe |  |  |  |  |
|  | 1 | NTS | DBLa | CIDRd | DBLb | DBLg | DBLd | CIDRb |  |  |  |  |
|  | 1 | NTS | DBLa | CIDRd | DBLg | DBLd | CIDRb | DBLb |  |  |  |  |
|  | 1 | NTS | DBLa | CIDRg |  |  |  |  |  |  |  |  |
|  | 1 | NTS | DBLa | CIDRg | DBLg | DBLg | DBLd | CIDRb |  |  |  |  |
|  | 3 | NTS | DBLa | DBLe |  |  |  |  |  |  |  |  |
|  | 1 | NTSpam | DBLpam1 | DBLpam2 | CIDRpam | DBLpam3 | DBLe | DBLe | DBLe |  |  |  |
|  |  |  |  |  |  |  |  |  |  |  |  |  |
|  | **Isolate 377_1** | | | | | | | | | | | |
|  | 1 | CIDRa |  |  |  |  |  |  |  |  |  |  |
|  | 1 | CIDRa | DBLb | DBLg | DBLz | DBLe |  |  |  |  |  |  |
|  | 2 | CIDRa | DBLd | CIDRb |  |  |  |  |  |  |  |  |
|  | 2 | CIDRg |  |  |  |  |  |  |  |  |  |  |
|  | 1 | CIDRpam | DBLpam3 | DBLe | DBLe | DBLe |  |  |  |  |  |  |
|  | 1 | DBLa | CIDRa |  |  |  |  |  |  |  |  |  |
|  | 1 | DBLa | CIDRa | DBLb | DBLg | DBLd | CIDRb |  |  |  |  |  |
|  | 1 | DBLa | CIDRa | DBLd | CIDRb |  |  |  |  |  |  |  |
|  | 1 | DBLb |  |  |  |  |  |  |  |  |  |  |
|  | 2 | DBLb | DBLb | DBLd | CIDRb |  |  |  |  |  |  |  |
|  | 1 | DBLb | DBLd | CIDRb |  |  |  |  |  |  |  |  |
|  | 1 | DBLg |  |  |  |  |  |  |  |  |  |  |
|  | 1 | DBLpam2 | CIDRpam | DBLpam3 | DBLe | DBLe | DBLe |  |  |  |  |  |
|  | 3 | NTS | DBLa |  |  |  |  |  |  |  |  |  |
|  | 1 | NTS | DBLa | CIDRa |  |  |  |  |  |  |  |  |
|  | 1 | NTS | DBLa | CIDRa | DBLb |  |  |  |  |  |  |  |
|  | 2 | NTS | DBLa | CIDRa | DBLb | DBLb | DBLg | DBLd | CIDRb |  |  |  |
|  | 1 | NTS | DBLa | CIDRa | DBLb | DBLd | CIDRb |  |  |  |  |  |
|  | 1 | NTS | DBLa | CIDRa | DBLb | DBLd | CIDRg | DBLg | DBLz |  |  |  |
|  | 2 | NTS | DBLa | CIDRa | DBLb | DBLg |  |  |  |  |  |  |
|  | 1 | NTS | DBLa | CIDRa | DBLb | DBLg | DBLd |  |  |  |  |  |
|  | 2 | NTS | DBLa | CIDRa | DBLb | DBLg | DBLd | CIDRb |  |  |  |  |
|  | 1 | NTS | DBLa | CIDRa | DBLb | DBLg | DBLd | CIDRd |  |  |  |  |
|  | 1 | NTS | DBLa | CIDRa | DBLb | DBLg | DBLd | CIDRd | DBLb | DBLg |  |  |
|  | 2 | NTS | DBLa | CIDRa | DBLb | DBLg | DBLd | CIDRg |  |  |  |  |
|  | 1 | NTS | DBLa | CIDRa | DBLb | DBLg | DBLd | CIDRg | DBLz | DBLe |  |  |
|  | 1 | NTS | DBLa | CIDRa | DBLb | DBLg | DBLe | DBLg | DBLz | DBLe |  |  |
|  | 3 | NTS | DBLa | CIDRa | DBLb | DBLg | DBLg | DBLd | CIDRb |  |  |  |
|  | 1 | NTS | DBLa | CIDRa | DBLb | DBLz | DBLe |  |  |  |  |  |
|  | 20 | NTS | DBLa | CIDRa | DBLd | CIDRb |  |  |  |  |  |  |
|  | 1 | NTS | DBLa | CIDRa | DBLd | CIDRb | DBLg | DBLz | DBLe |  |  |  |
|  | 1 | NTS | DBLa | CIDRa | DBLd | CIDRg |  |  |  |  |  |  |
|  | 1 | NTS | DBLa | CIDRa | DBLd | CIDRg | DBLe | DBLe | DBLe |  |  |  |
|  | 1 | NTS | DBLa | CIDRa | DBLg | DBLd | CIDRb |  |  |  |  |  |
|  | 2 | NTS | DBLa | CIDRa | DBLg | DBLz |  |  |  |  |  |  |
|  | 1 | NTS | DBLa | CIDRb | DBLg | DBLe | DBLz | DBLe |  |  |  |  |
|  | 1 | NTS | DBLa | CIDRg | DBLb | DBLg | DBLg | DBLz |  |  |  |  |
|  | 1 | NTS | DBLa | DBLd | CIDRb |  |  |  |  |  |  |  |
|  | 1 | NTS | DBLa | DBLd | CIDRg |  |  |  |  |  |  |  |
|  | 1 | NTS | DBLa | DBLe |  |  |  |  |  |  |  |  |
|  | 1 | NTSpam | DBLpam1 |  |  |  |  |  |  |  |  |  |
|  | 3 | NTSpam | DBLpam1 | DBLpam2 | CIDRpam | DBLpam3 | DBLe | DBLe |  |  |  |  |
|  |  |  |  |  |  |  |  |  |  |  |  |  |
|  | **Isolate 383_1** | | | | | | | | | | | |
|  | 1 | CIDRa | DBLb |  |  |  |  |  |  |  |  |  |
|  | 1 | CIDRa | DBLd |  |  |  |  |  |  |  |  |  |
|  | 10 | CIDRa | DBLd | CIDRb |  |  |  |  |  |  |  |  |
|  | 1 | CIDRa | DBLd | CIDRb | DBLg | DBLz | DBLe |  |  |  |  |  |
|  | 1 | CIDRa | DBLd | CIDRg | DBLb | DBLg |  |  |  |  |  |  |
|  | 3 | CIDRb |  |  |  |  |  |  |  |  |  |  |
|  | 1 | CIDRb | DBLz | DBLe |  |  |  |  |  |  |  |  |
|  | 1 | DBLa | CIDRa | DBLb | DBLd | CIDRb |  |  |  |  |  |  |
|  | 1 | DBLb | DBLb |  |  |  |  |  |  |  |  |  |
|  | 1 | DBLb | DBLb | DBLb | DBLd | CIDRb |  |  |  |  |  |  |
|  | 1 | DBLb | DBLb | DBLd | CIDRb |  |  |  |  |  |  |  |
|  | 1 | DBLd | CIDRb |  |  |  |  |  |  |  |  |  |
|  | 1 | DBLd | CIDRg |  |  |  |  |  |  |  |  |  |
|  | 1 | DBLe |  |  |  |  |  |  |  |  |  |  |
|  | 2 | DBLg |  |  |  |  |  |  |  |  |  |  |
|  | 1 | DBLg | DBLe | DBLz | DBLe |  |  |  |  |  |  |  |
|  | 1 | NTS | DBLa |  |  |  |  |  |  |  |  |  |
|  | 3 | NTS | DBLa | CIDRa |  |  |  |  |  |  |  |  |
|  | 1 | NTS | DBLa | CIDRa | DBLb | DBLb | DBLd | CIDRb |  |  |  |  |
|  | 4 | NTS | DBLa | CIDRa | DBLb | DBLd | CIDRb |  |  |  |  |  |
|  | 1 | NTS | DBLa | CIDRa | DBLb | DBLd | CIDRb | DBLg | DBLz |  |  |  |
|  | 1 | NTS | DBLa | CIDRa | DBLb | DBLd | CIDRb | DBLg | DBLz | DBLe |  |  |
|  | 1 | NTS | DBLa | CIDRa | DBLb | DBLg |  |  |  |  |  |  |
|  | 1 | NTS | DBLa | CIDRa | DBLb | DBLg | DBLd | CIDRb |  |  |  |  |
|  | 1 | NTS | DBLa | CIDRa | DBLb | DBLg | DBLd | CIDRb | DBLb | DBLg |  |  |
|  | 1 | NTS | DBLa | CIDRa | DBLb | DBLg | DBLd | CIDRg |  |  |  |  |
|  | 1 | NTS | DBLa | CIDRa | DBLb | DBLg | DBLe | DBLd | CIDRb |  |  |  |
|  | 1 | NTS | DBLa | CIDRa | DBLb | DBLg | DBLe | DBLg | DBLz | DBLe |  |  |
|  | 1 | NTS | DBLa | CIDRa | DBLb | DBLg | DBLg | DBLd | CIDRg |  |  |  |
|  | 1 | NTS | DBLa | CIDRa | DBLb | DBLg | DBLg | DBLg |  |  |  |  |
|  | 2 | NTS | DBLa | CIDRa | DBLb | DBLg | DBLz | DBLe |  |  |  |  |
|  | 1 | NTS | DBLa | CIDRa | DBLd |  |  |  |  |  |  |  |
|  | 19 | NTS | DBLa | CIDRa | DBLd | CIDRb |  |  |  |  |  |  |
|  | 1 | NTS | DBLa | CIDRa | DBLd | CIDRb | DBLg | DBLz |  |  |  |  |
|  | 5 | NTS | DBLa | CIDRa | DBLd | CIDRg |  |  |  |  |  |  |
|  | 1 | NTS | DBLa | CIDRa | DBLd | CIDRg | DBLz | DBLe |  |  |  |  |
|  | 1 | NTS | DBLa | CIDRa | DBLe | DBLe | DBLe |  |  |  |  |  |
|  | 1 | NTS | DBLa | CIDRa | DBLg | DBLg | DBLz | DBLe |  |  |  |  |
|  | 1 | NTS | DBLa | CIDRd | DBLg | DBLd | CIDRb | DBLb |  |  |  |  |
|  | 1 | NTS | DBLa | DBLg | DBLe | DBLz | DBLe |  |  |  |  |  |
|  | 2 | NTSpam | DBLpam1 | DBLpam2 | CIDRpam | DBLpam3 | DBLe | DBLe | DBLe |  |  |  |
|  |  |  |  |  |  |  |  |  |  |  |  |  |
|  | **Isolate 397_1** | | | | | | | | | | | |
|  | 6 | CIDRa |  |  |  |  |  |  |  |  |  |  |
|  | 1 | CIDRa | DBLb |  |  |  |  |  |  |  |  |  |
|  | 1 | CIDRa | DBLb | DBLg | DBLd | CIDRb |  |  |  |  |  |  |
|  | 1 | CIDRa | DBLb | DBLg | DBLz |  |  |  |  |  |  |  |
|  | 1 | CIDRa | DBLd |  |  |  |  |  |  |  |  |  |
|  | 1 | CIDRa | DBLd | CIDRb |  |  |  |  |  |  |  |  |
|  | 1 | CIDRa | DBLd | CIDRb | DBLg |  |  |  |  |  |  |  |
|  | 3 | CIDRa | DBLd | CIDRg |  |  |  |  |  |  |  |  |
|  | 6 | CIDRb |  |  |  |  |  |  |  |  |  |  |
|  | 1 | CIDRg |  |  |  |  |  |  |  |  |  |  |
|  | 1 | DBLa |  |  |  |  |  |  |  |  |  |  |
|  | 1 | DBLa | CIDRa | DBLb | DBLg | DBLd | CIDRb |  |  |  |  |  |
|  | 1 | DBLa | CIDRa | DBLb | DBLg | DBLg | DBLd | CIDRb | DBLb | DBLg |  |  |
|  | 2 | DBLb |  |  |  |  |  |  |  |  |  |  |
|  | 1 | DBLb | DBLb | DBLb | DBLd | CIDRg |  |  |  |  |  |  |
|  | 1 | DBLb | DBLb | DBLd | CIDRb |  |  |  |  |  |  |  |
|  | 1 | DBLb | DBLb | DBLg | DBLd | CIDRb |  |  |  |  |  |  |
|  | 1 | DBLb | DBLd | CIDRg |  |  |  |  |  |  |  |  |
|  | 1 | DBLb | DBLg | DBLd | CIDRg | DBLe | DBLe |  |  |  |  |  |
|  | 1 | DBLb | DBLg | DBLe | DBLg | DBLz | DBLe |  |  |  |  |  |
|  | 1 | DBLd |  |  |  |  |  |  |  |  |  |  |
|  | 5 | DBLd | CIDRb |  |  |  |  |  |  |  |  |  |
|  | 2 | DBLd | CIDRg |  |  |  |  |  |  |  |  |  |
|  | 2 | DBLe |  |  |  |  |  |  |  |  |  |  |
|  | 1 | DBLg |  |  |  |  |  |  |  |  |  |  |
|  | 2 | DBLg | DBLz | DBLe |  |  |  |  |  |  |  |  |
|  | 1 | DBLz |  |  |  |  |  |  |  |  |  |  |
|  | 5 | NTS |  |  |  |  |  |  |  |  |  |  |
|  | 3 | NTS | DBLa |  |  |  |  |  |  |  |  |  |
|  | 6 | NTS | DBLa | CIDRa |  |  |  |  |  |  |  |  |
|  | 4 | NTS | DBLa | CIDRa | DBLb |  |  |  |  |  |  |  |
|  | 1 | NTS | DBLa | CIDRa | DBLb | DBLb |  |  |  |  |  |  |
|  | 1 | NTS | DBLa | CIDRa | DBLb | DBLb | DBLg | DBLd | CIDRb |  |  |  |
|  | 3 | NTS | DBLa | CIDRa | DBLb | DBLd | CIDRb |  |  |  |  |  |
|  | 1 | NTS | DBLa | CIDRa | DBLb | DBLd | CIDRb | DBLz | DBLe |  |  |  |
|  | 3 | NTS | DBLa | CIDRa | DBLb | DBLg |  |  |  |  |  |  |
|  | 2 | NTS | DBLa | CIDRa | DBLb | DBLg | DBLd |  |  |  |  |  |
|  | 2 | NTS | DBLa | CIDRa | DBLb | DBLg | DBLd | CIDRb |  |  |  |  |
|  | 1 | NTS | DBLa | CIDRa | DBLb | DBLg | DBLd | CIDRb | DBLb | DBLg |  |  |
|  | 1 | NTS | DBLa | CIDRa | DBLb | DBLg | DBLd | CIDRg |  |  |  |  |
|  | 1 | NTS | DBLa | CIDRa | DBLb | DBLg | DBLg | DBLg | DBLz | DBLe |  |  |
|  | 1 | NTS | DBLa | CIDRa | DBLb | DBLg | DBLz |  |  |  |  |  |
|  | 3 | NTS | DBLa | CIDRa | DBLb | DBLg | DBLz | DBLe |  |  |  |  |
|  | 1 | NTS | DBLa | CIDRa | DBLd |  |  |  |  |  |  |  |
|  | 9 | NTS | DBLa | CIDRa | DBLd | CIDRb |  |  |  |  |  |  |
|  | 2 | NTS | DBLa | CIDRa | DBLd | CIDRb | DBLb | DBLg |  |  |  |  |
|  | 1 | NTS | DBLa | CIDRa | DBLd | CIDRg |  |  |  |  |  |  |
|  | 1 | NTS | DBLa | CIDRa | DBLd | CIDRg | DBLz | DBLe |  |  |  |  |
|  | 1 | NTS | DBLa | CIDRa | DBLg | DBLd | CIDRg | DBLg | DBLe |  |  |  |
|  | 1 | NTS | DBLa | CIDRa | DBLg | DBLd | CIDRg | DBLz | DBLe |  |  |  |
|  | 1 | NTS | DBLa | CIDRa | DBLg | DBLg | DBLz | DBLe |  |  |  |  |
|  | 1 | NTS | DBLa | CIDRb | DBLb |  |  |  |  |  |  |  |
|  | 2 | NTS | DBLa | CIDRd | DBLb | DBLg | DBLd | CIDRg | DBLz | DBLe |  |  |
|  | 2 | NTS | DBLa | DBLe |  |  |  |  |  |  |  |  |
|  | 1 | NTS | DBLe |  |  |  |  |  |  |  |  |  |
|  | 1 | NTSpam | DBLpam1 | DBLpam2 | CIDRpam | DBLpam3 | DBLe | DBLe | DBLe |  |  |  |
|  |  |  |  |  |  |  |  |  |  |  |  |  |
|  | **Isolate 398_1** | | | | | | | | | | | |
|  | 1 | CIDRa | DBLb | DBLb |  |  |  |  |  |  |  |  |
|  | 2 | CIDRa | DBLd | CIDRb |  |  |  |  |  |  |  |  |
|  | 1 | CIDRa | DBLd | CIDRg |  |  |  |  |  |  |  |  |
|  | 3 | CIDRb |  |  |  |  |  |  |  |  |  |  |
|  | 1 | CIDRg | DBLe | DBLz | DBLe |  |  |  |  |  |  |  |
|  | 2 | DBLb |  |  |  |  |  |  |  |  |  |  |
|  | 1 | DBLb | DBLb | DBLb | DBLd | CIDRg |  |  |  |  |  |  |
|  | 1 | DBLb | DBLd | CIDRb |  |  |  |  |  |  |  |  |
|  | 1 | DBLb | DBLg | DBLd | CIDRb | DBLe | DBLg |  |  |  |  |  |
|  | 1 | DBLb | DBLg | DBLe | DBLg | DBLz | DBLe |  |  |  |  |  |
|  | 1 | DBLd | CIDRb |  |  |  |  |  |  |  |  |  |
|  | 1 | DBLg |  |  |  |  |  |  |  |  |  |  |
|  | 1 | DBLg | DBLd | CIDRg | DBLe | DBLe | DBLe |  |  |  |  |  |
|  | 1 | NTS | DBLa |  |  |  |  |  |  |  |  |  |
|  | 2 | NTS | DBLa | CIDRa |  |  |  |  |  |  |  |  |
|  | 1 | NTS | DBLa | CIDRa | DBLb | DBLb |  |  |  |  |  |  |
|  | 1 | NTS | DBLa | CIDRa | DBLb | DBLd |  |  |  |  |  |  |
|  | 1 | NTS | DBLa | CIDRa | DBLb | DBLd | CIDRb |  |  |  |  |  |
|  | 1 | NTS | DBLa | CIDRa | DBLb | DBLe |  |  |  |  |  |  |
|  | 7 | NTS | DBLa | CIDRa | DBLb | DBLg | DBLd | CIDRb |  |  |  |  |
|  | 1 | NTS | DBLa | CIDRa | DBLb | DBLg | DBLd | CIDRb | DBLb | DBLg |  |  |
|  | 1 | NTS | DBLa | CIDRa | DBLb | DBLg | DBLd | CIDRg |  |  |  |  |
|  | 1 | NTS | DBLa | CIDRa | DBLb | DBLg | DBLe | DBLg | DBLd | CIDRb |  |  |
|  | 2 | NTS | DBLa | CIDRa | DBLb | DBLg | DBLg | DBLd | CIDRb |  |  |  |
|  | 4 | NTS | DBLa | CIDRa | DBLb | DBLg | DBLz |  |  |  |  |  |
|  | 1 | NTS | DBLa | CIDRa | DBLb | DBLg | DBLz | DBLe |  |  |  |  |
|  | 21 | NTS | DBLa | CIDRa | DBLd | CIDRb |  |  |  |  |  |  |
|  | 1 | NTS | DBLa | CIDRa | DBLd | CIDRb | DBLg | DBLz |  |  |  |  |
|  | 4 | NTS | DBLa | CIDRa | DBLd | CIDRg |  |  |  |  |  |  |
|  | 1 | NTS | DBLa | CIDRa | DBLd | CIDRg | DBLe | DBLz | DBLe |  |  |  |
|  | 1 | NTS | DBLa | CIDRa | DBLg | DBLd | CIDRb |  |  |  |  |  |
|  | 1 | NTS | DBLa | CIDRa | DBLg | DBLd | CIDRg |  |  |  |  |  |
|  | 1 | NTS | DBLa | CIDRa | DBLg | DBLz | DBLe |  |  |  |  |  |
|  | 1 | NTS | DBLa | CIDRd | DBLb | DBLg | DBLe | DBLz | DBLe |  |  |  |
|  | 4 | NTS | DBLa | DBLe |  |  |  |  |  |  |  |  |
|  | 1 | NTS | DBLa | DBLg | DBLg | DBLd | CIDRg | DBLe | DBLz | DBLe |  |  |
|  | 1 | NTSpam | DBLpam1 | DBLpam2 | CIDRpam | DBLpam3 | DBLe | DBLe | DBLe |  |  |  |
|  |  |  |  |  |  |  |  |  |  |  |  |  |
|  | **Isolate 58_1** | | | | | | | | | | | |
|  | 2 | CIDRa | DBLb |  |  |  |  |  |  |  |  |  |
|  | 1 | CIDRa | DBLb | DBLb |  |  |  |  |  |  |  |  |
|  | 2 | CIDRa | DBLd |  |  |  |  |  |  |  |  |  |
|  | 4 | CIDRa | DBLd | CIDRb |  |  |  |  |  |  |  |  |
|  | 3 | CIDRb |  |  |  |  |  |  |  |  |  |  |
|  | 1 | CIDRb | DBLb | DBLg |  |  |  |  |  |  |  |  |
|  | 1 | CIDRb | DBLg |  |  |  |  |  |  |  |  |  |
|  | 2 | DBLa |  |  |  |  |  |  |  |  |  |  |
|  | 1 | DBLa | CIDRa | DBLd |  |  |  |  |  |  |  |  |
|  | 6 | DBLb |  |  |  |  |  |  |  |  |  |  |
|  | 2 | DBLb | DBLb |  |  |  |  |  |  |  |  |  |
|  | 1 | DBLb | DBLb | DBLb | DBLd |  |  |  |  |  |  |  |
|  | 2 | DBLb | DBLb | DBLd | CIDRb |  |  |  |  |  |  |  |
|  | 1 | DBLb | DBLb | DBLg | DBLd | CIDRb |  |  |  |  |  |  |
|  | 7 | DBLb | DBLd | CIDRb |  |  |  |  |  |  |  |  |
|  | 1 | DBLb | DBLg |  |  |  |  |  |  |  |  |  |
|  | 8 | DBLd | CIDRb |  |  |  |  |  |  |  |  |  |
|  | 1 | DBLd | CIDRg |  |  |  |  |  |  |  |  |  |
|  | 1 | DBLe |  |  |  |  |  |  |  |  |  |  |
|  | 1 | DBLe | DBLe |  |  |  |  |  |  |  |  |  |
|  | 1 | DBLe | DBLg | DBLz | DBLe |  |  |  |  |  |  |  |
|  | 1 | DBLg | DBLd | CIDRb |  |  |  |  |  |  |  |  |
|  | 1 | DBLg | DBLg |  |  |  |  |  |  |  |  |  |
|  | 1 | DBLg | DBLg | DBLg | DBLd | CIDRb |  |  |  |  |  |  |
|  | 2 | DBLg | DBLz | DBLe |  |  |  |  |  |  |  |  |
|  | 1 | DBLz |  |  |  |  |  |  |  |  |  |  |
|  | 1 | DBLz | DBLe |  |  |  |  |  |  |  |  |  |
|  | 1 | NTS |  |  |  |  |  |  |  |  |  |  |
|  | 5 | NTS | DBLa |  |  |  |  |  |  |  |  |  |
|  | 6 | NTS | DBLa | CIDRa |  |  |  |  |  |  |  |  |
|  | 5 | NTS | DBLa | CIDRa | DBLb |  |  |  |  |  |  |  |
|  | 1 | NTS | DBLa | CIDRa | DBLb | DBLb | DBLd | CIDRb |  |  |  |  |
|  | 1 | NTS | DBLa | CIDRa | DBLb | DBLb | DBLg | DBLd |  |  |  |  |
|  | 1 | NTS | DBLa | CIDRa | DBLb | DBLd |  |  |  |  |  |  |
|  | 4 | NTS | DBLa | CIDRa | DBLb | DBLd | CIDRb |  |  |  |  |  |
|  | 1 | NTS | DBLa | CIDRa | DBLb | DBLg |  |  |  |  |  |  |
|  | 3 | NTS | DBLa | CIDRa | DBLb | DBLg | DBLd | CIDRb |  |  |  |  |
|  | 1 | NTS | DBLa | CIDRa | DBLb | DBLg | DBLd | CIDRd |  |  |  |  |
|  | 1 | NTS | DBLa | CIDRa | DBLb | DBLg | DBLg | DBLd | CIDRb |  |  |  |
|  | 1 | NTS | DBLa | CIDRa | DBLb | DBLz | DBLe |  |  |  |  |  |
|  | 2 | NTS | DBLa | CIDRa | DBLd |  |  |  |  |  |  |  |
|  | 15 | NTS | DBLa | CIDRa | DBLd | CIDRb |  |  |  |  |  |  |
|  | 1 | NTS | DBLa | CIDRa | DBLd | CIDRb | DBLg |  |  |  |  |  |
|  | 1 | NTS | DBLa | CIDRa | DBLd | CIDRb | DBLz | DBLe |  |  |  |  |
|  | 4 | NTS | DBLa | CIDRa | DBLd | CIDRg |  |  |  |  |  |  |
|  | 1 | NTS | DBLa | CIDRa | DBLd | CIDRg | DBLz | DBLe |  |  |  |  |
|  | 1 | NTS | DBLa | CIDRa | DBLe | DBLe | DBLe |  |  |  |  |  |
|  | 1 | NTS | DBLa | CIDRa | DBLg | DBLg | DBLz | DBLe |  |  |  |  |
|  | 1 | NTS | DBLa | CIDRb | DBLg | DBLe | DBLz | DBLe |  |  |  |  |
|  | 1 | NTS | DBLa | CIDRd | DBLg |  |  |  |  |  |  |  |
|  | 1 | NTS | DBLa | CIDRg |  |  |  |  |  |  |  |  |
|  | 1 | NTS | DBLa | CIDRg | DBLg | DBLd | CIDRg | DBLe | DBLe | DBLe |  |  |
|  | 1 | NTS | DBLa | DBLd |  |  |  |  |  |  |  |  |
|  | 2 | NTSpam | DBLpam1 | DBLpam2 | CIDRpam | DBLpam3 | DBLe | DBLe | DBLe |  |  |  |
|  |  |  |  |  |  |  |  |  |  |  |  |  |
|  | **PFCLIN** | | | | | | | | | | | |
|  | 1 | CIDRb | ATS |  |  |  |  |  |  |  |  |  |
|  | 1 | DBLa | CIDRa | DBLd | CIDRb |  |  |  |  |  |  |  |
|  | 1 | DBLb | DBLb | DBLd | CIDRb | ATS |  |  |  |  |  |  |
|  | 1 | DBLb | DBLg |  |  |  |  |  |  |  |  |  |
|  | 2 | DBLd | CIDRb |  |  |  |  |  |  |  |  |  |
|  | 1 | NTS | DBLa |  |  |  |  |  |  |  |  |  |
|  | 5 | NTS | DBLa | CIDRa |  |  |  |  |  |  |  |  |
|  | 1 | NTS | DBLa | CIDRa | DBLb | DBLb |  |  |  |  |  |  |
|  | 1 | NTS | DBLa | CIDRa | DBLb | DBLd | CIDRd | ATS |  |  |  |  |
|  | 2 | NTS | DBLa | CIDRa | DBLb | DBLd | CIDRg | ATS |  |  |  |  |
|  | 1 | NTS | DBLa | CIDRa | DBLb | DBLd | CIDRg | DBLg | DBLz | ATS |  |  |
|  | 1 | NTS | DBLa | CIDRa | DBLb | DBLe | DBLd |  |  |  |  |  |
|  | 2 | NTS | DBLa | CIDRa | DBLb | DBLg | ATS |  |  |  |  |  |
|  | 1 | NTS | DBLa | CIDRa | DBLb | DBLg | DBLd | CIDRb |  |  |  |  |
|  | 1 | NTS | DBLa | CIDRa | DBLb | DBLg | DBLd | CIDRb | ATS |  |  |  |
|  | 1 | NTS | DBLa | CIDRa | DBLb | DBLg | DBLe | DBLg | DBLz | DBLe | ATS |  |
|  | 1 | NTS | DBLa | CIDRa | DBLb | DBLg | DBLg |  |  |  |  |  |
|  | 1 | NTS | DBLa | CIDRa | DBLb | DBLg | DBLg | DBLd | CIDRg | ATS |  |  |
|  | 1 | NTS | DBLa | CIDRa | DBLb | DBLg | DBLz |  |  |  |  |  |
|  | 1 | NTS | DBLa | CIDRa | DBLb | DBLg | DBLz | DBLe |  |  |  |  |
|  | 1 | NTS | DBLa | CIDRa | DBLb | DBLg | DBLz | DBLe | ATS |  |  |  |
|  | 1 | NTS | DBLa | CIDRa | DBLb | DBLz | DBLe | ATS |  |  |  |  |
|  | 2 | NTS | DBLa | CIDRa | DBLd |  |  |  |  |  |  |  |
|  | 8 | NTS | DBLa | CIDRa | DBLd | CIDRb |  |  |  |  |  |  |
|  | 6 | NTS | DBLa | CIDRa | DBLd | CIDRb | ATS |  |  |  |  |  |
|  | 5 | NTS | DBLa | CIDRa | DBLd | CIDRg | ATS |  |  |  |  |  |
|  | 1 | NTS | DBLa | CIDRa | DBLg |  |  |  |  |  |  |  |
|  | 1 | NTS | DBLa | CIDRa | DBLg | DBLd | CIDRg | DBLe | DBLz | DBLe | ATS |  |
|  | 1 | NTS | DBLa | CIDRa | DBLg | DBLg | DBLd | CIDRb |  |  |  |  |
|  | 2 | NTS | DBLa | CIDRa | DBLg | DBLz | DBLe | ATS |  |  |  |  |
|  | 1 | NTS | DBLa | CIDRb | DBLg | DBLd | CIDRb | DBLz | DBLe | ATS |  |  |
|  | 1 | NTS | DBLa | CIDRd | DBLb | DBLg | DBLg |  |  |  |  |  |
|  | 1 | NTS | DBLa | CIDRd | DBLg | DBLd | CIDRb | DBLb |  |  |  |  |
|  | 1 | NTS | DBLa | CIDRg | DBLb | DBLg | DBLz | DBLe | ATS |  |  |  |
|  | 1 | NTS | DBLa | DBLd | CIDRg | ATS |  |  |  |  |  |  |
|  | 1 | NTS | DBLa | DBLe | ATS |  |  |  |  |  |  |  |
|  | 1 | NTSpam | DBLpam1 | DBLpam2 | CIDRpam | DBLpam3 | DBLe | DBLe | DBLe |  |  |  |
|  | 1 | NTSpam | DBLpam1 | DBLpam2 | CIDRpam | DBLpam3 | DBLe | DBLe | DBLe | ATS |  |  |
|  |  |  |  |  |  |  |  |  |  |  |  |  |
|  | **3D7** | | | | | | | | | | | |
|  | 1 | NTS | DBLa | CIDRa | DBLb | DBLb | DBLd | CIDRg | ATS |  |  |  |
|  | 1 | NTS | DBLa | CIDRa | DBLb | DBLb | DBLg | DBLd | CIDRb | ATS |  |  |
|  | 2 | NTS | DBLa | CIDRa | DBLb | DBLd | CIDRb | ATS |  |  |  |  |
|  | 1 | NTS | DBLa | CIDRa | DBLb | DBLg | ATS |  |  |  |  |  |
|  | 1 | NTS | DBLa | CIDRa | DBLb | DBLg | DBLd | CIDRb | ATS |  |  |  |
|  | 1 | NTS | DBLa | CIDRa | DBLb | DBLg | DBLd | CIDRg | DBLe | DBLe | DBLe | ATS |
|  | 1 | NTS | DBLa | CIDRa | DBLb | DBLg | DBLg | DBLd | CIDRg | ATS |  |  |
|  | 2 | NTS | DBLa | CIDRa | DBLb | DBLg | DBLz | DBLe | ATS |  |  |  |
|  | 28 | NTS | DBLa | CIDRa | DBLd | CIDRb | ATS |  |  |  |  |  |
|  | 1 | NTS | DBLa | CIDRa | DBLd | CIDRb | DBLe | ATS |  |  |  |  |
|  | 13 | NTS | DBLa | CIDRa | DBLd | CIDRg | ATS |  |  |  |  |  |
|  | 1 | NTS | DBLa | CIDRa | DBLg | ATS |  |  |  |  |  |  |
|  | 1 | NTS | DBLa | CIDRa | DBLg | DBLd | CIDRb | ATS |  |  |  |  |
|  | 1 | NTS | DBLa | CIDRd | DBLb | DBLg | DBLd | CIDRb | DBLb | ATS |  |  |
|  | 1 | NTS | DBLa | CIDRd | DBLb | DBLg | DBLz | DBLe | ATS |  |  |  |
|  | 1 | NTS | DBLa | CIDRg | DBLg | DBLd | CIDRb | DBLb | ATS |  |  |  |
|  | 3 | NTS | DBLa | DBLe | ATS |  |  |  |  |  |  |  |
|  | 1 | NTSpam | DBLpam1 | DBLpam2 | CIDRpam | DBLpam3 | DBLe | DBLe | DBLe | ATS |  |  |
|  |  |  |  |  |  |  |  |  |  |  |  |  |
|  |  |  |  |  |  |  |  |  |  |  |  |  |

**Figure S1. Domain organization of PfEMP1 in each of 12 Malain isolates.** Domain content and structural domain architecture of annotated PfEMP1s from each of 12 clinical samples. PfEMP1s that contain the N-terminal sequence (NTS) are boxed. The number of each type of domain architecture is also shown.


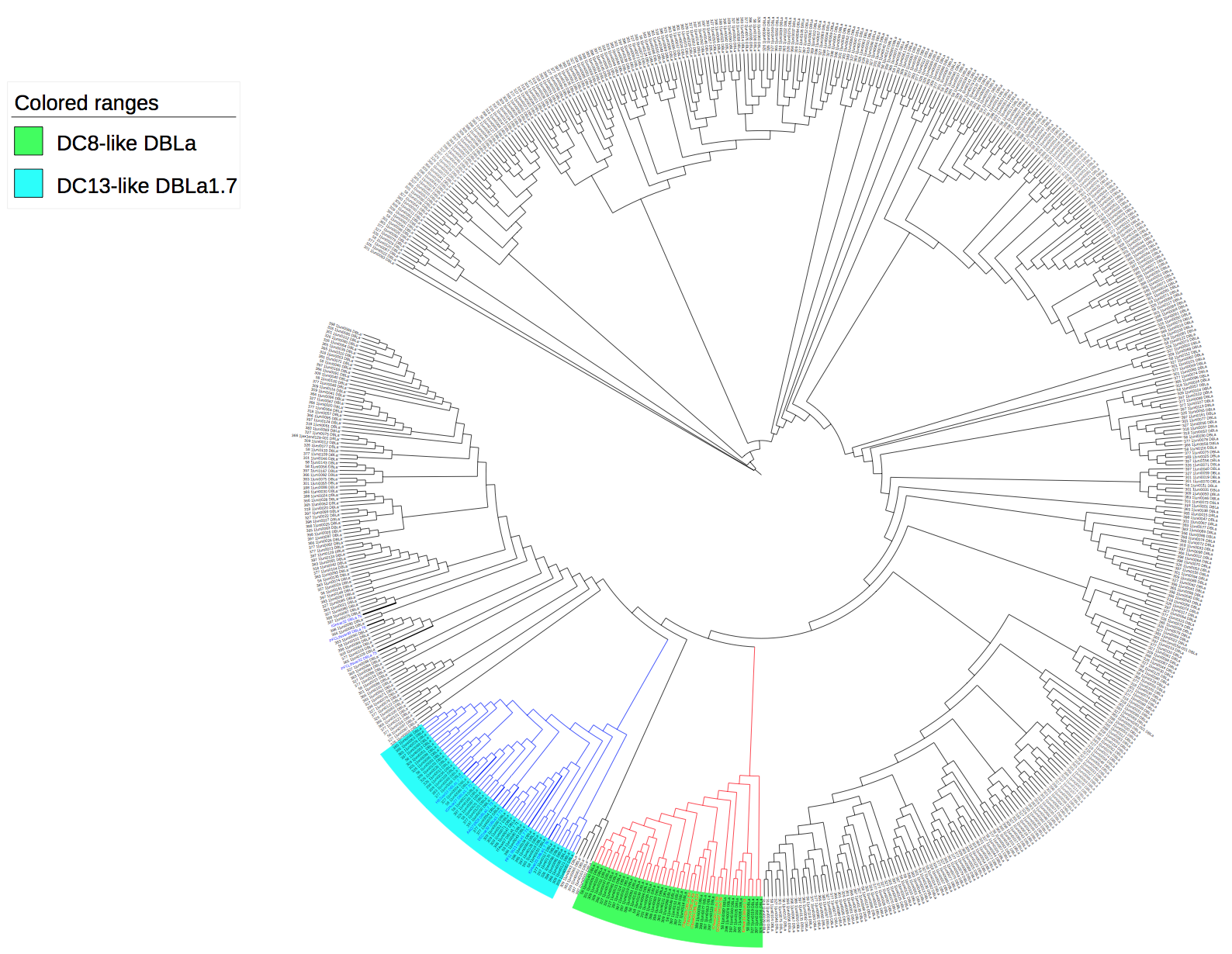
**Figure S2. Maximum likelihood phylogeny of DBLα domain sequences.** All domains annotated as DBLα from the 12 samples are shown. Color coded are DBLα sequences that clustered with known domain cassettes DC8 (red label highlighted in green) and DC13 (blue label highlighted in cyan). DBLα2 represents DC8-like DBLα, whereas DBLα1.7 defines DC13-like DBLα. The three sequences labeled in blue outside of the highlighted clusters were use as non-DC8 and non-DC13 controls.

**Figure S3. Maximum likelihood phylogeny of CIDRα domain sequences.** All domains annotated as CIDRα from the 12 samples are shown. CIDRα sequences that clustered with known domain cassettes DC8 (red branches highlighted in green) and DC13 (blue branches highlighted in cyan) are shown as well. CIDRα1.1 represents DC8-like CIDRα, whereas CIDRα1.4 defines DC13-like CIDRα.


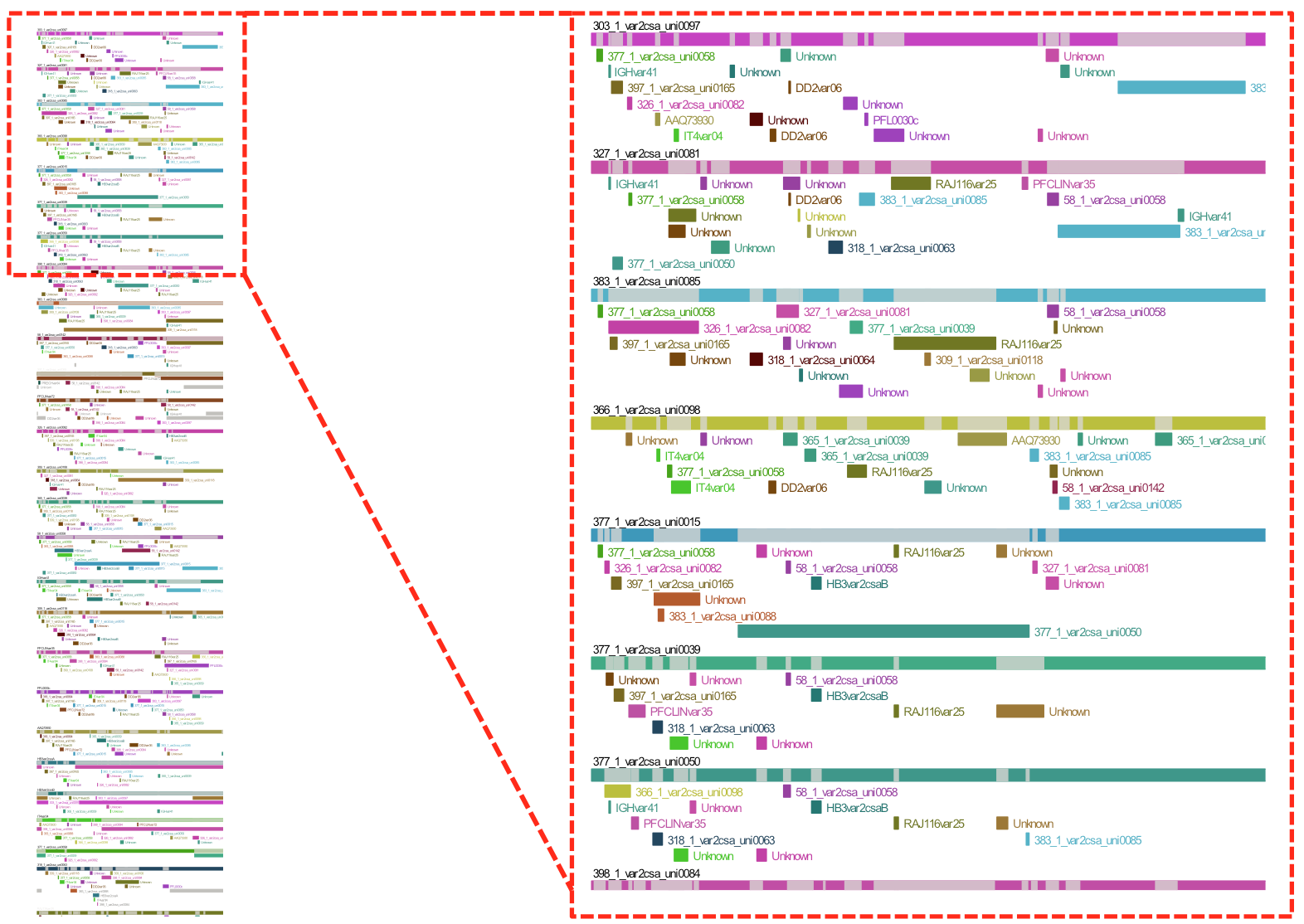


**Figure S4. Detection and visualization of recombination events within and between *var2csa* sequences.** The left panel represents the recombination events detected between *var2csa* sequences from geographically close and distant parasites using the RDP4 program and the right panel is a zoom the first eight sequences of the 12 Malian samples. The full-length exon 1 on *var2csa* is denoted by the long horizontal bar. Below each *var2csa* are depicted recombinant segments and the names of the sequences they recombined with. For example, recombination events occurred between the sample shown on top (303_1_var2csa_0097) and 377_1_var2csa_0068, IGHvar41, DD2var06, etc.

**Figure S5 - Phylogenetic tree of reconstructed *var2csa* exon 1 sequences**. The tree is inferred from the 12 Malian samples (19 sequences) and 12 sequences from VarDom database using maximum likelihood. Complete sequences are used, with no regard to possible recombination.
